# Rhodium-catalyzed oxidative amidation of allylic alcohols and aldehydes: effective conversion of amines and anilines into amides†
†Electronic supplementary information (ESI) available. See DOI: 10.1039/c5sc03103f


**DOI:** 10.1039/c5sc03103f

**Published:** 2015-10-27

**Authors:** Zhao Wu, Kami L. Hull

**Affiliations:** a University of Illinois , Urbana-Champaign , Department of Chemistry , 600 S. Mathews , Urbana , IL 61820 , USA . Email: kamihull@illinois.edu

## Abstract

The rhodium-catalyzed oxidative amidation of allylic alcohols and aldehydes is reported. *In situ* generated [(BINAP)Rh]BF_4_ catalyzes the one-pot isomerization/oxidative amidation of allylic alcohols or direct amidation of aldehydes using acetone or styrene as the hydrogen acceptor. The conditions are general, affording good to excellent yields with a wide array of amine and aniline nucleophiles, and chemoselective, other alcohols do not participate in the oxidation reaction. Utilization of biphasic conditions is critical, as they promote an equilibrium between the imine/enamine byproducts and the hemiaminal, which can undergo oxidation to the amide.

## Abstract 2

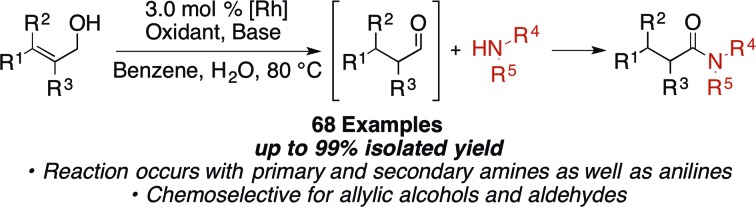
The rhodium-catalyzed oxidative amidation of allylic alcohols and aldehydes is reported.

## Introduction

**Scheme 1:**
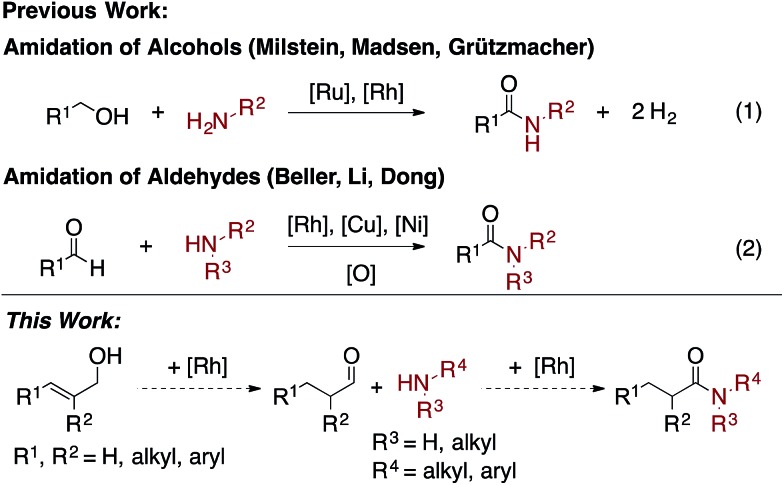
Transition metal-catalyzed oxidative amidation of alcohols and aldehydes.

Amides are a common functionality found throughout natural products, pharmaceuticals, agrochemicals, and organic materials.^1^ Approaches have been developed for the coupling of carboxylic acids and amines to generate amides in high yields.^2^ While these reactions are versatile and widely employed, there are significant drawbacks associated with them: they generate super-stoichiometric quantities of high molecular weight byproducts and are not functional group tolerant.^3^ The transition metal-catalyzed oxidative amidation of alcohols^4^ and aldehydes^5^ is a promising alternative to traditional coupling methods,^6^,^7^ as it allows for the generation of amides along with molecular hydrogen as the only by-product (Scheme 1). For example, Misltein and Madsen reported an acceptorless oxidative amidation of alcohols using Ru catalysis with pincer4*c* and NHC4*e* ligands, respectively. Although no hydrogen acceptor is needed, substrate scopes are limited to unhindered alcohols and amines. Secondary cyclic amines require a less-hindered pincer ligand8*b* and acyclic amines are still challenging.^8^ Moreover, elevated temperatures (>110 °C) and refluxing solvents are needed to favor the evolution of H_2_ gas4c–e,i–l or transfer hydrogenation to ketones.4*f* Direct amidation of aryl aldehydes with external oxidants have been reported by Rh5*b* and Cu5*c* catalysis. However, yields are usually low when enolizable aldehydes are used due to the formation of amine byproducts.5*b* Recently, Dong reported a Ni-catalyzed C–H activation of aldehydes followed by coupling with alcohols or amines to form esters and amides respectively.5*i* A variety of aldehydes and amines are shown to be reactive, but an additional equivalent aldehyde or trifluoroacetophenone is required as the hydrogen acceptor. Herein, we report an easily accessible, chemoselective, and general catalytic method, which selectively couples allylic alcohols or aldehydes with aliphatic and aryl amines to form amides.

## Results and discussion

### Research hypothesis

In seeking to develop general conditions for amide bond formation, we proposed that allylic alcohols could serve as an aldehyde precursor.^9^,^10^ Such a system would be advantageous as allylic alcohols have been previously shown to isomerize to an aldehyde in the presence of a [Rh]-catalyst.^10^ Then, the *in situ* generated aldehydes could undergo a Rh-catalyzed oxidative amidation with an amine to afford the amide and a Rh–H. Subsequent H_2_ formation or transfer hydrogenation would regenerate the active catalyst species.4g,5b

### Optimization studies

Initial efforts focused on developing a Rh-catalyzed oxidative amidation of cinnamyl alcohol (**1a**) and *N*-methylpiperazine (**2a**). When they are combined with a cationic rhodium catalyst, the enamine (*E*)-1-methyl-4-(3-phenylprop-1-en-1-yl)piperazine (**4a**) predominates,^12^ suggesting that isomerization/condensation is significantly faster than the subsequent oxidation of the hemiaminal (*vide infra*). Moreover, reduced starting material **1h** was also observed, indicating that the cinnamyl alcohol was acting as the hydrogen acceptor.^12^

As such, two main challenges in optimization of the desired oxidative amidation reaction were to identify: (1) conditions that promote amide over enamine formation and (2) an oxidant that is selectively reduced over the allylic alcohol.

As summarized in Table 1, and further elaborated in Tables S1–S8,† a variety of reaction conditions were explored: varying solvent systems, bases, and oxidants. As enamine byproducts do not undergo the oxidative amidation, it was postulated that adding water to the reaction might promote reformation of the hemiaminal; indeed, the addition of an equal volume of H_2_O to non-polar solvents like benzene and toluene significantly suppressed the formation of the enamine **4a** (Table S3†) and favored the formation of the amide **3a** (Table 1, entries 1–4; Table S2†). Bases were next investigated: addition of CsOAc gave 56% yield of **3a**; stronger bases lead to lower yields of the desired amide, with KOH and Et_3_N affording **3a** in 24% and 41% yields with **1h** as the major byproduct (Table 1, entry 5–8; Table S4†). Next, hydrogen acceptors were investigated; styrene proved to be superior, over acetone, affording **3a** in 80% yield and **1h** in 9% yield (Table 1, entry 11). Cyclohexanone, norbornene and MMA lead to lower yields of **3a** (Table 1, entries 9–12; Table S4†). Finally, increasing the equivalents of styrene and CsOAc slightly improved yields and shortened the reaction time to 4 hours (Table 1, entries 13–16; Tables S5 and S7†).

**Table 1:** Selected optimization of oxidative amidation of allyl alcohol with secondary amine^*a*^

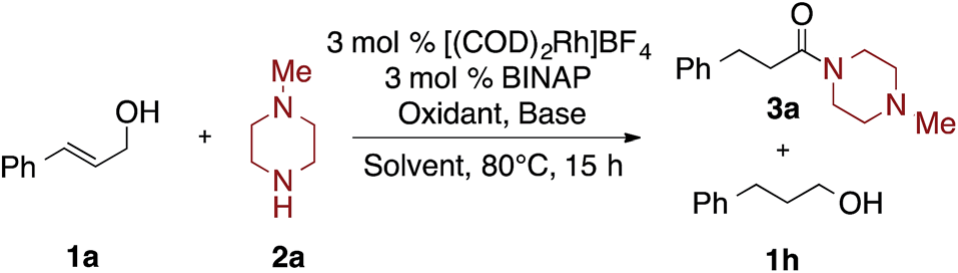					
Entry	Solvent	Base	Oxidant	% yield **3a**	% yield **1h**
1	DME	None	Acetone	9	0
2	DME/H_2_O	None	Acetone	14	0
3	C_6_H_6_/H_2_O	None	Acetone	42	20
4	Tol/H_2_O	None	Acetone	38	19
5	C_6_H_6_/H_2_O	KOH	Acetone	24	19
6	C_6_H_6_/H_2_O	NEt_3_	Acetone	41	19
7	C_6_H_6_/H_2_O	K_2_CO_3_	Acetone	51	24
8	C_6_H_6_/H_2_O	CsOAc	Acetone	56	22
9	C_6_H_6_/H_2_O	CsOAc	Cyclohexanone	63	22
10	C_6_H_6_/H_2_O	CsOAc	Norbornene	62	23
11	C_6_H_6_/H_2_O	CsOAc	Styrene	80	9
12	C_6_H_6_/H_2_O	CsOAc	MMA^*b*^	65	12
13	C_6_H_6_/H_2_O	CsOAc	Styrene^*c*^	68	20
14	C_6_H_6_/H_2_O	CsOAc	Styrene^*d*^	91	5
15	C_6_H_6_/H_2_O	CsOAc^*e*^	Styrene	84	10
16	C_6_H_6_/H_2_O	CsOAc^*f*^	Styrene	90	10

^*a*^Unless otherwise noted, reaction conditions are: allyl alcohol (0.25 mmol, 1.0 equiv.), amine (3.0 equiv.), base (1.5 equiv.), oxidant (3.0 equiv.), solvent (0.2 mL, 1.2 M), DI H_2_O (0.2 mL). Yields are determined by GC analysis and comparison to an internal standard.

^*b*^MMA = methyl methacrylate.

^*c*^1.0 equiv.

^*d*^5.0 equiv.

^*e*^2.0 equiv.

^*f*^2.5 equiv.

Interestingly, as seen in Table 2, under the optimized conditions benzyl amine gave only 11% yield of amide **3h** and 89% yield of imine **4h** (Table 2, entry 1). Fortunately, by changing the base and hydrogen acceptor to KOH and acetone, respectively, the desired amide **3h** is formed in 82% yield along with only 6% yield of **1h** (Table 2, entry 6).

**Table 2:** Selected optimization of oxidative amidation of allyl alcohol with primary amine^*a*^

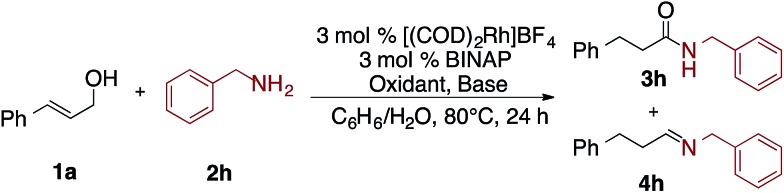					
Entry	Base	Oxidant	% yield **3h**	% yield **4h**	% yield **1h**
1	CsOAc	Styrene	11	89	0
2	Cs_2_CO_3_	Styrene	50	30	20
3	KOH	Styrene	51	19	30
4	KOH	Norbornene	56	10	26
5	KOH	NMO	48	6	37
6	KOH	Acetone	82	0	6
7	Cs_2_CO_3_	Acetone	60	11	18
8	KOH	Acetone^*b*^	84	0	4
9^*c*^	KOH	Acetone	15	3	0

^*a*^Unless otherwise noted, reaction conditions are: allyl alcohol (0.25 mmol, 1.0 equiv.), amine (3.0 equiv.), base (1.5 equiv.), oxidant (3.0 equiv.), solvent (0.2 mL, 1.2 M), DI H_2_O (0.2 mL). Yields are determined by GC analysis and comparison to an internal standard.

^*b*^5.0 equiv.

^*c*^No H_2_O added.

### Substrate scope

The optimization experiments suggest that the allylic alcohol rapidly converts into the aldehyde under the reaction conditions, which could then go on to form the amide. This suggested that under the optimized conditions, aldehydes should be effective substrates for the oxidative coupling reaction. Indeed, when 3-phenylpropanal (**5a**) is subjected to the optimized conditions amide **3a** is formed in a 76% yield, which is nearly identical to the 81% yield from the cinnamyl alcohol (Table 3). The scope of amines was then explored with both **1a** and **5a** (Table 3). Secondary cyclic amines, including 1-methylpiperazine, morpholine, piperidine, pyrrolidine, tetrahydroisoquinoline (**2a–2e**) and more sterically hindered acyclic amines, such as dimethyl amine (**2f**) and *N*-benzyl methyl amine (**2g**) are incorporated in very good, and nearly equivalent, yields from either the **1a** or **5a**. *cis*-Cinnamyl alcohol also undergoes the amidation reaction with morpholine to afford **3b**, although at a significantly reduced rate and in moderately reduced yields.^12^ Likewise, primary amines afforded **3h–3l** in good to excellent yields.

**Table 3:** Amine scope of allylic alcohol and aldehyde amidation^*a*^

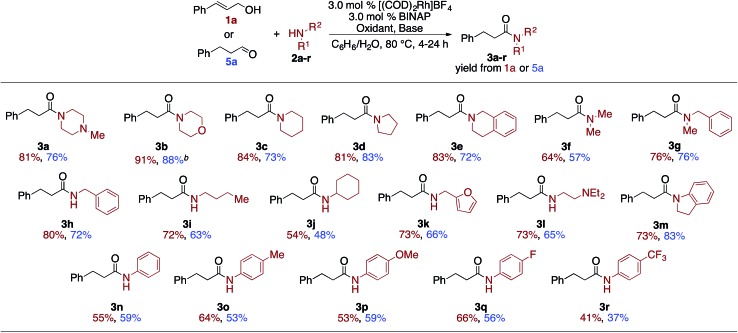

^*a*^Alcohol or aldehyde (0.25 mmol, 1.0 equiv.), amine (3.0 equiv.), [(COD)_2_Rh]BF_4_ (3.0 mol%), BINAP (3.0 mol%), oxidant (3.0–5.0 equiv.): styrene (2° amine and aniline) or acetone (1° amine), base (1.5–2.5 equiv.), benzene (0.2 mL, 1.2 M), DI H_2_O (0.2 mL).^12^

^*b*^82% yield was observed from (*Z*)-cinnamyl alcohol.^12^

The reaction is sensitive to steric hindrance of the amine, as cyclohexylamine affords 54%/48% yield of amide **3j** while *n*-butylamine affords amide **3i** in 72%/63% yield. Unlike other oxidative amidation processes,4a,d–l electron rich and electron poor aniline derivatives (**2m–2r**) were all effective nucleophiles in the coupling reaction without requiring higher temperatures or specialized reaction conditions.^11^ It is important to note, that for all substrates, the allylic alcohol and the aldehyde gave similar yields.

The allylic alcohols that undergo the oxidative amidation reaction were explored, as seen in Table 4. Functional groups such as ketones, esters, ethers, aryl bromides, and chlorides are tolerant under the standard conditions based on a chemical robustness screen.^12^ Products bearing active aryl bromides could be synthesized in good yields (**3ba**, **3ha**), allowing for facile subsequent coupling reactions to increase molecule complexity. 1,1-Di, 1,2-di-, tri-, and tetra-substituted allylic alcohols give the corresponding α- or/and β-branched secondary (**6i–6p**) and tertiary (**6a–6h**) amides in moderate to good yields with primary and secondary amines, respectively. Notably, allylic alcohols known to be slow to isomerize, such as cyclohex-1-en-1-ylmethanol (**1g**) afforded moderate yields (54%) of the oxidative amidation product **6f** after 24 hours.10*c* Notably, geraniol was coupled to afford **6g** and **6o** in 64% and 65% yield, respectively; neither reduction nor isomerization of the distal alkene was observed.

**Table 4:** Allylic alcohol scope for amidation^*a*^

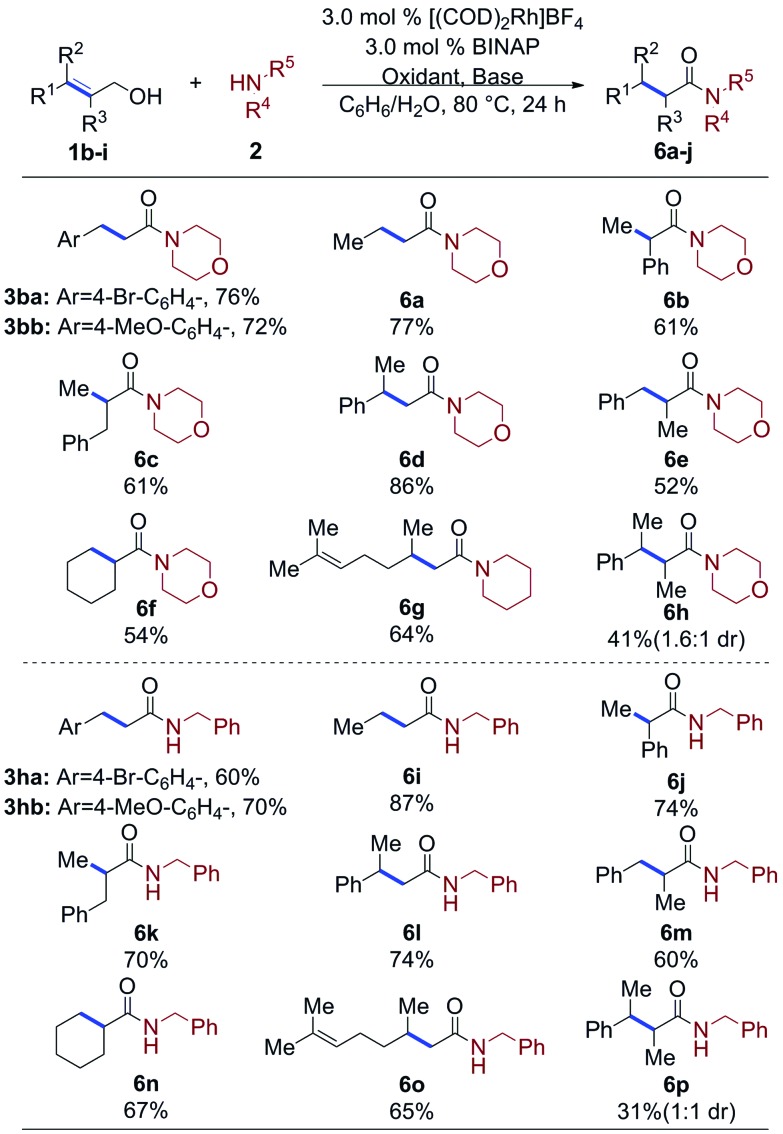

^*a*^Alcohol or aldehyde (0.25 mmol, 1.0 equiv.), amine (3.0 equiv.), [(COD)_2_Rh]BF_4_ (3.0 mol%), BINAP (3.0 mol%), oxidant (3.0–5.0 equiv.): styrene (2° amine) or acetone (1° amine benzene (0.2 mL, 1.2 M), DI H_2_O (0.2 mL).^12^

Tetra-substituted allylic alcohols react, affording **6h** and **6p** in 41% and 31% yield, respectively, along with unreacted starting material. Unfortunately, the diastereoselectivity of these reactions was poor, affording the amides in only 1.6 : 1 and 1 : 1 dr.

Finally, as seen in Table 5, the scope of aldehydes that undergo the oxidative amidation reaction was investigated. The reactions are tolerant of a variety of functionalities, including ethers (**7b**), acetals (**7c**), aryl bromides (**7d**), aryl fluorides (**7g**), trifluromethyls (**7i**), nitriles (**7h**), and heteroaromatics (**7j**). Benzaldehyde derivatives with electron donating groups, such as *p*-MeO, afford the desire amide in excellent yield; electron poor benzaldehydes, such as *p*-CN or *p*-F_3_C, undergo the oxidative amidation to afford **7h** and **7i**, albeit in reduced yields. Steric hindrance of the aldehyde did not affect its reactivity, as 2,6-dimethylbenzaldehyde afforded amide **7f** in 88% yield. Aliphatic aldehydes, which have proven challenging for other oxidative amidation reactions,5a–h also afford the desired amides in good to very good yields (**7k** and **6g**).

**Table 5:** Aldehyde scope for amidation^*a*^

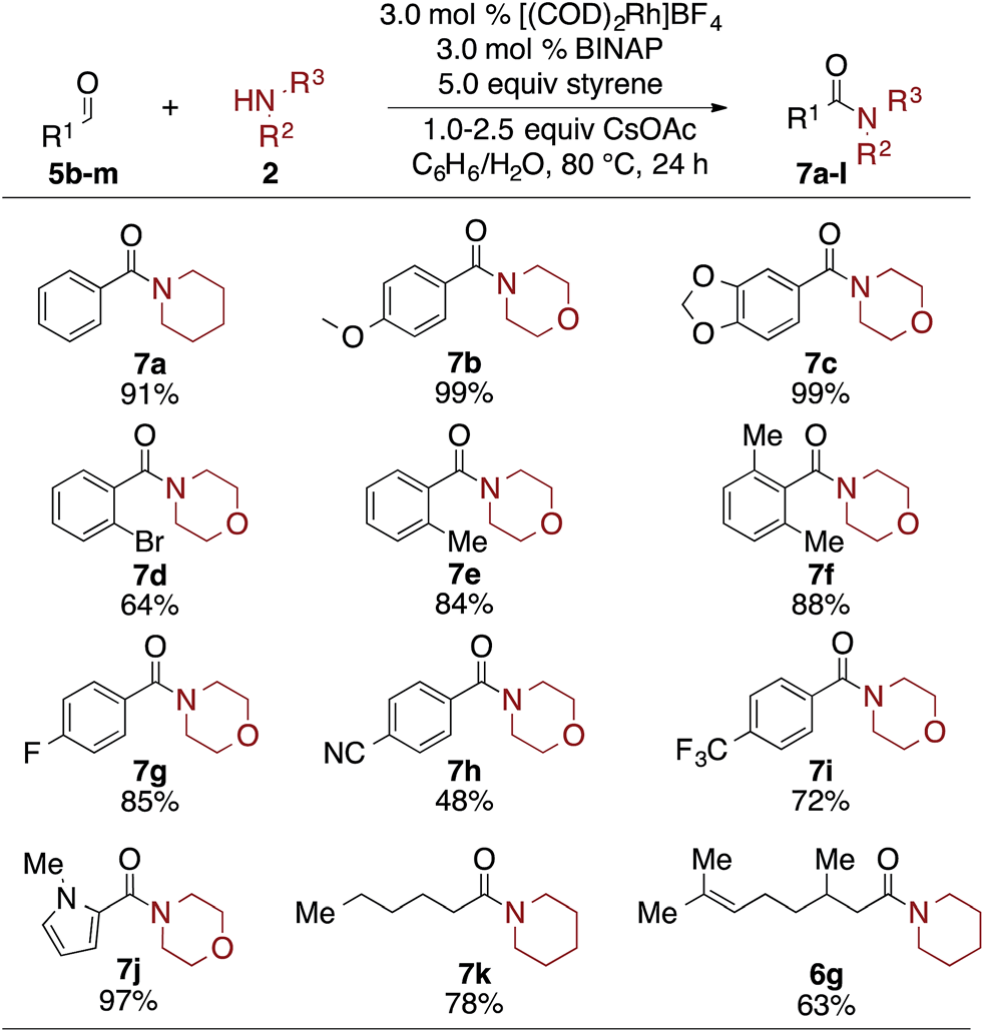

^*a*^Alcohol or aldehyde (0.25 mmol, 1.0 equiv.), amine (3.0 equiv.), [(COD)_2_Rh]BF_4_ (3.0 mol%), BINAP (3.0 mol%), styrene (5.0 equiv.), CsOAc (1.5–2.5 equiv.), benzene (0.2 mL, 1.2 M), DI H_2_O (0.2 mL).^12^

### Competition experiments

**Scheme 2:**
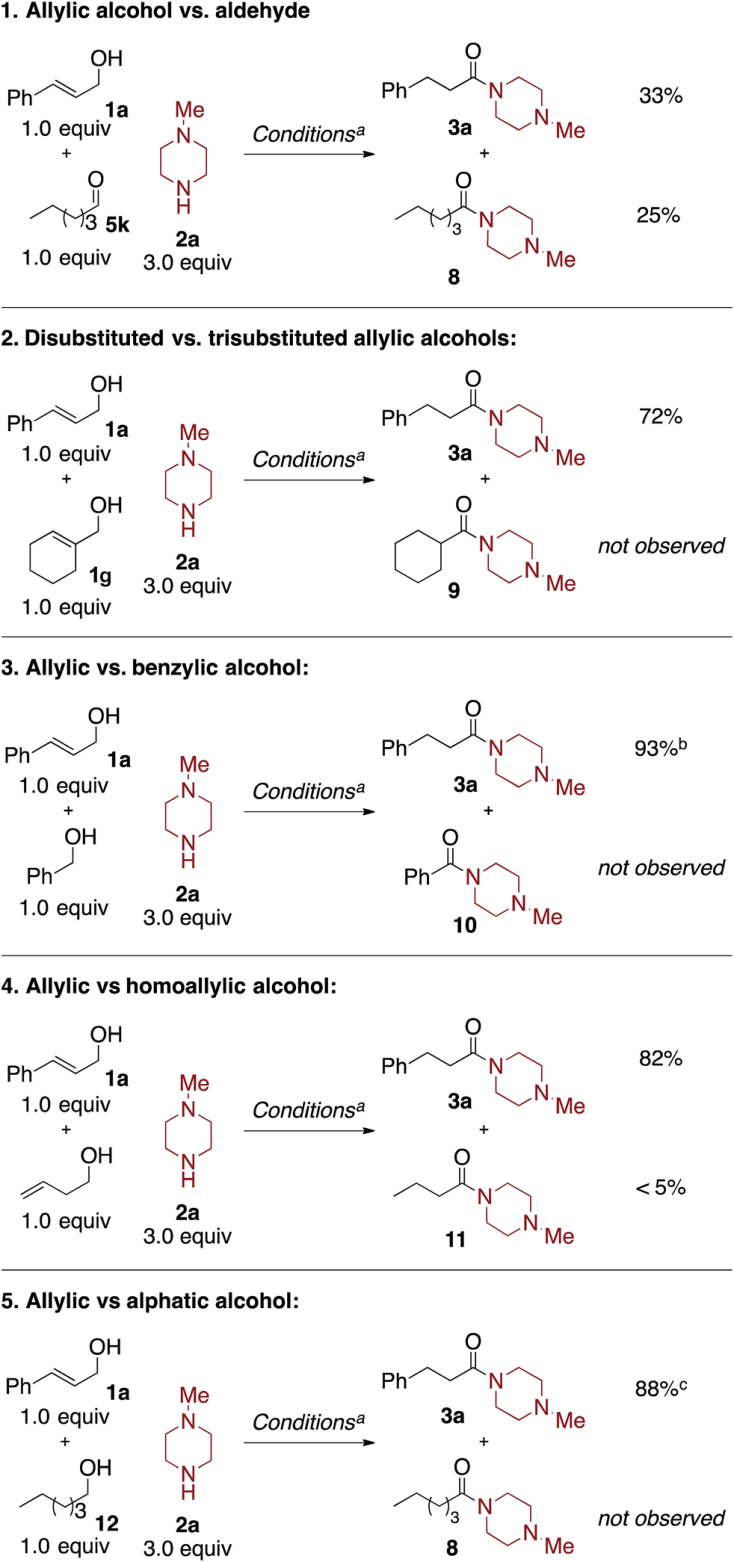
Competition experiments. ^a^ Standard conditions are: alcohol/aldehyde 1 (1.0 equiv.), alcohol/aldehyde 2 (1.0 equiv.), **2a** (3.0 equiv.), [(COD)_2_Rh]BF_4_ (3.0 mol%), BINAP (3.0 mol%), styrene (3.0 equiv.), CsOAc (2.5 equiv.), benzene (0.2 mL, 1.2 M), DI H_2_O (0.2 mL), 80 °C, 4 hours. ^b^ Benzyl 3-phenylpropanoate was formed in 7% yield. ^c^ Hexyl 3-phenyl-propanoate was formed in 8% yield.

The synthetic utility of this oxidative amidation reaction would be significantly increased if the reaction proved to be chemoselective for allylic alcohols and aldehydes over other oxidizable functionalities, *i.e.*, simple primary alcohols. To explore the chemoselectivity of the reaction conditions, a series of competition studies were carried out (Scheme 2).

In competition experiment 1, cinnamyl alcohol (**1a**) competes against hexanal (**5k**) to compare the relative rates of the two coupling partners. Unsurprisingly, given the rapid rate of 1,3-hydride shift,10*c* the reaction is unselective, affording a 33% yield of **3a** and a 25% yield of **8** after four hours. This lack of selectivity supports the rapid Rh-catalyzed isomerization of the cinnamyl alcohol to the corresponding aldehyde; the oxidative amidation of the two aliphatic aldehydes then occurs at similar rates.

Next, in competition experiment 2, the comparative reactivity of two different allylic alcohols was explored. Under the standard reaction conditions, cinnamyl alcohol (**1a**) reacts selectively over cyclohex-1-en-1-ylmethanol (**1g**). This excellent chemoselectivity is consistent with the known rates of the Rh-catalyzed isomerization of allylic alcohols.10*c* Importantly, **1g** was observed, unisomerized, at the end of the reaction.

Competition experiments 3–5 investigate the chemo-selectivity of the oxidative amidation reaction with respect to benzylic alcohols, homoallylic alcohols, and aliphatic alcohols. When equimolar amounts of cinnamyl alcohol (**1a**) and benzyl alcohol were treated with 3.0 equivalents of **2a** under the standard reaction conditions, product **3a** was formed in 93% yield while **10** was not observed. This indicates that our conditions are highly selective for allylic alcohols over easily oxidizable benzylic alcohols.4h,m Moreover, in competition experiment 4, less than 5% amide product **11** was observed when cinnamyl alcohol and 3-buten-1-ol are subjected to the reaction conditions, which afforded an 82% yield of **3a**. Notably, 3-buten-1-ol affords <5% yield of **11** under the standard reaction conditions, in the absence of cinnamyl alcohol.^12^ Finally, competition experiment 5 demonstrates that cinnamyl alcohol (**1a**) reacts selectively over hexan-1-ol (**12**), to afford an 88% yield of **3a**; **8** was not observed. These experiments exhibit the excellent chemoselectivity of this Rh-catalyzed oxidative amidation reaction for coupling allylic alcohols and aldehydes selectively.

### Mechanistic investigations

Next, the requirement of excess amine and super-stoichiometric base was investigated. When a equimolar amounts of amine and allyl alcohol/aldehyde are added to the reaction the desired amide is formed in only 20% yield along with 13% enamine along with both unreacted aldehyde and aldol condensation products (Table S6,† entry 1). However, in the presence of excess amine excellent yields of the amide are observed. It is proposed that the excess amine forces the equilibrium, between aldehyde and enamine/imine, to the enamine/imine thereby decreasing the concentration of aldehyde and preventing the undesired aldol condensation from occurring. The base additive, CsOAc or KOH, could serve two possible roles, either acting as a proton shuttle or as a ligand on the catalyst. The pH of the water layer in the oxidative amidation reactions is 9.2; when the CsOAc solution is replaced with various buffers (pH = 5.2, 7.5, or 9.0) the reaction are equally efficient as to those in the absence of base (Table S9†). This suggests that the base is acting as a ligand to the catalyst and that the requirement of excess base is due to the solubility differential of the anion in water *versus* benzene.

**Scheme 3:**
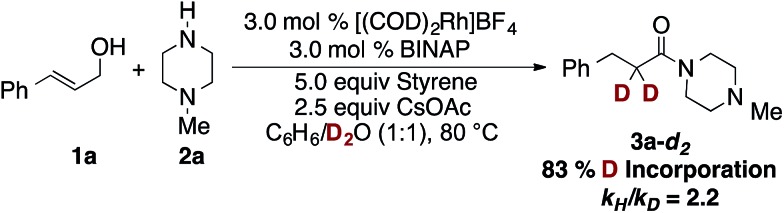
Deuterium studies.

To gain further mechanistic insight into this reaction, we conducted an isotope incorporation experiment, replacing H_2_O with D_2_O. After the reaction had gone to completion, 83% deuterium was incorporated at the α-position of the product (Scheme 3). Importantly, in the absence of catalyst and *N*-methylpiperazine, no deuterium incorporation is observed into amide **3a** or 3-phenylpropanal. This suggests that water reacts with the enamine/imine to reform the reactive hemiaminal,^13^ which is different from many Ru-catalyzed dehydrogenative coupling of alcohols with amines, where *in situ* formed aldehydes stay bounded to metal center to avoid the imine formation.4c–f,i–k Comparison of the initial rate of the reaction in H_2_O and D_2_O revealed a *k*_H_/*k*_D_ = 2.2, indicating the enamine–hemiaminal equilibrium occurs at or before the turnover-limiting step. Alternatively, the primary *k.i.e.* could be attributed to cleavage of N–H(D) bond in hemiaminal formation, due to the exchange of amine proton with D_2_O.

**Scheme 4:**
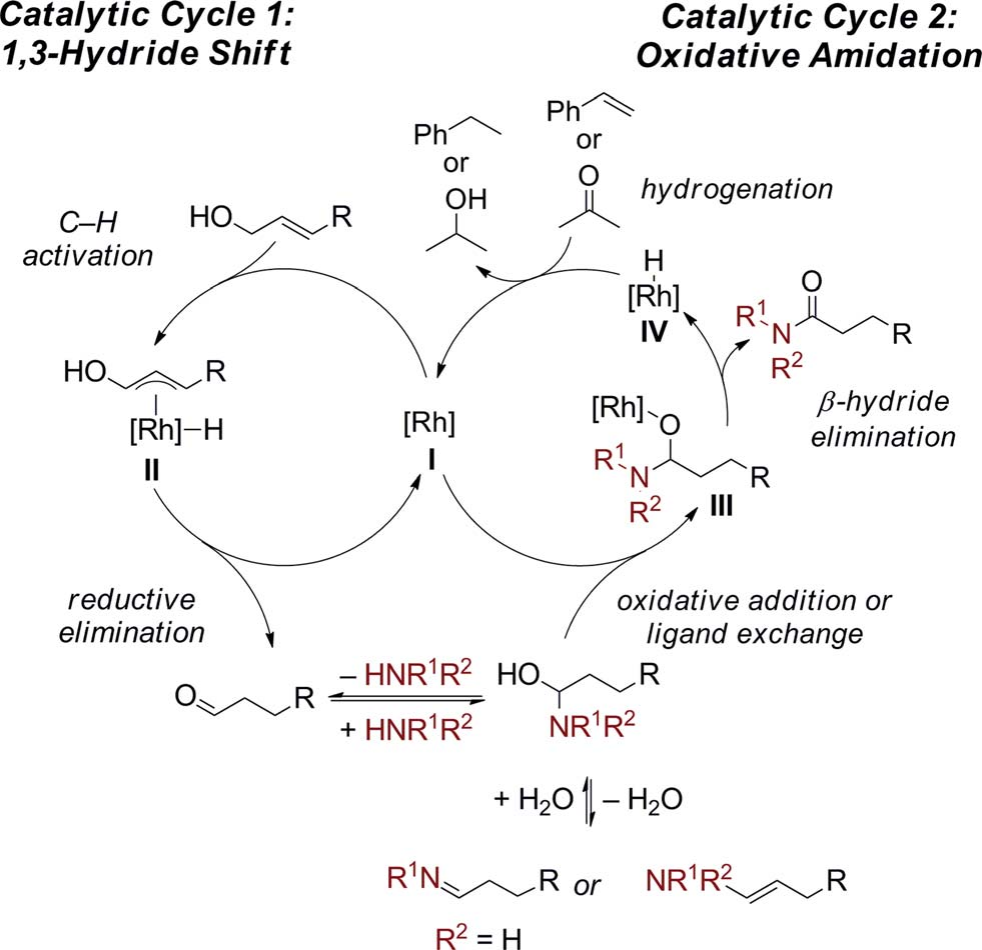
Proposed dual catalytic cycle.

The proposed dual catalytic cycles for this oxidative amidation reaction are shown in Scheme 4. First, a Rh-mediated 1,3-hydride shift^10^ occurs to form an aldehyde from the allylic alcohol. The aldehyde then condenses with the amine to generate the enamine/imine, which is in equilibrium with the hemiaminal. The oxidation of the hemiaminal could occur through either a Rh(i)/Rh(iii) or a redox neutral Rh(i) catalytic cycle. In the first, oxidative addition of the hemiaminal OH into the Rh(i) generates a Rh(iii) (H)OCHNR_2_ (**III**). Subsequent β-hydride elimination generates the amide and a Rh(iii)–(H)_2_ (**IV**) complex which is reduced to Rh(i) through hydrogenation of the styrene or acetone. Alternatively, in a redox neutral catalytic cycle, ligand exchange of the [Rh] (**I**) with the hemiaminal affords the Rh–alkoxide (**III**) then undergoes β-hydride elimination to generate the amide and the Rh–H (**IV**). Insertion of the hydride into styrene/acetone followed by protolytic cleavage or ligand exchange affords the ethyl benzene/isopropanol, respectively, and either [Rh] complex **I** or **III**. Currently, we can not distinguish between the two possible mechanistic pathways and further mechanistic investigations are ongoing.^14^

## Conclusions

In conclusion, conditions have been developed for the chemoselective oxidative amidation of allylic alcohols or aldehydes, using styrene or acetone as hydrogen acceptors. This methodology presents a general protocol for the synthesis of amides, which is effective for both primary and secondary alkyl/aryl amines. Current efforts are focusing on expanding the scope of nucleophiles and developing asymmetric conditions^15^ for the transformation.

## Supplementary Material

Supplementary information
