# Supplementary material for: Rhodium-catalyzed oxidative amidation of allylic alcohols and aldehydes: effective conversion of amines and anilines into amides
Source: Chem Sci. 2015 Oct 27;7(2):969–75. doi: 10.1039/c5sc03103f (PMC5954618; doi:10.1039/c5sc03103f)
Supplement: Supplementary file 1 [file SC-007-C5SC03103F-s001.pdf]

Electronic Supplementary Material (ESI) for Chemical Science.
This journal is © The Royal Society of Chemistry 2015

Rhodium-catalyzed oxidative amidation of allylic alcohols and aldehydes: effective conversion of amines and anilines into amides

Zhao Wu and Kami L. Hull

Department of Chemistry, University of Illinois at Urbana-Champaign, Urbana, IL 61820

Supporting Information

Table of Contents

A. General Information **S2**

B. Select Optimization Reactions **S3**

C. Control Experiments **S11**

D. Robust Chemical Screens for the Rapid Assessment **S16**

E. Deuterium Incorporation Studies **S17**

F. Kinetic Isotope Effect Studies **S21**

G. Competition Studies **S22**

H. Kinetic Profile of E- vs Z- Allylic Alcohol Amidation **S24**

I. Experimental Procedure, Isolation, and Characterization **S25**

J. References **S62**

K. Spectral Data **S63**

A. General Information

General Experimental Procedures: All reactions were carried out in flame-dried (or oven-dried at 140 °C for at least 2 h) glassware under an atmosphere of nitrogen unless otherwise indicated. Nitrogen was dried using a drying tube equipped with Drierite™ unless otherwise noted. Air- and moisture-sensitive reagents were handled in a nitrogen-filled glovebox (working oxygen level ~ 0.1 ppm). Column chromatography was performed with silica gel from Grace Davison Discovery Sciences (35-75 μm) with a column mixed as a slurry with the eluent and was packed, rinsed, and run under air pressure. Analytical thin-layer chromatography (TLC) was performed on precoated glass silica gel plates (by EMD Chemicals Inc.) with F-254 indicator. Visualization was either by short wave (254 nm) ultraviolet light, or by staining with potassium permanganate followed by brief heating on a hot plate or by a heat gun. Distillations were performed using a 3 cm short-path column under reduced pressure or by using a Hickman still at ambient pressure.

Instrumentation: ^{1}H NMR and ^{13}C NMR were recorded on a Varian Unity 400/500 MHz (100/125 MHz respectively for ^{13}C) or a VXR-500 MHz spectrometer. Spectra were referenced using either $CDCl_3$ or C_6D_6 as solvents (unless otherwise noted) with the residual solvent peak as the internal standard (^{1}H NMR: δ 7.26 ppm, ^{13}C NMR: δ 77.00 ppm for $CDCl_3$ and ^{1}H NMR: δ 7.15 ppm, ^{13}C NMR: δ 128.60 ppm for C_6D_6). Chemical shifts were reported in parts per million and multiplicities are as indicated: s (singlet,) d (doublet,) t (triplet,) q (quartet,) p (pentet,) m (multiplet,) and br (broad). Coupling constants, *J*, are reported in Hertz and integration is provided, along with assignments, as indicated. Analysis by Gas Chromatography-Mass Spectrometry (GC-MS) was performed using a Shimadzu GC-2010 Plus Gas chromatograph fitted with a Shimadzu GCMS-QP2010 SE mass spectrometer using electron impact (EI) ionization after analytes traveled through a SHRXI–5MS- 30m x 0.25 mm x 0.25 μm column using a helium carrier gas. Data are reported in the form of m/z (intensity relative to base peak = 100). Gas Chromatography (GC) was performed on a Shimadzu GC-2010 Plus gas chromatograph with SHRXI–MS- 15m x 0.25 mm x 0.25 μm column with nitrogen carrier gas and a flame ionization detector (FID). Low-resolution Mass Spectrometry and High Resolution Mass Spectrometry were performed in the Department of Chemistry at University of Illinois at Urbana-Champaign. The glove box, MBraun LABmaster sp, was maintained under nitrogen atmosphere. Melting points were recorded on a Thomas Hoover capillary melting point apparatus and are uncorrected.

Materials: Solvents used for extraction and column chromatography were reagent grade and used as received. Reaction solvents tetrahydrofuran (Fisher, unstabilized HPLC ACS grade), diethyl ether (Fisher, BHT stabilized ACS grade), methylene chloride (Fisher, unstabilized HPLC grade), dimethoxyethane (Fisher, certified ACS), toluene (Fisher, optima ACS grade), 1,4-dioxane (Fisher, certified ACS), acetonitrile (Fisher, HPLC grade), and hexanes (Fisher, ACS HPLC grade) were dried on a Pure Process Technology Glass Contour Solvent Purification System using activated Stainless Steel columns while following manufacture's recommendations for solvent preparation and dispensation unless otherwise noted. All amines were distilled and degassed by the freeze-pump-thaw method, and were stored under an atmosphere of nitrogen in glove box before use. All liquid aldehydes were distilled prior to use, and ketones, benzophenone and cyclohexanone, were used as received.

B. Select Optimization Reactions

Table S1. Varying the ligand in optimizing the Rh-catalyzed oxidative amidation reaction [a]

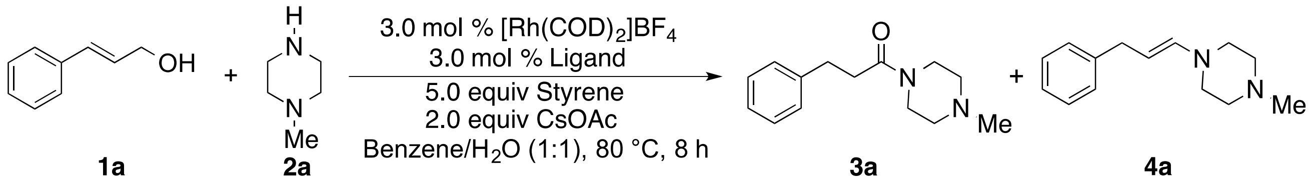

Entry	Ligand	% Yield **3a**[b]	% Yield **4a**[b]
1	dppp	72	22
2	dppb	57	32
3	dpppent	9	70
4	dppf	31	64
5	bipyridine	4	2
6	DPEphos	11	75
7	Xantphos	17	68
8	Davephos	18	34
9	BINAP	87	2
10	None	9	10

[a] Unless otherwise specified, all reactions were set up in oven-dried 4mL vials and performed with 3.0 mol % catalyst at 1.25 M in **alcohol** (0.25 mmol) with 3.0 equiv of **amine** for 8 h at 80 °C. **Amine** was distilled prior to use. [b] *In situ* yields were determined by GC analysis of the crude reaction mixture and comparison to diphenylmethane (20 μL, 0.12 mmol, 0.48 equiv.) as an internal standard.

Table S2. Varying the solvent in optimizing the Rh-catalyzed oxidative amidation reaction [a]

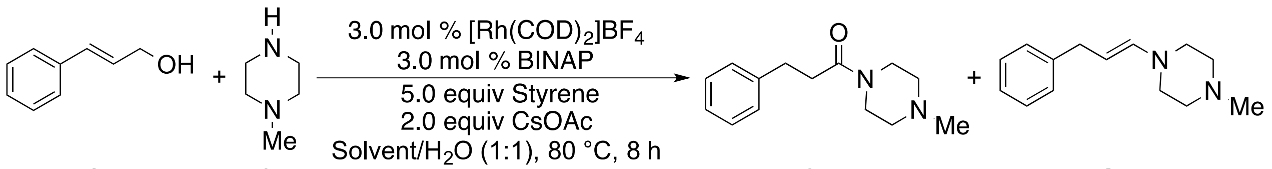

1a **2a** **3a** **4a**

Entry	Solvent	% Yield **3a**[b]	% Yield **4a**[b]
1	Benzene	91	3
2	Toluene	79	7
3	Hexanes	88	2
4	DME	80	0
5	Et_2O	83	0
6	1,4-dioxane	75	0
7	THF	82	0
8[c]	MeCN	43	0

[a] Unless otherwise specified, all reactions were set up in oven-dried 4mL vials and performed with 3.0 mol % catalyst at 1.25 M in **alcohol** (0.25 mmol) with 3.0 equiv of **amine** for 8 h at 80 °C. **Amine** was distilled prior to use. [b] *In situ* yields were determined by GC analysis of the crude reaction mixture and comparison to diphenylmethane (20 µL, 0.12 mmol, 0.48 equiv.) as an internal standard. [c] Aldol condensation product was observed.

Table S3. Varying the concentration, and benzene/H_2O ratio in optimizing the Rh-catalyzed oxidative amidation reaction [a]

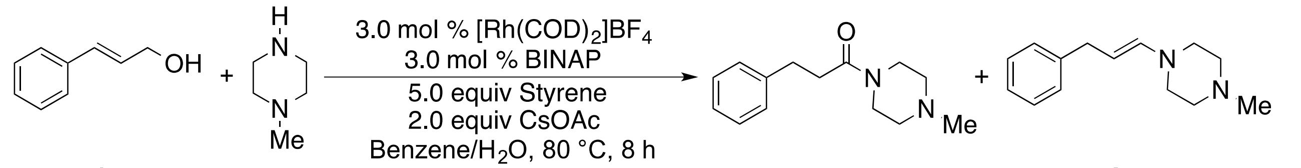

1a **2a** **3a** **4a**

Entry	Concentration (M)	Benzene: H_2O	% Yield 3a[b]	% Yield 4a[b]
1	0.31	1 : 1	35	14
2	0.62	1 : 1	60	5
3	1.2	1 : 1	89	0
4	2.5	1 : 1	88	7
5	1.2	2 : 1	78	15
6	1.2	3: 1	66	34
7	1.2	4 : 1	68	32
8	1.2	10: 1	70	30
9	1.2	1 : o	49	51
10	1.2	1 : 2	62	0
11	1.2	1 : 3	34	13

[a] Unless otherwise specified, all reactions were set up in oven-dried 4mL vials and performed with 3.0 mol % catalyst at 1.25 M in **alcohol** (0.25 mmol) with 3.0 equiv of **amine** for 8 h at 80 °C. **Amine** was distilled prior to use. [b] *In situ* yields were determined by GC analysis of the crude reaction mixture and comparison to diphenylmethane (20 µL, 0.12 mmol, 0.48 equiv.) as an internal standard.

Table S4. Varying the oxidants and bases in optimizing the Rh-catalyzed oxidative amidation reaction [a]

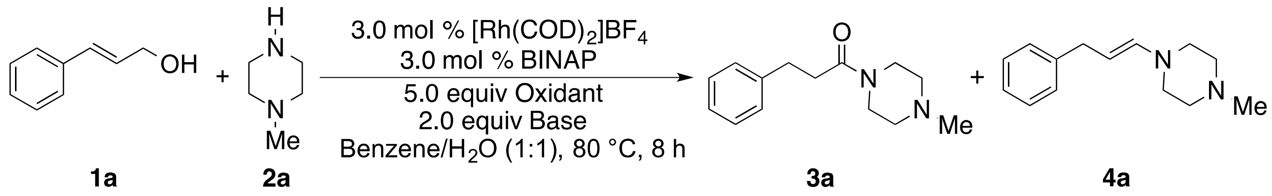

Entry	Base	Oxidant	% Yield 3a[b]	% Yield 4a[b]
1	Na_2CO_3	styrene	36	49
2	K_2CO_3	styrene	47	31
3	Cs_2CO_3	styrene	31	58
4	NaOAc	styrene	89	0
5	KOAc	styrene	88	0
6	CsOAc	styrene	89	0
7	NaO*t*Bu	styrene	17	47
8	KO*t*Bu	styrene	13	27
9	Et_3N	styrene	18	38
10	Py	styrene	63	0
11	none	styrene	39	15
12	CsOAc	norbornene	56	0
13	CsOAc	cyclohexene	39	21
14	CsOAc	tert-butylethylene	28	31
15	CsOAc	methyl-methacrylate	25	2
16	CsOAc	NMO	65	0
17	CsOAc	trans-stilbene	58	7
18	CsOAc	acetone	58	0
19	CsOAc	cyclohexanone	65	0
20	CsOAc	none	41	5

[a] Unless otherwise specified, all reactions were set up in oven-dried 4mL vials and performed with 3.0 mol % catalyst at 1.25 M in **alcohol** (0.25 mmol) with 3.0 equiv of **amine** for 8 h at 80 °C. **Amine** was distilled prior to use. [b] *In situ* yields were determined by GC analysis of the crude reaction mixture and comparison to diphenylmethane (20 µL, 0.12 mmol, 0.48 equiv.) as an internal standard.

Table S5. Varying the equivalences of oxidants and bases in optimizing the Rh-catalyzed oxidative amidation reaction [a]

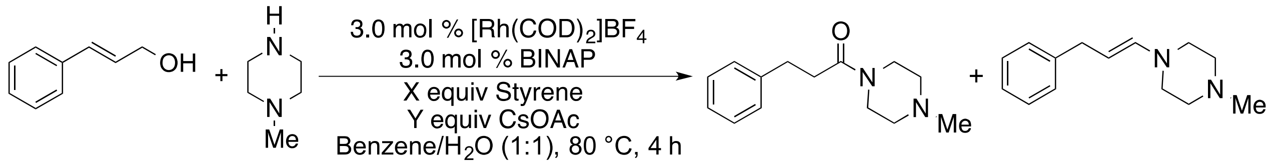

1a 2a 3a 4a

Entry	X equiv styrene	Y equiv CsOAc	% Yield 3a[b]	% Yield 4a[b]
1	5.0	0.20	48	0
2	5.0	0.50	61	0
3	5.0	1.0	63	0
4	5.0	1.5	76	0
5	5.0	2.0	81	0
6	5.0	2.5	87	0
7	5.0	3.0	85	0
8	1.0	2.5	61	0
9	2.0	2.5	73	0
10	3.0	2.5	78	0
11	4.0	2.5	80	0
12	8.0	2.5	83	0

[a] Unless otherwise specified, all reactions were set up in oven-dried 4mL vials and performed with 3.0 mol % catalyst at 1.25 M in **alcohol** (0.25 mmol) with 3.0 equiv of **amine** for 4 h at 80 °C. **Amine** was distilled prior to use. [b] *In situ* yields were determined by GC analysis of the crude reaction mixture and comparison to diphenylmethane (20 µL, 0.12 mmol, 0.48 equiv.) as an internal standard.

Table S6. Varying the equivalence of catalyst/ligand, allylic alcohol, and amine in optimizing the Rh-catalyzed oxidative amidation reaction [a]

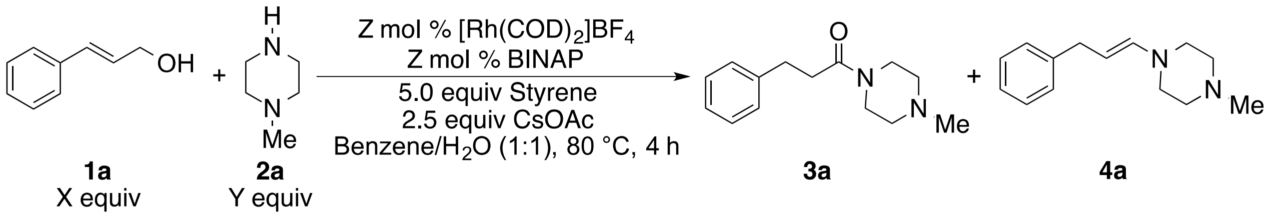

Entry	X equiv alcohol	Y equiv amine	Z mol% catalyst	% Yield 3a[b]	% Yield 4a[b]
1	1.0	1.0	3.0	20	13
2	1.0	2.0	3.0	63	0
3	1.0	3.0	3.0	87	0
4	1.0	4.0	3.0	87	0
5	1.0	5.0	3.0	85	0
6	2.0	1.0	3.0	16	35
7	3.0	1.0	3.0	15	45
8	1.0	3.0	1.0	14	65
9	1.0	3.0	2.0	62	11
10	1.0	3.0	4.0	87	0
11	1.0	3.0	5.0	90	0
12	1.0	3.0	3.0	91	0

[a] Unless otherwise specified, all reactions were set up in oven-dried 4mL vials and performed with 3.0 mol % catalyst at 1.25 M in **alcohol** (0.25 mmol) with 3.0 equiv of **amine** for 4 h at 80 °C. **Amine** was distilled prior to use. [b] *In situ* yields were determined by GC analysis of the crude reaction mixture and comparison to diphenylmethane (20 µL, 0.12 mmol, 0.48 equiv.) as an internal standard.

Table S7. Varying the temperature and reaction time in optimizing the Rh-catalyzed oxidative amidation reaction [a]

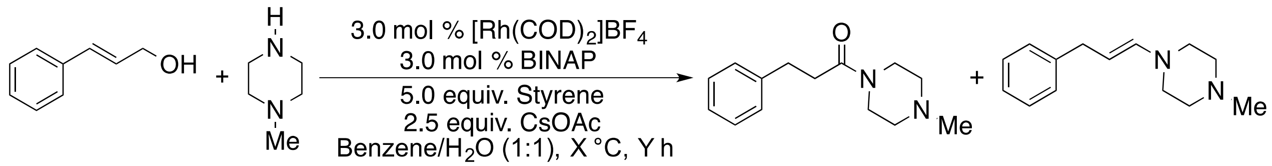

Entry	X (°C)	Y (h)	% Yield 3a[b]	% Yield 4a[b]
1	rt	8	9	46
2	40	8	10	36
3	60	8	76	16
4	80	8	91	0
5	100	8	91	0
6	120	8	86	0
7	80	0.5	43	50
8	80	1	68	15
9	80	2	75	10
10	80	4	83	1
11	80	6	83	1
12	80	8	84	0

[a] Unless otherwise specified, all reactions were set up in oven-dried 4mL vials and performed with 3.0 mol % catalyst at 1.25 M in **alcohol** (0.25 mmol) with 3.0 equiv of **amine**. **Amine** was distilled prior to use. [b] *In situ* yields were determined by GC analysis of the crude reaction mixture and comparison to diphenylmethane (20 µL, 0.12 mmol, 0.48 equiv.) as an internal standard.

Table S8. Optimization of Primary Amine Nucleophiles [a]

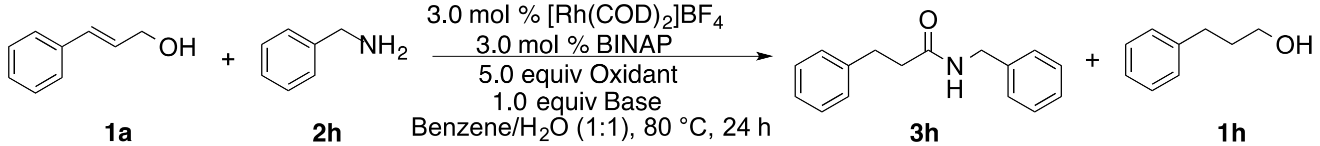

Entry	Base	Oxidant	% Yield 3h[b]	% Yield 1h[b]
1	CsOAc	styrene	4	0
2	Cs_2CO_3	styrene	35	20
3	KOH	styrene	34	26
4	$CsOH{\cdot}H_2O$	styrene	37	29
5	KOH	norbornene	56	26
6	KOH	NMO	48	37
7	KOH	acetone	84	4
8	KOH	acetone(3 equiv)	82	6
9	LiOH	acetone(3 equiv)	64	5
10	NaOH	acetone(3 equiv)	74	6
11	$CsOH{\cdot}H_2O$	acetone(3 equiv)	77	6

[a] Unless otherwise specified, all reactions were set up in oven-dried 4mL vials and performed with 3.0 mol % catalyst at 1.25 M in **alcohol** (0.25 mmol) with 3.0 equiv of **amine** for 24 h at 80 °C. **Amine** was distilled prior to use. [b] *In situ* yields were determined by GC analysis of the crude reaction mixture and comparison to diphenylmethane (20 µL, 0.12 mmol, 0.48 equiv.) as an internal standard.

C. Control Reaction

1) **Table S9.** Control reactions for base and water in oxidative amidation of allylic alcohol[a]

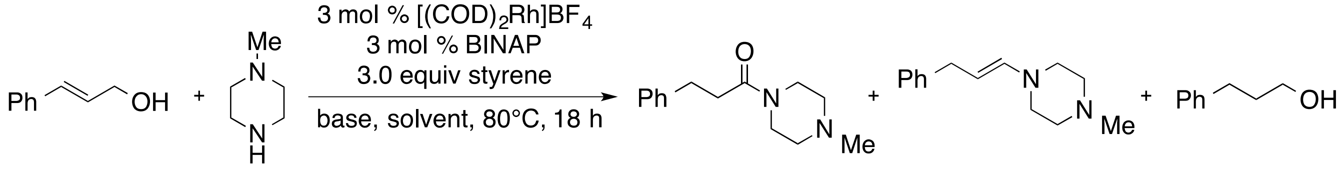

1a **2a** **3a** **4a** **1h**

Entry	Solvent	Solvent Ratio	Base	% Yield 3a[b]	% Yield 4a[b]	% Yield 1h[b]
1	C_6H_6/H_2O	1:1	CsOAc	88	0	< 5
2	C_6H_6	-	none	1	44	< 5
3	C_6H_6/H_2O	1:1	none	59	0	< 5
4	C_6H_6/H_2O	1:2	none	68	0	< 5
5	C_6H_6	-	CsOAc	60	38	< 5
6	C_6H_6	1:1	buffer 1[c]	55	5	< 5
7	C_6H_6	1:2	buffer 1[c]	68	8	< 5
8	C_6H_6	1:1	buffer 2[d]	65	4	< 5
9	C_6H_6	1:2	buffer 2[d]	69	10	< 5
10	C_6H_6	1:1	buffer 3[e]	63	5	< 5
11	C_6H_6	1:2	buffer 3[e]	70	4	< 5

[a] Standard conditions are: **1a** (1.0 equiv) **3a** (3.0 equiv), $[(COD)_2Rh]BF_4$ (3.0 mol%), BINAP (3.0 mol%), styrene (3.0 equiv), CsOAc (1.5 equiv), benzene (0.2 mL, 1.2 M), 80°C, 4 hours. [b] *In situ* yields were determined by GC analysis of the crude reaction mixture and comparison to diphenylmethane (20 μL, 0.12 mmol, 0.48 equiv.) as an internal standard.
[c] buffer 1: pH=9.0, 20 nM Tris·HCl, 50 nM KCl, HCl [d] buffer 2: pH=7.5, 20 nM Tris·HCl, 50 nM KCl, HCl [e] buffer 3: pH=5.2, 300mM NaOAc, HOAc.

2) Oxidative amidation of homoallylic alcohol

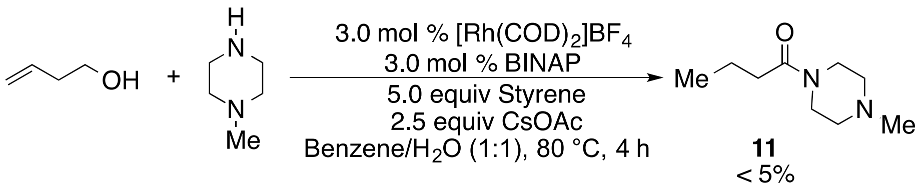

Procedure: $[Rh(COD)_2]BF_4$ (3.1 mg, 0.0075 mmol, 3.0 mol %), BINAP (4.7 mg, 0.0075 mmol, 3.0 mol %), and CsOAc (120 mg, 0.62 mmol, 2.5 equiv) were added to a 4 mL vial equipped with a stir bar. After purging with nitrogen, to the vial was added sequentially benzene (0.2 mL), 1-methylpiperazine (65 μL, 0.75 mmol, 3.0 equiv), styrene (145 μL, 1.25 mmol, 5.0 equiv), but-3-en-1-ol (21 μL, 0.25 mmol, 1.0 equiv), and D_2O (0.2 mL). The resulting solution was allowed to stir for 4 h at 80 °C. Then, the reaction vial was cooled to room temperature followed by the addition of diphenylmethane as an internal standard for analysis of the crude reaction mixture. Only trace amount of **11** was not observed by analysis of reaction mixture by gas chromatography.

GC of Reaction Crude

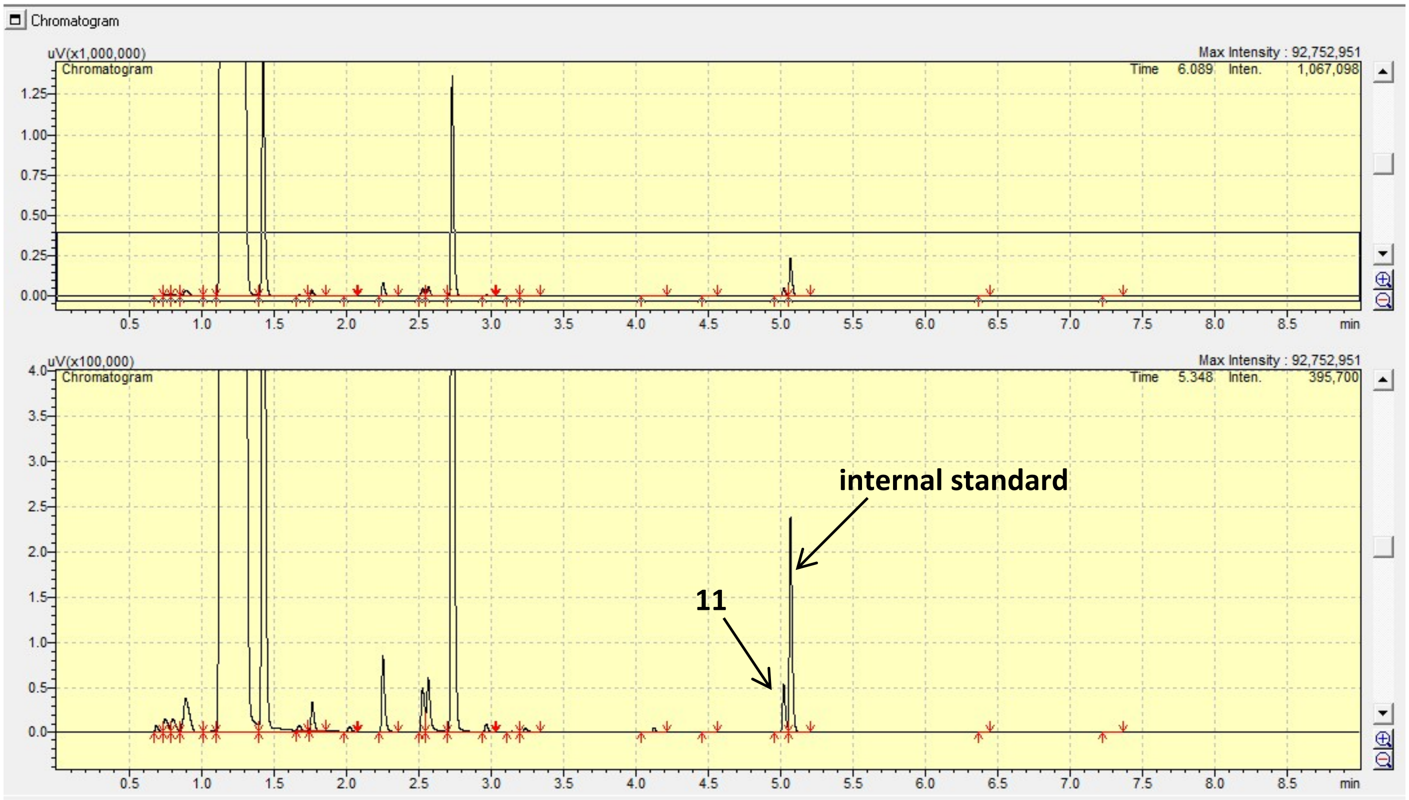

3) Oxidative amidation of primary alcohol **1h**

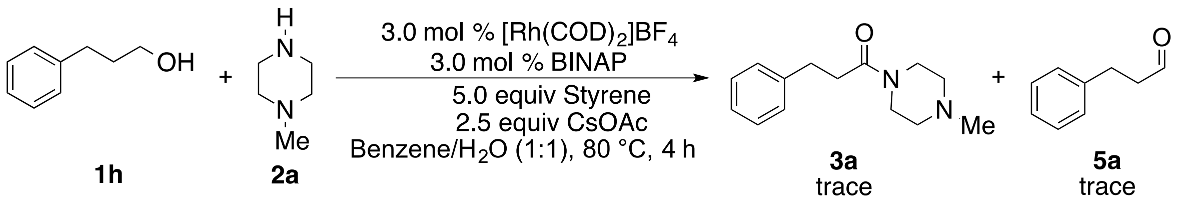

Procedure: $[Rh(COD)_2]BF_4$ (3.1 mg, 0.0075 mmol, 3.0 mol %), BINAP (4.7 mg, 0.0075 mmol, 3.0 mol %), and CsOAc (120 mg, 0.62 mmol, 2.5 equiv) were added to a 4 mL vial equipped with a stir bar. After purging with nitrogen, to the vial was added sequentially benzene (0.2 mL), 1-methylpiperazine (65 μL, 0.75 mmol, 3.0 equiv), styrene (145 μL, 1.25 mmol, 5.0 equiv), hydrocinnamyl alcohol (**1h**, 34 μL, 0.25 mmol, 1.0 equiv), and D_2O (0.2 mL). The resulting solution was allowed to stir for 4 h at 80 °C. Then, the reaction vial was cooled to room temperature followed by the addition of diphenylmethane as an internal standard for analysis of the crude reaction mixture. **3a** and **5a** were observed as trace peaks by when analyzing the reaction mixture by gas chromatography.

GC of Reaction Crude

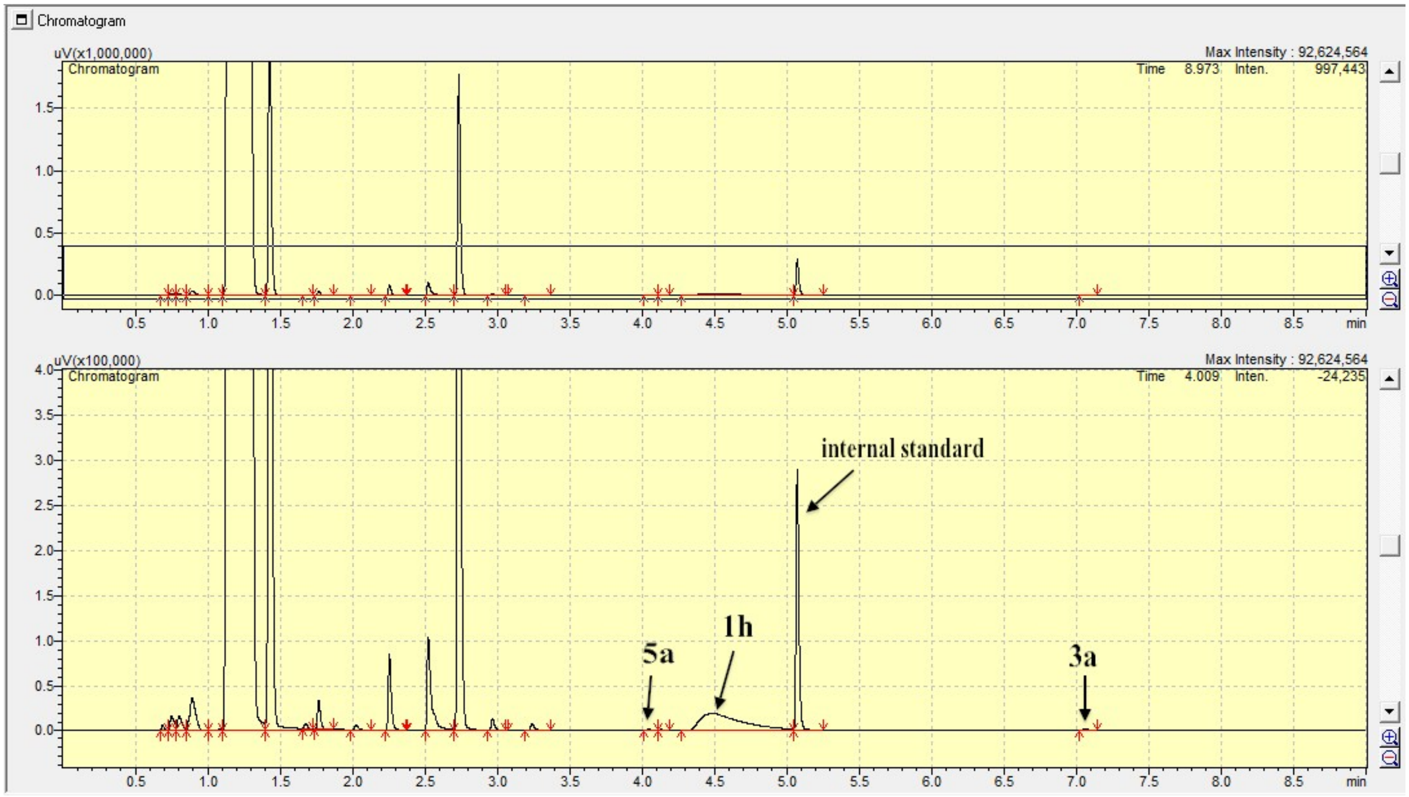

4) Oxidative amidation of carboxylic acid

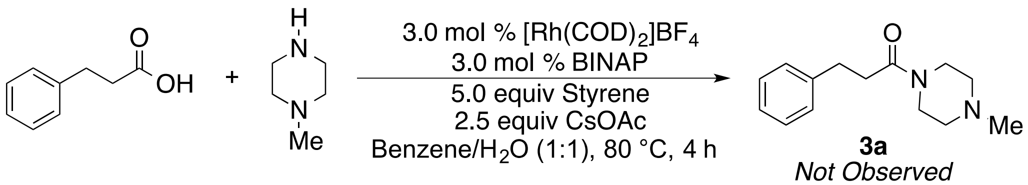

3a
Not Observed

Procedure: $[Rh(COD)_2]BF_4$ (3.1 mg, 0.0075 mmol, 3.0 mol %), BINAP (4.7 mg, 0.0075 mmol, 3.0 mol %), and CsOAc (120 mg, 0.62 mmol, 2.5 equiv) were added to a 4 mL vial equipped with a stir bar. After purging with nitrogen, to the vial was added sequentially benzene (0.2 mL), 1-methylpiperazine (65 μL, 0.75 mmol, 3.0 equiv), styrene (145 μL, 1.25 mmol, 5.0 equiv), 3-phenylpropanoic acid (38 mg, 0.25 mmol, 1.0 equiv), and D_2O (0.2 mL). The resulting solution was allowed to stir for 4 h at 80 °C. Then, the reaction vial was cooled to room temperature followed by the addition of diphenylmethane as an internal standard for analysis of the crude reaction mixture. **3a** was not observed by analysis of reaction mixture by gas chromatography.

GC of Reaction Crude

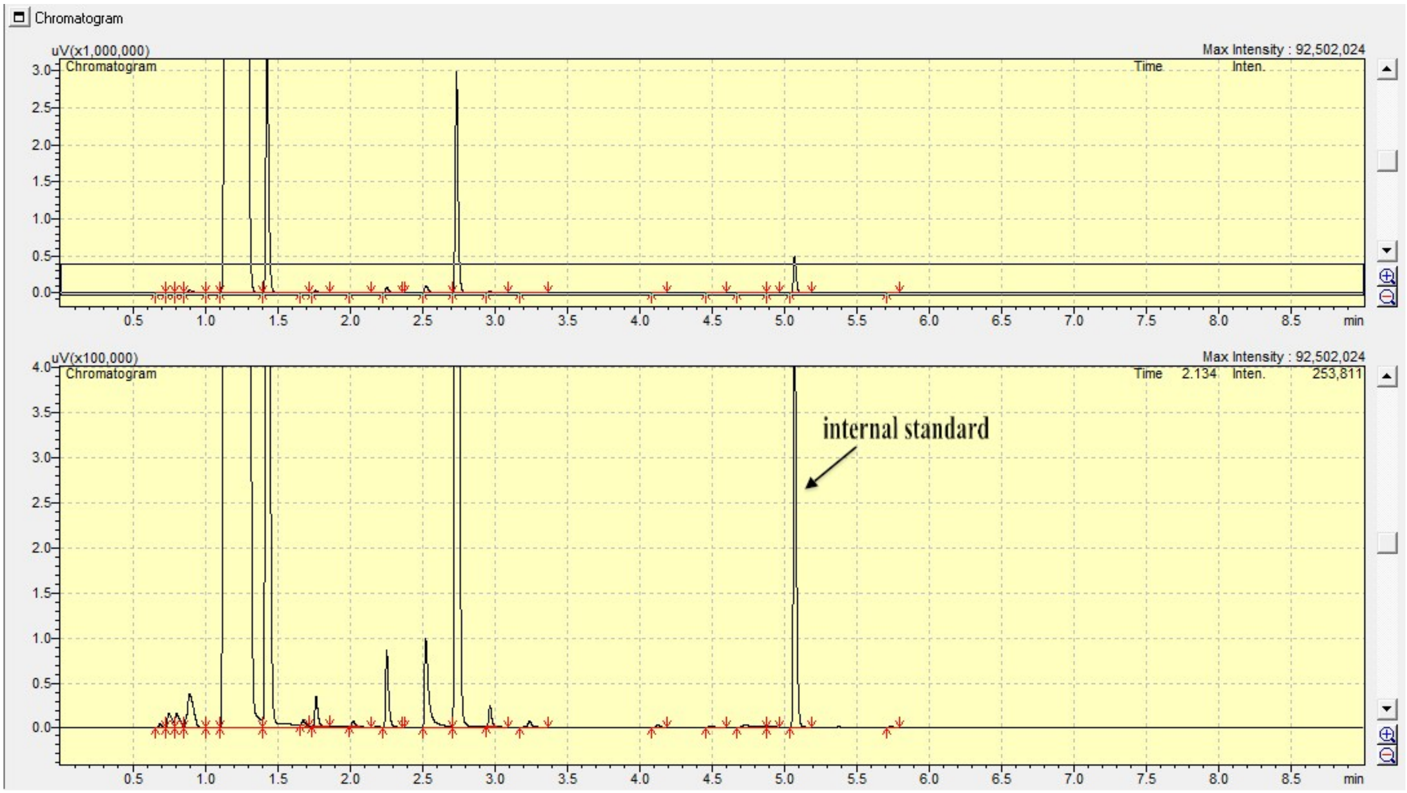

5) Oxidative amidation of carboxylic ester **12**

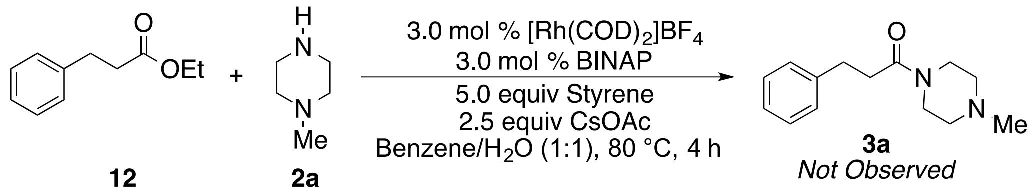

Procedure: $[Rh(COD)_2]BF_4$ (3.1 mg, 0.0075 mmol, 3.0 mol %), BINAP (4.7 mg, 0.0075 mmol, 3.0 mol %), and CsOAc (120 mg, 0.62 mmol, 2.5 equiv) were added to a 4 mL vial equipped with a stir bar. After purging with nitrogen, to the vial was added sequentially benzene (0.2 mL), 1-methylpiperazine (65 µL, 0.75 mmol, 3.0 equiv), styrene (145 µL, 1.25 mmol, 5.0 equiv), ethyl 3-phenylpropanoate (**12**, 44 µL, 0.25 mmol, 1.0 equiv), and D_2O (0.2 mL). The resulting solution was allowed to stir for 4 h at 80 °C. Then, the reaction vial was cooled to room temperature followed by the addition of diphenylmethane as an internal standard for analysis of the crude reaction mixture. **3a** was not observed by analysis of reaction mixture by gas chromatography.

GC of Reaction Crude

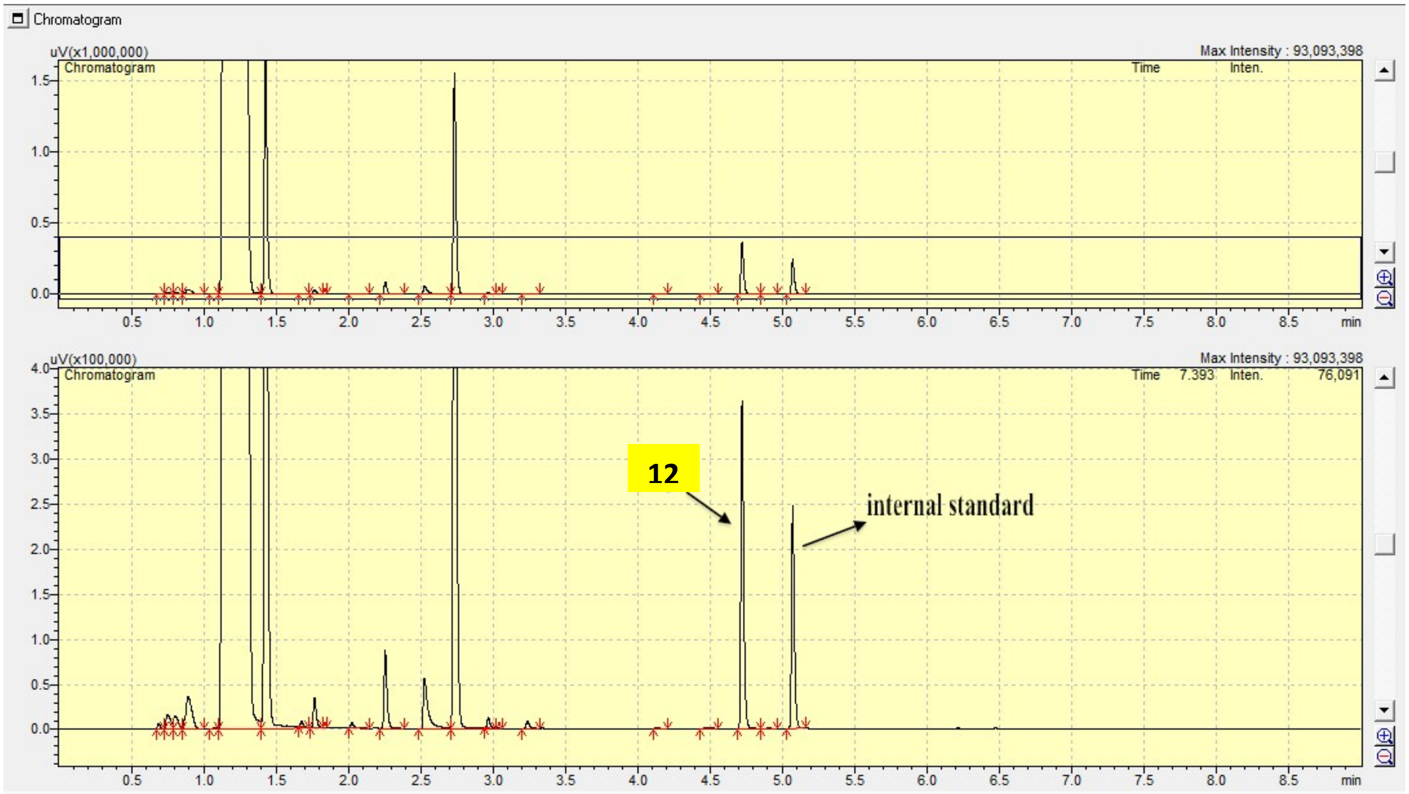

D. Robust Chemical Screens for the Rapid Assessment of Allylic alcohols Amidation

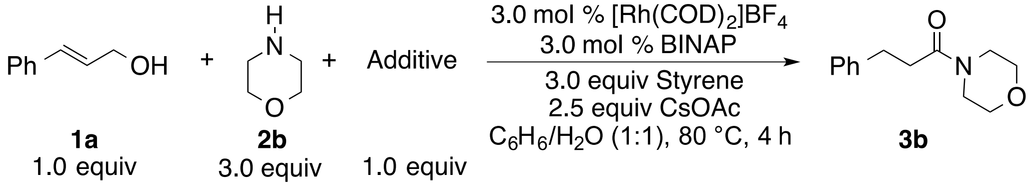

Entry	Additive	Yield of **3b** (%)	Additive remaining (%)
1	None	83	---
2	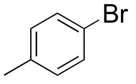	85	> 99%
3	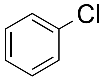	83	> 99%
4	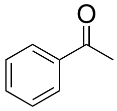	84	92%
5	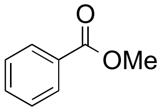	86	98%
6	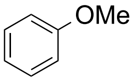	84	99%

Experiment: $[Rh(COD)_2]BF_4$ (3.1 mg, 0.0075 mmol, 3.0 mol %), BINAP (4.7 mg, 0.0075 mmol, 3.0 mol %), and CsOAc (120 mg, 0.62 mmol, 2.5 equiv) were added to a 4 mL vial equipped with a stir bar. After purging with with nitrogen, to the vial was added sequentially benzene (0.2 mL), morpholine (65 µL, 0.75 mmol, 3.0 equiv), styrene (87 µL, 0.75 mmol, 3.0 equiv), cinnamyl alcohol (32 µL, 0.25 mmol, 1.0 equiv), additive (0.25 mmol, 1.0 equiv) and DI water (0.2 mL). The resulting solution was allowed to stir for 4 h at 80 °C. Then, the reaction vial was cooled to room temperature followed by the addition of diphenylmethane as an internal standard for GC analysis of the crude reaction mixture to determine the yields of **3a** and remaining additives.

E. Deuterium Incorporation Study

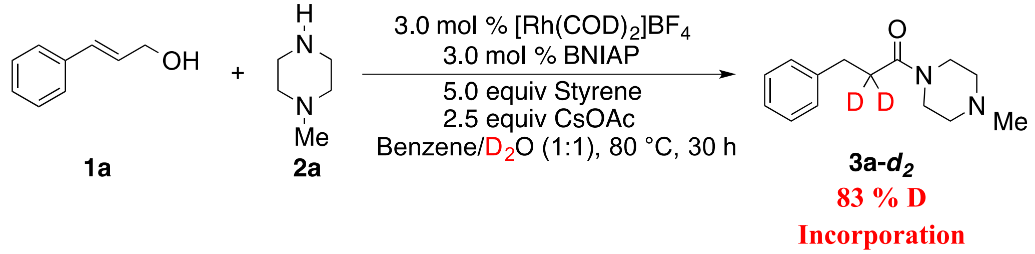

1-(4-methylpiperazin-1-yl)-3-phenylpropan-1-one-2,2-d$_2$ (3a-*d$_2$*): [Rh(COD)$_2$]BF$_4$ (3.1 mg, 0.0075 mmol, 3.0 mol %), BINAP (4.7 mg, 0.0075 mmol, 3.0 mol %), and CsOAc (120 mg, 0.62 mmol, 2.5 equiv) were added to a 4 mL vial equipped with a stir bar. After purging with nitrogen, to the vial was added sequentially benzene (0.2 mL), 1-methylpiperazine (65 µL, 0.75 mmol, 3.0 equiv), styrene (145 µL, 1.25 mmol, 5.0 equiv), cinnamyl alcohol (32 µL, 0.25 mmol, 1.0 equiv), and D_2O (0.2 mL). The resulting solution was allowed to stir for 30 h at 80 °C. Then, the reaction vial was cooled to room temperature followed by the addition of diphenylmethane as an internal standard for analysis of the crude reaction mixture. The biphasic solution was added EtOAc, extracted with 1 M HCl solution three times. The combined water layer was basified by adding 2 M NaOH solution until pH > 10, then extracted with DCM three times. The combine organic layers was dried over anhydrous $MgSO_4$, filtered and concentrated *in vacuo* to afford a yellow liquid in 69% yield.

^{1}H NMR of **3a-*d$_2$***

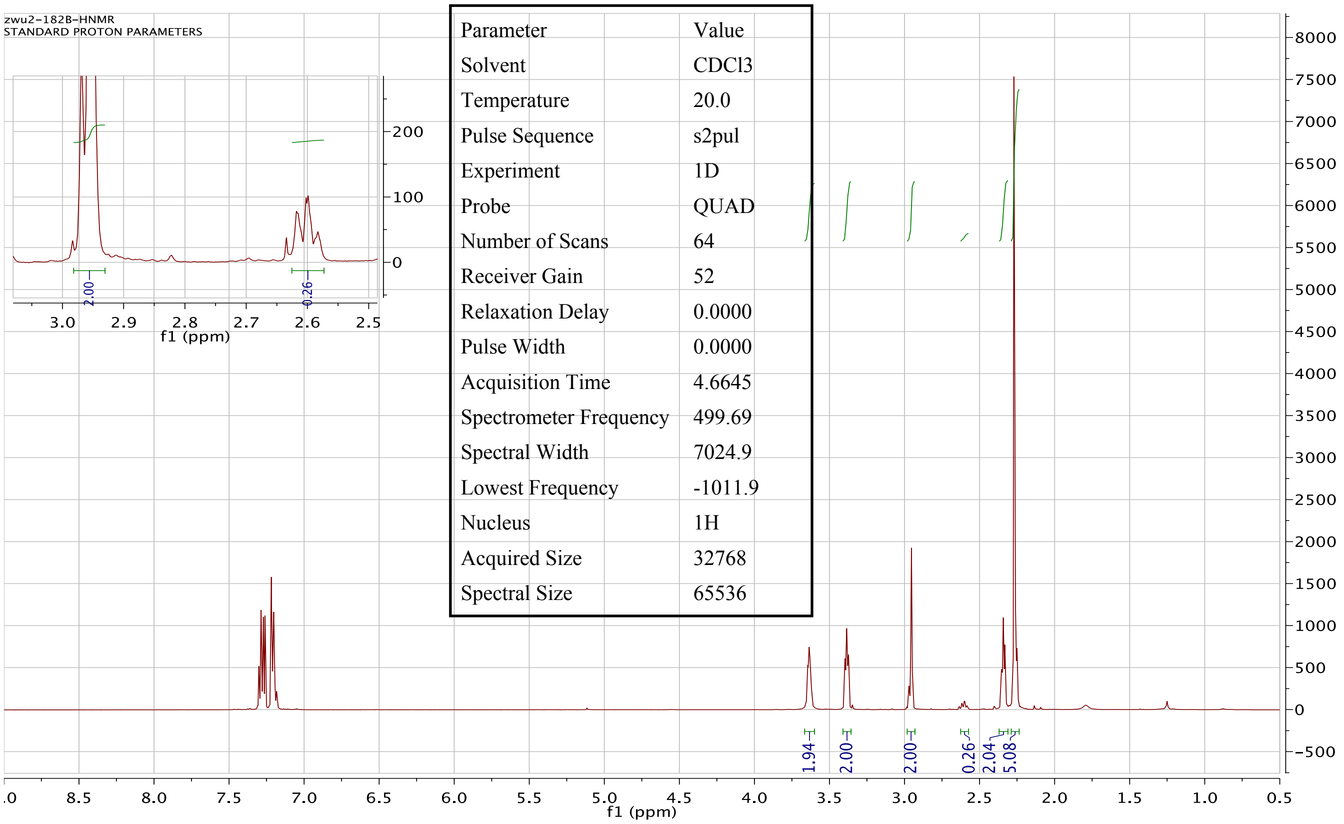

Parameter	Value
Solvent	CDCl3
Temperature	20.0
Pulse Sequence	s2pul
Experiment	1D
Probe	QUAD
Number of Scans	64
Receiver Gain	52
Relaxation Delay	0.0000
Pulse Width	0.0000
Acquisition Time	4.6645
Spectrometer Frequency	499.69
Spectral Width	7024.9
Lowest Frequency	-1011.9
Nucleus	1H
Acquired Size	32768
Spectral Size	65536

^{2}H NMR of **3a-*d*₂**

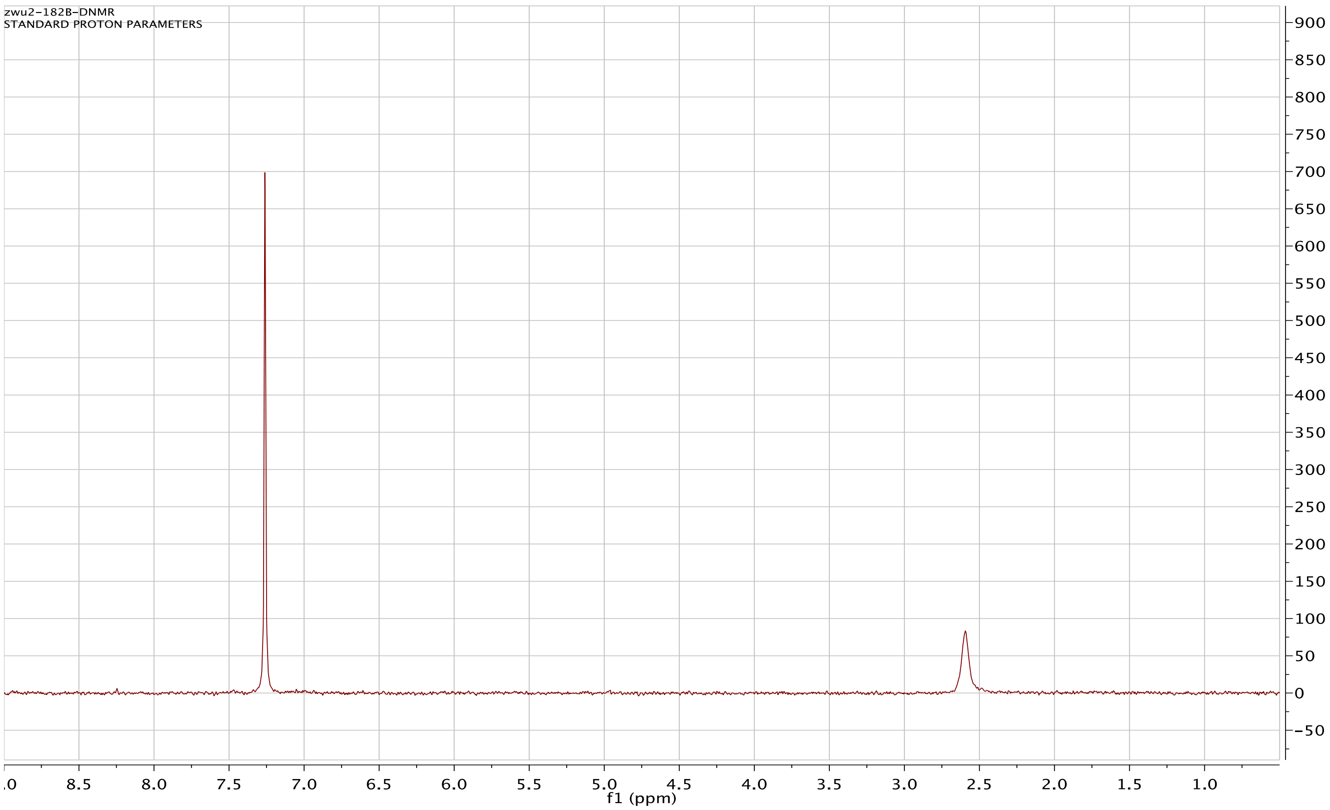

Mass spectrum of **3a-*d*₂**

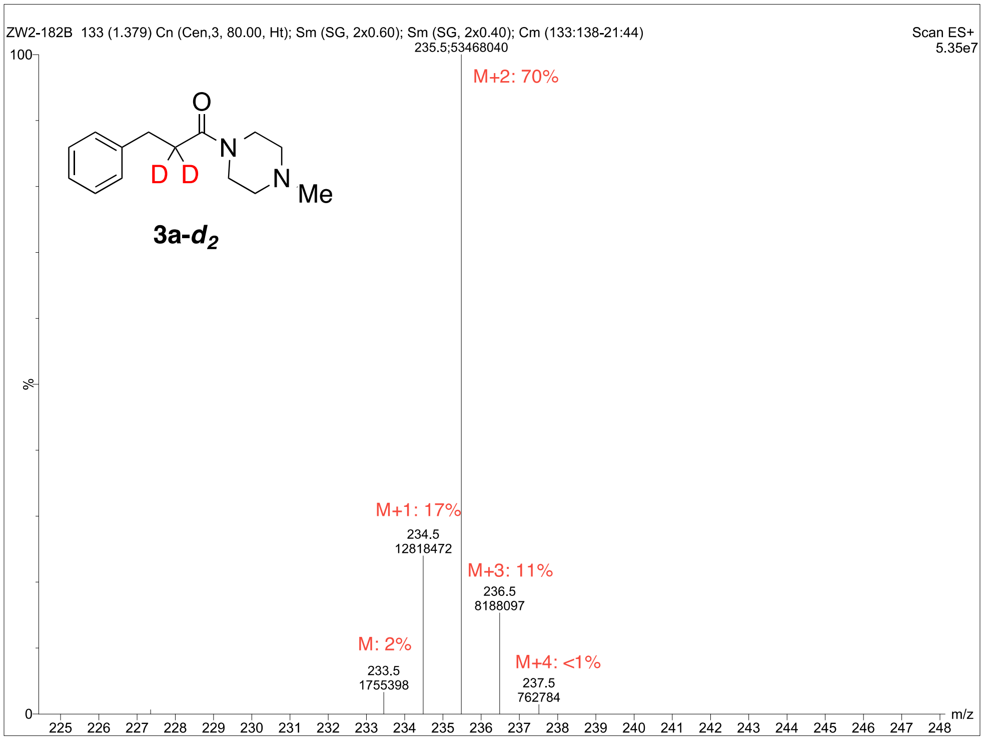

Mass spectrum of **3a**

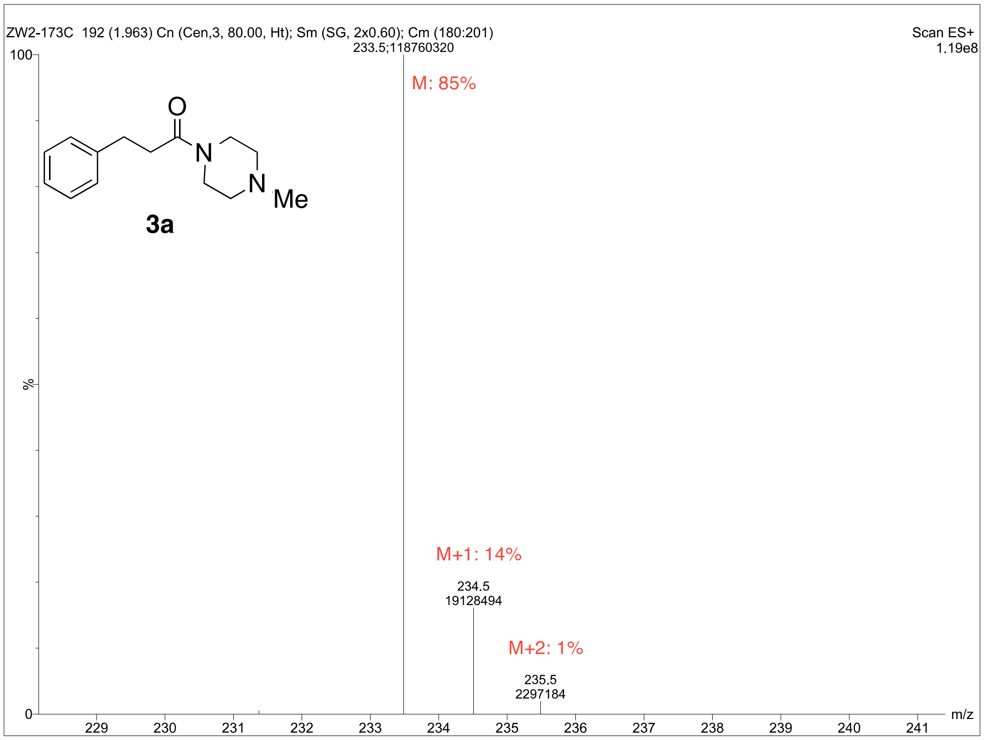

Calculation: based on mass spectrum of **3a**, M: M+1 = 85: 14. Combined with **3a-d$_2$** mass spectrum, we can get **3a-d$_2$** : **3a-d$_1$**: **3a** = 78% : 20% : 2%, which should give 88% D-incorporation, close to experimental result: 83%.

No deuterium scrambling observed when product 3a was treated under standard conditions:

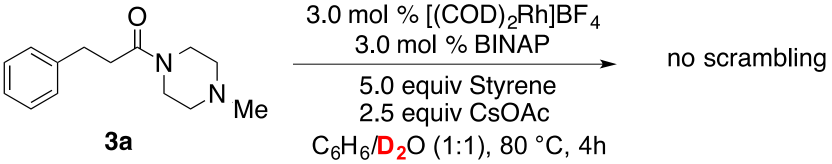

No deuterium scrambling observed when aldehyde 5a was treated under bi-phasic conditions:

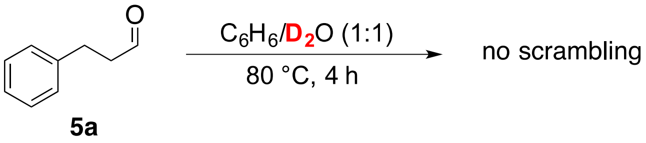

Deuterium incorporation was observed at the alpha-position of aldehyde when 20 mol% amine 2a was added:

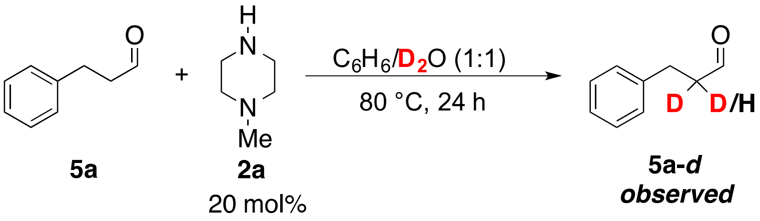

^{2}H NMR of **5a-*d***

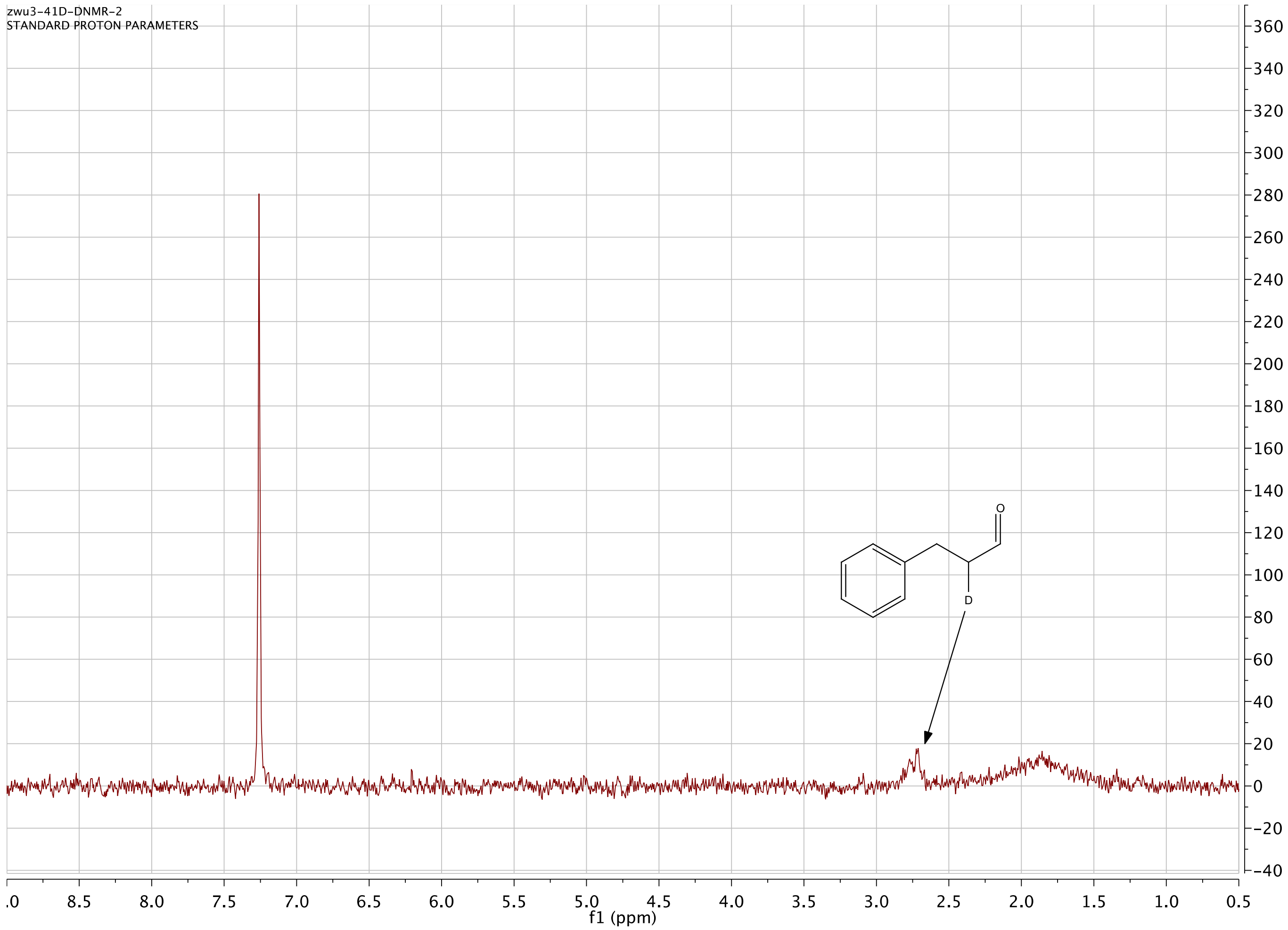

F. Kinetic Isotope Effect Study

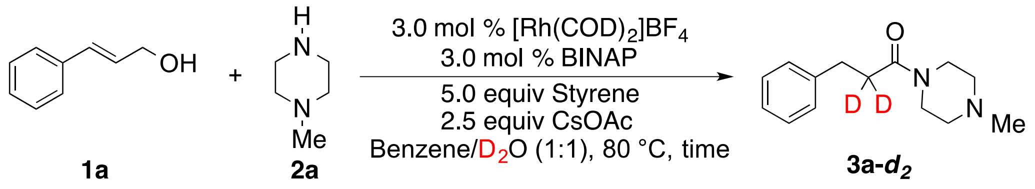

H_2O: A serial of reactions were set up according to standard procedure. After 2, 4, 6, 8, 10, and 12 min, one reaction was stopped and diluted with EtOAc, followed by the addition of diphenymethane as an internal standard. The *in situ* yield of product **3a** was determined by GC analysis and comparison to diphenylmethane as an internal standard. The yield vs. time are plotted in **Figure S1** (red).

D_2O: Same procedures were applied to D_2O, and reaction time and yield of product **3a-*d*$_2$** were plotted in **Figure S1** (blue).

Figure S1: Kinetic Isotope Effect Data

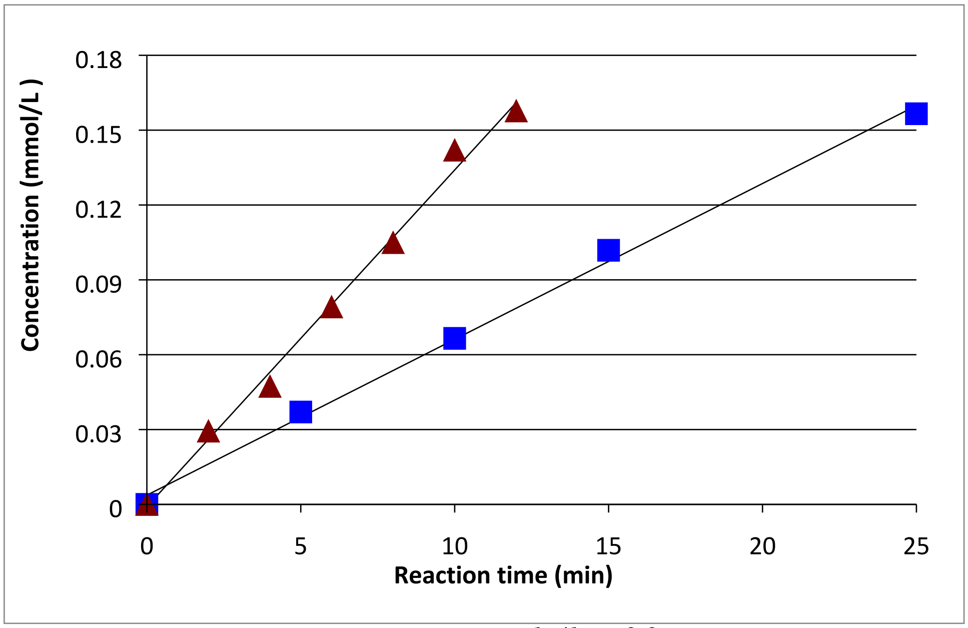

k_H/k_D = 2.2

G. Competition Studies

1) Allylic alcohols vs aldehyde

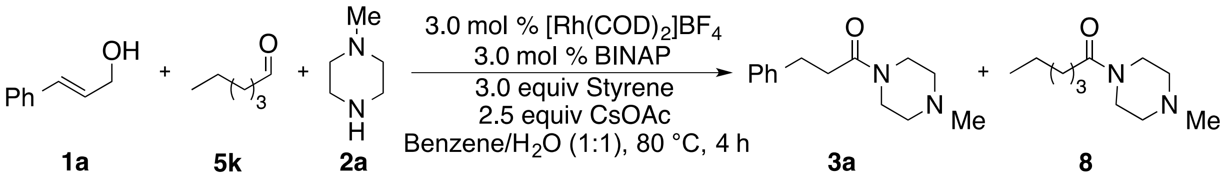

1a 1.0 equiv; **5k** 1.0 equiv; **2a** 3.0 equiv; **3a** 33%; **8** 25%

Scheme 2, Eqn 1: $[Rh(COD)_2]BF_4$ (3.1 mg, 0.0075 mmol, 3.0 mol %), BINAP (4.7 mg, 0.0075 mmol, 3.0 mol %), and CsOAc (120 mg, 0.62 mmol, 2.5 equiv) were added to a 4 mL vial equipped with a stir bar. After purging with with nitrogen, to the vial was added sequentially benzene (0.2 mL), 1-methylpiperazine (83 μL, 0.75 mmol, 3.0 equiv), styrene (87 μL, 0.75 mmol, 3.0 equiv), cinnamyl alcohol (32 μL, 0.25 mmol, 1.0 equiv), hexanal (31 μL, 0.25 mmol, 1.0 equiv) and DI water (0.2 mL). The resulting solution was allowed to stir for 4 h at 80 °C. The reaction vial was cooled to room temperature followed by the addition of diphenylmethane as an internal standard for analysis of the crude reaction mixture. The biphasic solution was added EtOAc, extracted with 1 M HCl solution three times. The combined water layer was basified by adding 2 M NaOH solution until pH > 10, then extracted with DCM three times. The combined organic layers was dried over anhydrous $MgSO_4$, filtered and concentrated *in vacuo* to afford a yellow liquid. The ratio and yield of **3a** and **8** was determined by NMR analysis.

2) Disubstituted vs. trisubstituted allylic alcohols

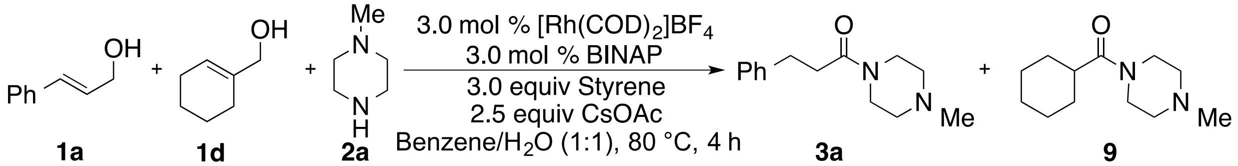

1a 1.0 equiv; **1d** 1.0 equiv; **2a** 3.0 equiv; **3a** 72%; **9** *not observed*

Scheme 2, Eqn 2: $[Rh(COD)_2]BF_4$ (3.1 mg, 0.0075 mmol, 3.0 mol %), BINAP (4.7 mg, 0.0075 mmol, 3.0 mol %), and CsOAc (120 mg, 0.62 mmol, 2.5 equiv) were added to a 4 mL vial equipped with a stir bar. After purging with with nitrogen, to the vial was added sequentially benzene (0.2 mL), 1-methylpiperazine (83 μL, 0.75 mmol, 3.0 equiv), styrene (87 μL, 0.75 mmol, 3.0 equiv), cinnamyl alcohol (32 μL, 0.25 mmol, 1.0 equiv), cyclohex-1-en-1-ylmethanol (29 μL, 0.25 mmol, 1.0 equiv) and DI water (0.2 mL). The resulting solution was allowed to stir for 4 h at 80 °C. The reaction vial was cooled to room temperature followed by the addition of diphenylmethane as an internal standard. The yields were determined by GC analysis compared to internal standard.

3) Allylic vs benzylic alcohol

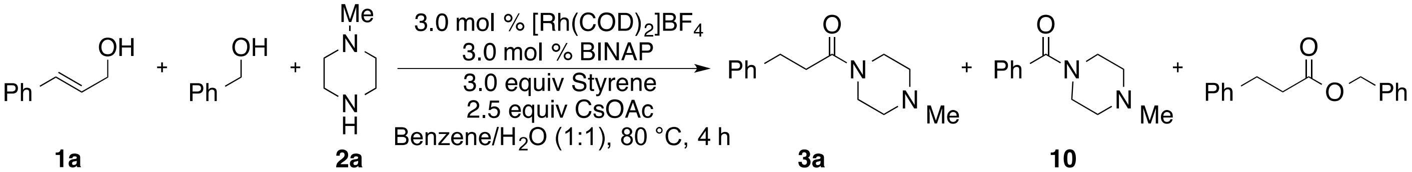

1a 1.0 equiv; 1.0 equiv; **2a** 3.0 equiv; **3a** 93%; **10** *not observed*; 7%

Scheme 2, Eqn 3: $[Rh(COD)_2]BF_4$ (3.1 mg, 0.0075 mmol, 3.0 mol %), BINAP (4.7 mg, 0.0075 mmol, 3.0 mol %), and CsOAc (120 mg, 0.62 mmol, 2.5 equiv) were added to a 4 mL vial equipped with a stir bar. After purging with with nitrogen, to the vial was added sequentially benzene (0.2 mL), 1-methylpiperazine (83 µL, 0.75 mmol, 3.0 equiv), styrene (87 µL, 0.75 mmol, 3.0 equiv), cinnamyl alcohol (32 µL, 0.25 mmol, 1.0 equiv), benzyl alcohol (26 µL, 0.25 mmol, 1.0 equiv) and DI water (0.2 mL). The resulting solution was allowed to stir for 4 h at 80 °C. The reaction vial was cooled to room temperature followed by the addition of diphenylmethane as an internal standard. The yields were determined by GC analysis compared to internal standard.

4) Allylic Vs homoallylic alcohol

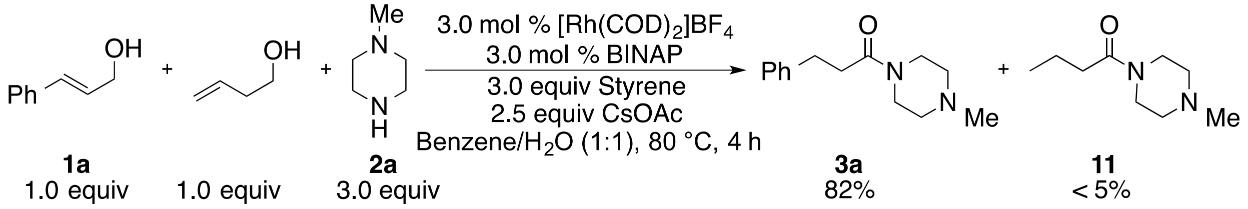

Scheme 2, Eqn 4: $[Rh(COD)_2]BF_4$ (3.1 mg, 0.0075 mmol, 3.0 mol %), BINAP (4.7 mg, 0.0075 mmol, 3.0 mol %), and CsOAc (120 mg, 0.62 mmol, 2.5 equiv) were added to a 4 mL vial equipped with a stir bar. After purging with with nitrogen, to the vial was added sequentially benzene (0.2 mL), 1-methylpiperazine (83 µL, 0.75 mmol, 3.0 equiv), styrene (87 µL, 0.75 mmol, 3.0 equiv), cinnamyl alcohol (32 µL, 0.25 mmol, 1.0 equiv), homoallylic alcohol (21 µL, 0.25 mmol, 1.0 equiv) and DI water (0.2 mL). The resulting solution was allowed to stir for 4 h at 80 °C. The reaction vial was cooled to room temperature followed by the addition of diphenylmethane as an internal standard. The yields were determined by GC analysis compared to internal standard.

5) Allylic Vs aliphatic alcohol

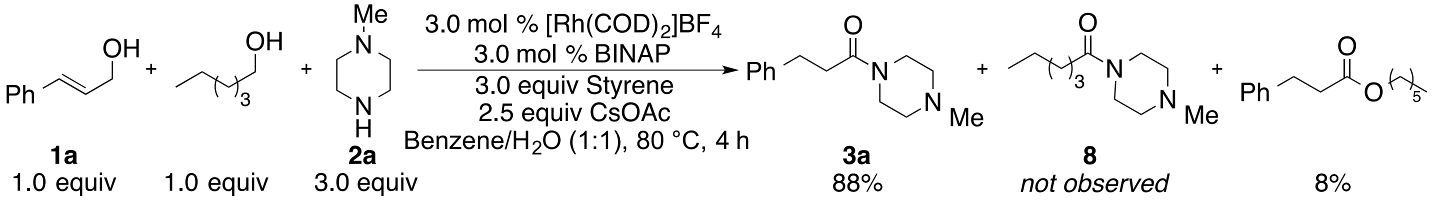

Scheme 2, Eqn 5: $[Rh(COD)_2]BF_4$ (3.1 mg, 0.0075 mmol, 3.0 mol %), BINAP (4.7 mg, 0.0075 mmol, 3.0 mol %), and CsOAc (120 mg, 0.62 mmol, 2.5 equiv) were added to a 4 mL vial equipped with a stir bar. After purging with with nitrogen, to the vial was added sequentially benzene (0.2 mL), 1-methylpiperazine (83 µL, 0.75 mmol, 3.0 equiv), styrene (87 µL, 0.75 mmol, 3.0 equiv), cinnamyl alcohol (32 µL, 0.25 mmol, 1.0 equiv), *n*-hexanol (31 µL, 0.25 mmol, 1.0 equiv) and DI water (0.2 mL). The resulting solution was allowed to stir for 4 h at 80 °C. The reaction vial was cooled to room temperature followed by the addition of diphenylmethane as an internal standard. The yields were determined by GC analysis compared to internal standard.

H. Kinetic Profile of E- vs Z- Allylic Alcohol Amidation

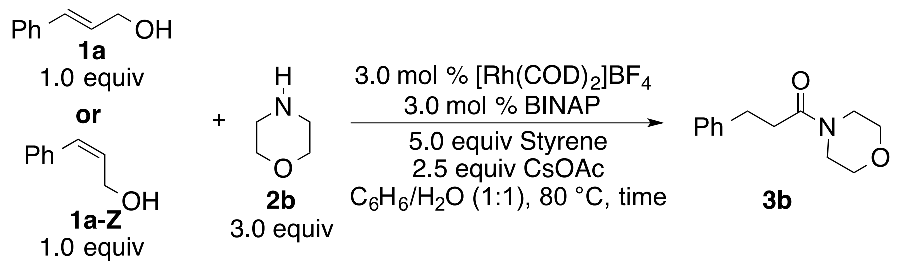

E-cinnamyl alcohol (1a): A serial of reactions were set up according to standard procedure. After 30min, 1h, 2h, 3h, 4h, 8h, and 12h, one reaction was stopped and diluted with EtOAc, followed by the addition of diphenymethane as an internal standard. The *in situ* yield of product **3a** was determined by GC analysis and comparison to diphenylmethane as an internal standard. The yields vs. time are plotted in **Figure S2** (blue).

Z-cinnamyl alcohol (1a-Z): Same procedures were applied to Z-cinnamyl alcohol, and reaction time and yield of product **3a** were plotted in **Figure S2** (red).

Figure S2: E- vs Z- Allylic Alcohol

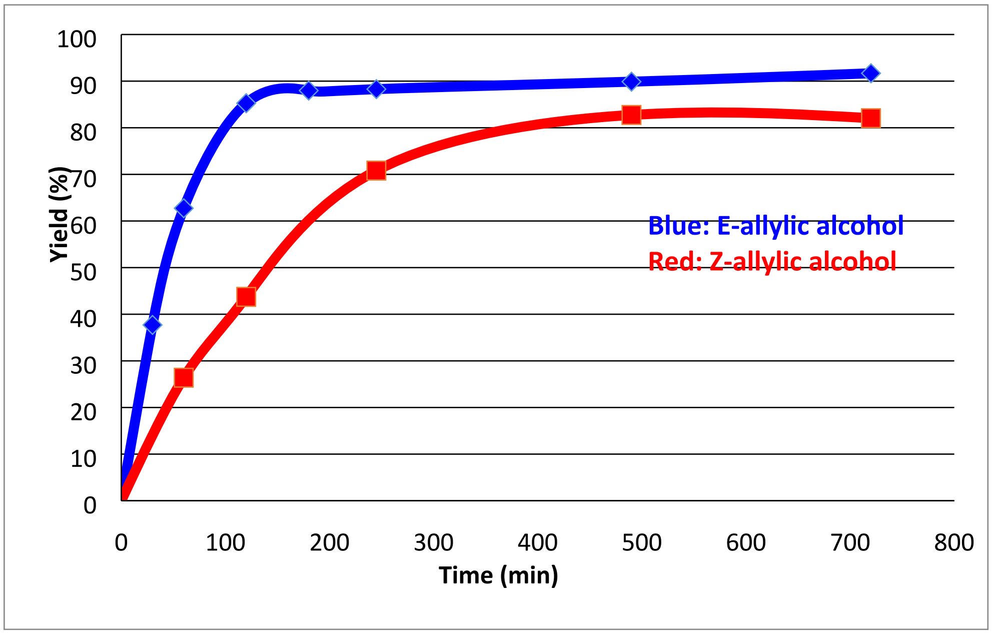

I. Experimental Procedure, Isolation, and Characterization

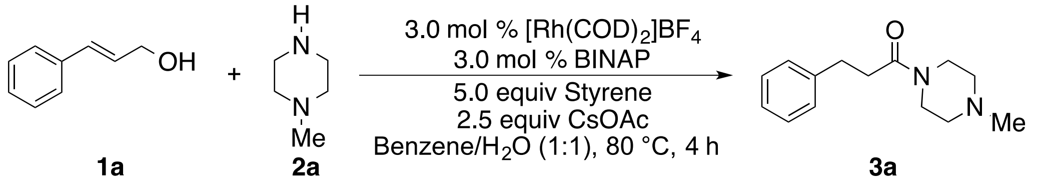

1a **2a** **3a**

1-(4-methylpiperazin-1-yl)-3-phenylpropan-1-one (Table 1, 3a): $[Rh(COD)_2]BF_4$ (3.1 mg, 0.0075 mmol, 3.0 mol %), BINAP (4.7 mg, 0.0075 mmol, 3.0 mol %), and CsOAc (120 mg, 0.62 mmol, 2.5 equiv) were added to a 4 mL vial equipped with a stir bar. After purging with with nitrogen, to the vial was added sequentially benzene (0.2 mL), 1-methylpiperazine (83 μL, 0.75 mmol, 3.0 equiv), styrene (145 μL, 1.25 mmol, 5.0 equiv), cinnamyl alcohol (32 μL, 0.25 mmol, 1.0 equiv) and DI water (0.2 mL). The resulting solution was allowed to stir for 4 h at 80 °C. The reaction vial was cooled to room temperature followed by the addition of diphenylmethane as an internal standard for analysis of the crude reaction mixture. The biphasic solution was added EtOAc, extracted with 1 M HCl solution three times. The combined water layer was basified by adding 2 M NaOH solution until pH > 10, then extracted with DCM three times. The combined organic layers was dried over anhydrous $MgSO_4$, filtered and concentrated *in vacuo* to afford a yellow liquid in 74% yield.

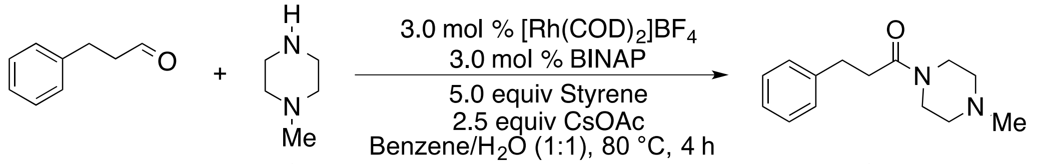

5a **2a** **3a**

1-(4-methylpiperazin-1-yl)-3-phenylpropan-1-one (Table 1, 3a): $[Rh(COD)_2]BF_4$ (3.1 mg, 0.0075 mmol, 3.0 mol %), BINAP (4.7 mg, 0.0075 mmol, 3.0 mol %), and CsOAc (120 mg, 0.62 mmol, 2.5 equiv) were added to a 4 mL vial equipped with a stir bar. After purging with with nitrogen, to the vial was added sequentially benzene (0.2 mL), 1-methylpiperazine (83 μL, 0.75 mmol, 3.0 equiv), styrene (145 μL, 1.25 mmol, 5.0 equiv), 3-phenylpropanal (34 μL, 0.25 mmol, 1.0 equiv) and DI water (0.2 mL). The resulting solution was allowed to stir for 4 h at 80 °C. The reaction vial was cooled to room temperature followed by the addition of diphenylmethane as an internal standard for analysis of the crude reaction mixture. The biphasic solution was added purified by the same acid-base extraction to afford a yellow liquid in 76% yield.

^{1}H NMR (400 MHz, $CDCl_3$) δ: 7.32 – 7.26 (m, 2H), 7.24 – 7.17 (m, 3H), 3.64 (dd, J = 6.3, 4.2 Hz, 2H), 3.39 (dd, J = 5.8, 4.3 Hz, 2H), 2.97 (dd, J = 9.2, 6.7 Hz, 2H), 2.69 – 2.55 (m, 2H), 2.35 (t, J = 5.2 Hz, 2H), 2.27 (m, 5H, overlapping peaks) ppm.

^{13}C NMR (101 MHz, $CDCl_3$) δ: 170.75, 141.39, 128.66, 128.59, 126.32, 55.12, 54.81, 46.15, 45.55, 41.65, 35.17, 31.65 ppm.

IR: ν 2940, 2795, 1643, 1440 cm^{-1}.

HRMS (ESI-TOF) *m/z*: $[M+H^+]$ calculated for $C_{14}H_{21}N_2O$, 233.1654; found, 233.1661.

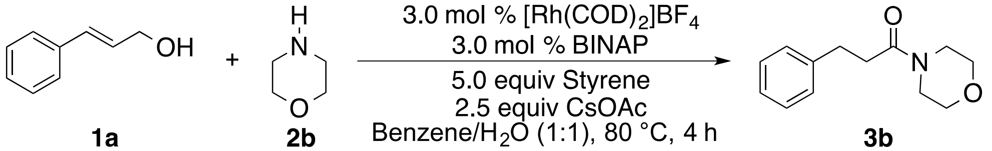

1a 2b 3b

1-morpholino-3-phenylpropan-1-one (Table 1, 3b): $[Rh(COD)_2]BF_4$ (3.1 mg, 0.0075 mmol, 3.0 mol %), BINAP (4.7 mg, 0.0075 mmol, 3.0 mol %), and CsOAc (120 mg, 0.62 mmol, 2.5 equiv) were added to a 4 mL vial equipped with a stir bar. After purging with with nitrogen, to the vial was added sequentially benzene (0.2 mL), morpholine (65 µL, 0.75 mmol, 3.0 equiv), styrene (145 µL, 1.25 mmol, 5.0 equiv), cinnamyl alcohol (32 µL, 0.25 mmol, 1.0 equiv) and DI water (0.2 mL). The resulting solution was allowed to stir for 4 h at 80 °C. Then, the reaction vial was cooled to room temperature followed by the addition of diphenylmethane as an internal standard for analysis of the crude reaction mixture. The biphasic solution was added EtOAc, dried over anhydrous $MgSO_4$, and filtered. The solution was concentrated *in vacuo* and then purified by silica gel chromatography (1:1 hexanes/EtOAc) to afford a light yellow liquid in 91% yield.

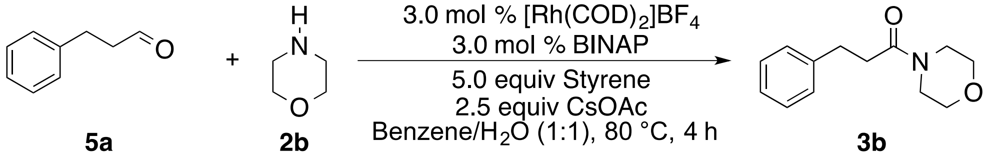

5a 2b 3b

1-morpholino-3-phenylpropan-1-one (Table 1, 3b): $[Rh(COD)_2]BF_4$ (3.1 mg, 0.0075 mmol, 3.0 mol %), BINAP (4.7 mg, 0.0075 mmol, 3.0 mol %), and CsOAc (120 mg, 1.25 mmol, 2.5 equiv) were added to a 4 mL vial equipped with a stir bar. After purging with with nitrogen, to the vial was added sequentially benzene (0.2 mL), morpholine (65 µL, 0.75 mmol, 3.0 equiv), styrene (145 µL, 1.25 mmol, 5.0 equiv), 3-phenylpropanal (34 µL, 0.25 mmol, 1.0 equiv) and DI water (0.2 mL). The resulting solution was allowed to stir for 4 h at 80 °C. Then, the reaction vial was cooled to room temperature followed by the addition of diphenylmethane as an internal standard for analysis of the crude reaction mixture. The biphasic solution was added EtOAc, dried over anhydrous $MgSO_4$, and filtered. The solution was concentrated *in vacuo* and then purified by silica gel chromatography (1:1 hexanes/EtOAc) to afford a light yellow liquid in 88% yield.

$\mathbf{R_f}$ = 0.15

^{1}H NMR (400 MHz, $CDCl_3$) δ: 7.33 – 7.27 (m, 2H), 7.25 – 7.18 (m, 3H), 3.70 – 3.58 (m, 4H), 3.51 (dd, J = 5.7, 4.0 Hz, 2H), 3.36 (dd, J = 5.7, 4.0 Hz, 2H), 2.98 (t, J = 7.8Hz, 2H), 2.62 (t, J = 7.8 Hz, 2H) ppm.

^{13}C NMR (101 MHz, $CDCl_3$) δ: 170.98, 141.17, 128.67, 128.58, 126.39, 66.97, 66.58, 46.07, 42.04, 34.94, 31.60 ppm.

IR: ν 2927, 2857, 1642, 1455, 1435, 1227, 1333 cm^{-1}.

HRMS (ESI-TOF) *m/z*: $[M+H^+]$ calculated for $C_{13}H_{17}NO_2$, 220.1338; found, 220.1342.

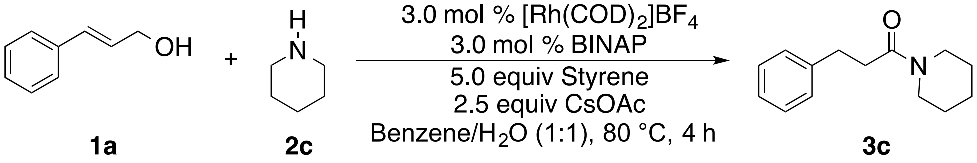

3-phenyl-1-(piperidin-1-yl)propan-1-one (Table 1, 3c): $[Rh(COD)_2]BF_4$ (3.1 mg, 0.0075 mmol, 3.0 mol %), BINAP (4.7 mg, 0.0075 mmol, 3.0 mol %), and CsOAc (120 mg, 1.25 mmol, 2.5 equiv) were added to a 4 mL vial equipped with a stir bar. After purging with with nitrogen, to the vial was added sequentially benzene (0.2 mL), piperidine (74 μL, 0.75 mmol, 3.0 equiv), styrene (145 μL, 1.25 mmol, 5.0 equiv), cinnamyl alcohol (32 μL, 0.25 mmol, 1.0 equiv) and DI water (0.2 mL). The resulting solution was allowed to stir for 4 h at 80 °C. Then, the reaction vial was cooled to room temperature followed by the addition of diphenylmethane as an internal standard for analysis of the crude reaction mixture. The biphasic solution was added EtOAc, dried over anhydrous $MgSO_4$, and filtered. The solution was concentrated *in vacuo* and then purified by silica gel chromatography (4:1 hexanes/EtOAc) to afford a colorless liquid in 84% yield.

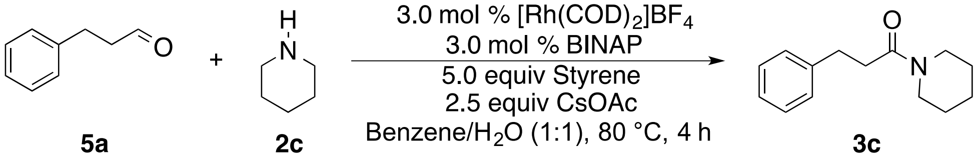

3-phenyl-1-(piperidin-1-yl)propan-1-one (Table 1, 3c): $[Rh(COD)_2]BF_4$ (3.1 mg, 0.0075 mmol, 3.0 mol %), BINAP (4.7 mg, 0.0075 mmol, 3.0 mol %), and CsOAc (120 mg, 1.25 mmol, 2.5 equiv) were added to a 4 mL vial equipped with a stir bar. After purging with with nitrogen, to the vial was added sequentially benzene (0.2 mL), morpholine (65 μL, 0.75 mmol, 3.0 equiv), styrene (145 μL, 1.25 mmol, 5.0 equiv), 3-phenylpropanal (34 μL, 0.25 mmol, 1.0 equiv) and DI water (0.2 mL). The resulting solution was allowed to stir for 4 h at 80 °C. Then, the reaction vial was cooled to room temperature followed by the addition of diphenylmethane as an internal standard for analysis of the crude reaction mixture. The biphasic solution was added EtOAc, dried over anhydrous $MgSO_4$, and filtered. The solution was concentrated *in vacuo* and then purified by silica gel chromatography (1:1 hexanes/EtOAc) to afford a light yellow liquid in 73% yield.

$\mathbf{R_f}$ = 0.2

^{1}H NMR (400 MHz, $CDCl_3$) δ: 7.29 (dd, J = 8.1, 6.8 Hz, 2H), 7.25 – 7.16 (m, 3H), 3.61 – 3.51 (m, 2H), 3.38 – 3.29 (m, 2H), 3.03 – 2.91 (m, 2H), 2.67 – 2.57 (m, 2H), 1.61 (dtt, J = 7.6, 4.7, 2.9 Hz, 2H), 1.56 – 1.41 (m, 4H) ppm.

^{13}C NMR (101 MHz, $CDCl_3$) δ: 170.53, 141.61, 128.57, 126.20, 46.74, 42.84, 35.35, 31.76, 26.52, 25.68, 24.66 ppm.

IR: ν 2937, 2858, 1639, 1441, 1252, 1218 cm^{-1}.

HRMS (ESI-TOF) *m/z*: [M+H$^+$] calculated for $C_{14}H_{20}NO$, 218.1545; found, 218.1550.

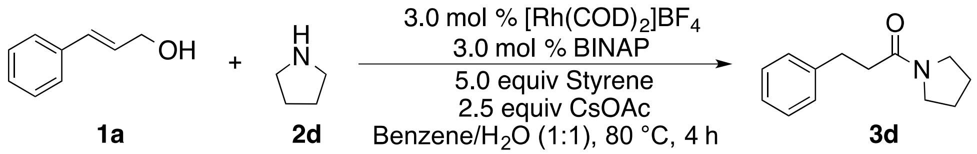

3-phenyl-1-(pyrrolidin-1-yl)propan-1-one (Table 1, 3d): $[Rh(COD)_2]BF_4$ (3.1 mg, 0.0075 mmol, 3.0 mol %), BINAP (4.7 mg, 0.0075 mmol, 3.0 mol %), and CsOAc (120 mg, 1.25 mmol, 2.5 equiv) were added to a 4 mL vial equipped with a stir bar. After purging with with nitrogen, to the vial was added sequentially benzene (0.2 mL), pyrrolidine (82 μL, 0.75 mmol, 3.0 equiv), styrene (145 μL, 1.25 mmol, 5.0 equiv), cinnamyl alcohol (32 μL, 0.25 mmol, 1.0 equiv) and DI water (0.2 mL). The resulting solution was allowed to stir for 4 h at 80 °C. Then, the reaction vial was cooled to room temperature followed by the addition of diphenylmethane as an internal standard for analysis of the crude reaction mixture. The biphasic solution was added EtOAc, dried over anhydrous $MgSO_4$, and filtered. The solution was concentrated *in vacuo* and then purified by silica gel chromatography (2:1 hexanes/EtOAc) to afford a colorless liquid in 81% yield.

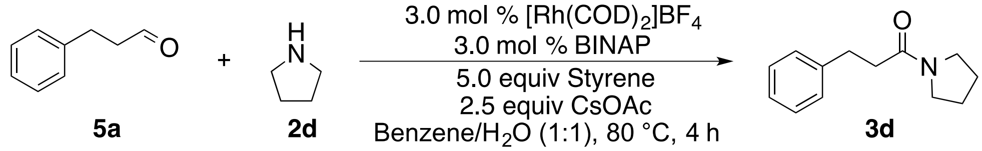

3-phenyl-1-(pyrrolidin-1-yl)propan-1-one (Table 1, 3d): $[Rh(COD)_2]BF_4$ (3.1 mg, 0.0075 mmol, 3.0 mol %), BINAP (4.7 mg, 0.0075 mmol, 3.0 mol %), and CsOAc (120 mg, 1.25 mmol, 2.5 equiv) were added to a 4 mL vial equipped with a stir bar. After purging with with nitrogen, to the vial was added sequentially benzene (0.2 mL), pyrrolidine (82 μL, 0.75 mmol, 3.0 equiv), styrene (145 μL, 1.25 mmol, 5.0 equiv), 3-phenylpropanal (34 μL, 0.25 mmol, 1.0 equiv) and DI water (0.2 mL). The resulting solution was allowed to stir for 4 h at 80 °C. Then, the reaction vial was cooled to room temperature followed by the addition of diphenylmethane as an internal standard for analysis of the crude reaction mixture. The biphasic solution was added EtOAc, dried over anhydrous $MgSO_4$, and filtered. The solution was concentrated *in vacuo* and then purified by silica gel chromatography (2:1 hexanes/EtOAc) to afford a light yellow liquid in 85% yield.

$\mathbf{R_f}$ = 0.1

^{1}H NMR (400 MHz, $CDCl_3$) δ: 7.33 – 7.25 (m, 2H), 7.25 – 7.16 (m, 3H), 3.46 (t, J = 6.7 Hz, 2H), 3.28 (t, J = 6.6 Hz, 2H), 3.06 – 2.91 (m, 2H), 2.64 – 2.50 (m, 2H), 1.96 – 1.75 (m, 4H) ppm.

^{13}C NMR (101 MHz, $CDCl_3$) δ: 170.86, 141.60, 128.54, 128.52, 126.15, 46.65, 45.75, 36.87, 31.31, 26.15, 24.48 ppm.

IR: ν 2974, 2877, 1638, 1436 cm^{-1}.

HRMS (ESI-TOF) *m/z*: [M+H$^+$] calculated for $C_{13}H_{18}NO$, 204.1388; found, 204.1396.

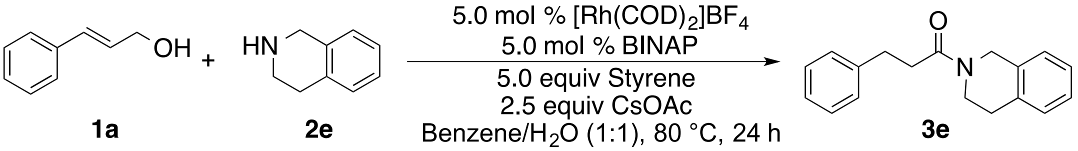

1-(3,4-dihydroisoquinolin-2(1H)-yl)-3-phenylpropan-1-one (Table 1, 3e): $[Rh(COD)_2]BF_4$ (5.0 mg, 0.0125 mmol, 5.0 mol %), BINAP (7.8 mg, 0.0125 mmol, 5.0 mol %), and CsOAc (120 mg, 1.25 mmol, 2.5 equiv) were added to a 4 mL vial equipped with a stir bar. After purging with with nitrogen, to the vial was added sequentially benzene (0.2 mL), 1,2,3,4-tetrahydroisoquinoline (95 μL, 0.75 mmol, 3.0 equiv), styrene (145 μL, 1.25 mmol, 5.0 equiv), cinnamyl alcohol (32 μL, 0.25 mmol, 1.0 equiv) and DI water (0.2 mL). The resulting solution was allowed to stir for 24 h at 80 °C. Then, the reaction vial was cooled to room temperature followed by the addition of diphenylmethane as an internal standard for analysis of the crude reaction mixture. The biphasic solution was added EtOAc, dried over anhydrous $MgSO_4$, and filtered. The solution was concentrated *in vacuo* and then purified by silica gel chromatography (4:1 hexanes/EtOAc) to afford a yellow liquid in 81% yield.

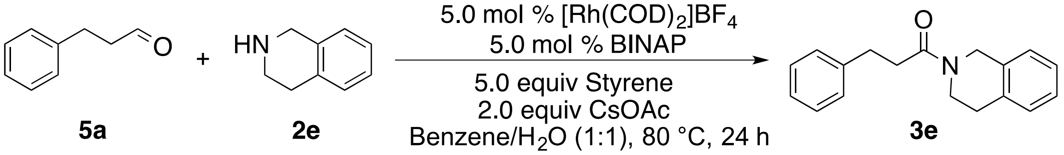

1-(3,4-dihydroisoquinolin-2(1H)-yl)-3-phenylpropan-1-one (Table 1, 3e): $[Rh(COD)_2]BF_4$ (5.0 mg, 0.0125 mmol, 5.0 mol %), BINAP (7.8 mg, 0.0125 mmol, 5.0 mol %), and CsOAc (96 mg, 1.00 mmol, 2.0 equiv) were added to a 4 mL vial equipped with a stir bar. After purging with with nitrogen, to the vial was added sequentially benzene (0.2 mL), 1,2,3,4-tetrahydroisoquinoline (95 μL, 0.75 mmol, 3.0 equiv), styrene (145 μL, 1.25 mmol, 5.0 equiv), 3-phenyl-propanal (34 μL, 0.25 mmol, 1.0 equiv) and DI water (0.2 mL). The resulting solution was allowed to stir for 24 h at 80 °C. Then, the reaction vial was cooled to room temperature followed by the addition of diphenylmethane as an internal standard for analysis of the crude reaction mixture. The biphasic solution was added EtOAc, dried over anhydrous $MgSO_4$, and filtered. The solution was concentrated *in vacuo* and then purified by silica gel chromatography (4:1 hexanes/EtOAc) to afford a yellow liquid in 72% yield.

$\mathbf{R_f}$ = 0.15

^{1}H NMR (500 MHz, DMSO-d_6, 60 °C) δ: 7.32 – 7.22 (m, 4H), 7.20 – 7.10 (m, 5H), 4.61 (s, 2H), 3.73 – 3.60 (br, 2H), 2.87 (t, J = 7.8 Hz, 2H), 2.83 – 2.74 (m, 2H), 2.71 (t, J = 7.7 Hz, 2H) ppm.

^{13}C NMR (101 MHz, $CDCl_3$) δ: 171.25, 171.13, 141.45, 135.21, 134.19, 133.68, 132.62, 129.03, 128.64, 128.61, 128.56, 128.54, 128.35, 126.99, 126.80, 126.70, 126.63, 126.42, 126.29, 126.27, 126.14, 47.38, 44.38, 43.29, 39.81, 35.95, 35.67, 31.55, 31.49, 29.55, 28.65 ppm (mixture of amide rotamers, two carbon resonances are coincidental).[1]

IR: ν 2937, 2858, 1639, 1441, 1252, 1218 cm^{-1}.

HRMS (ESI-TOF) *m/z*: $[M+H^+]$ calculated for $C_{18}H_{20}NO$, 266.1545; found, 266.1550.

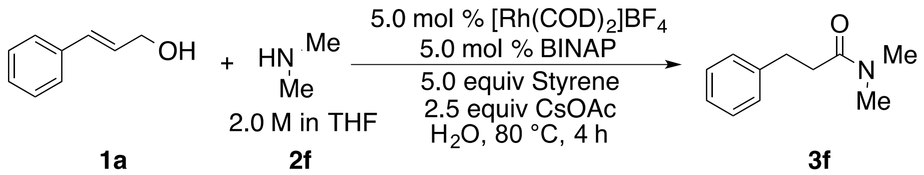

N,N-dimethyl-3-phenylpropanamide (Table 1, 3f): $[Rh(COD)_2]BF_4$ (5.0 mg, 0.0125 mmol, 5.0 mol %), BINAP (7.8 mg, 0.0125 mmol, 5.0 mol %), and CsOAc (120 mg, 1.25 mmol, 2.5 equiv) were added to a 4 mL vial equipped with a stir bar. After purging with with nitrogen, to the vial was added sequentially diethylamine (2.0 M THF) (375 µL, 0.75 mmol, 3.0 equiv), styrene (145 µL, 1.25 mmol, 5.0 equiv), cinnamyl alcohol (32 µL, 0.25 mmol, 1.0 equiv) and DI water (0.2 mL). The resulting solution was allowed to stir for 4 h at 80 °C. Then, the reaction vial was cooled to room temperature followed by the addition of diphenylmethane as an internal standard for analysis of the crude reaction mixture. The biphasic solution was added EtOAc, dried over anhydrous $MgSO_4$, and filtered. The solution was concentrated *in vacuo* and then purified by silica gel chromatography (1:1 hexanes/EtOAc) to afford a white solid in 64% yield.

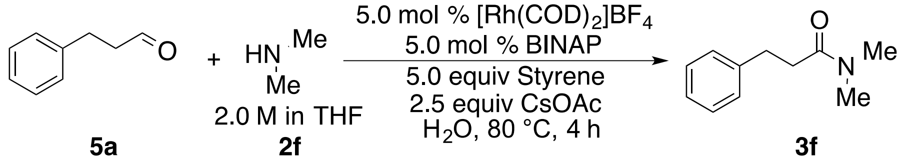

N,N-dimethyl-3-phenylpropanamide (Table 1, 3f): $[Rh(COD)_2]BF_4$ (5.0 mg, 0.0125 mmol, 5.0 mol %), BINAP (7.8 mg, 0.0125 mmol, 5.0 mol %), and CsOAc (120 mg, 1.25 mmol, 2.5 equiv) were added to a 4 mL vial equipped with a stir bar. After purging with with nitrogen, to the vial was added sequentially diethylamine (2.0 M THF) (375 µL, 0.75 mmol, 3.0 equiv), styrene (145 µL, 1.25 mmol, 5.0 equiv), 3-phenyl-propanal (34 µL, 0.25 mmol, 1.0 equiv) and DI water (0.2 mL). The resulting solution was allowed to stir for 4 h at 80 °C. Then, the reaction vial was cooled to room temperature followed by the addition of diphenylmethane as an internal standard for analysis of the crude reaction mixture. The biphasic solution was added EtOAc, dried over anhydrous $MgSO_4$, and filtered. The solution was concentrated *in vacuo* and then purified by silica gel chromatography (1:1 hexanes/EtOAc) to afford a white solid in 57% yield.

R_f = 0.25

^{1}H NMR (400 MHz, $CDCl_3$) δ: 7.32 – 7.26 (m, 2H), 7.24 – 7.17 (m, 3H), 3.00-2.96 (m, 2H), 2.95 (s, 3H), 2.93 (s, 3H), 2.66 – 2.57 (m, 2H) ppm.

^{13}C NMR (101 MHz, $CDCl_3$) δ: 172.31, 141.60, 128.57, 128.54, 126.20, 37.28, 35.56, 35.45, 31.48 ppm.

IR: ν 2936, 1644, 1496, 1455 cm^{-1}.

HRMS (ESI-TOF) *m/z*: [M+H$^+$] calculated for $C_{11}H_{26}NO$, 178.1232; found, 178.1236.

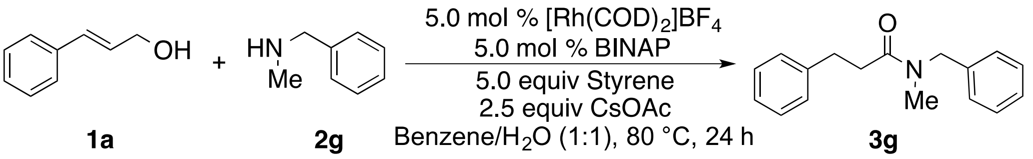

***N*-benzyl-*N*-methyl-3-phenylpropanamide (Table 1, 3g):** [Rh(COD)$_2$]BF$_4$ (5.0 mg, 0.0125 mmol, 5.0 mol %), BINAP (7.8 mg, 0.0125 mmol, 5.0 mol %), and CsOAc (120 mg, 1.25 mmol, 2.5 equiv) were added to a 4 mL vial equipped with a stir bar. After purging with with nitrogen, to the vial was added sequentially benzene (0.2 mL), *N*-methyl-1-phenylmethanamine (97 µL, 0.75 mmol, 3.0 equiv), styrene (145 µL, 1.25 mmol, 5.0 equiv), cinnamyl alcohol (32 µL, 0.25 mmol, 1.0 equiv) and DI water (0.2 mL). The resulting solution was allowed to stir for 24 h at 80 °C. Then, the reaction vial was cooled to room temperature followed by the addition of diphenylmethane as an internal standard for analysis of the crude reaction mixture. The biphasic solution was added EtOAc, dried over anhydrous MgSO$_4$, and filtered. The solution was concentrated *in vacuo* and then purified by silica gel chromatography (6:1 hexanes/EtOAc) to afford a colorless liquid in 76% yield.

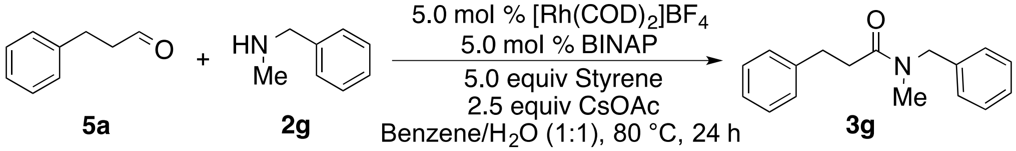

***N*-benzyl-*N*-methyl-3-phenylpropanamide (Table 1, 3g):** [Rh(COD)$_2$]BF$_4$ (5.0 mg, 0.0125 mmol, 5.0 mol %), BINAP (7.8 mg, 0.0125 mmol, 5.0 mol %), and CsOAc (120 mg, 1.25 mmol, 2.5 equiv) were added to a 4 mL vial equipped with a stir bar. After purging with with nitrogen, to the vial was added sequentially benzene (0.2 mL), *N*-methyl-1-phenylmethanamine (97 µL, 0.75 mmol, 3.0 equiv), styrene (145 µL, 1.25 mmol, 5.0 equiv), 3-phenyl-propanal (34 µL, 0.25 mmol, 1.0 equiv) and DI water (0.2 mL). The resulting solution was allowed to stir for 24 h at 80 °C. Then, the reaction vial was cooled to room temperature followed by the addition of diphenylmethane as an internal standard for analysis of the crude reaction mixture. The biphasic solution was added EtOAc, dried over anhydrous MgSO$_4$, and filtered. The solution was concentrated *in vacuo* and then purified by silica gel chromatography (6:1 hexanes/EtOAc) to afford a colorless liquid in 76% yield.

R_f = 0.15

^{1}H NMR (400 MHz, CDCl$_3$, mixture of amide rotamers)[2] δ: 7.42 – 7.15 (m, 9H), 7.13 – 7.05 (m, 1H), 4.61 (s, 1.1H), 4.47 (s, 0.8H), 3.09 – 2.98 (m, 2H), 2.96 (s, 1.1H), 2.85 (s, 1.8H), 2.74 – 2.65 (m, 2H) ppm.

^{13}C NMR (101 MHz, CDCl$_3$, mixture of amide rotamers)[2] δ: 172.69, 172.36, 141.49, 141.41, 137.47, 136.64, 129.02, 128.67, 128.59, 128.57, 128.15, 127.68, 127.42, 126.33, 126.24, 126.22, 53.34, 50.96, 35.52, 35.09, 34.85, 34.10, 31.66, 31.49 ppm.

IR: ν 2926, 2856, 1644, 1495, 1453 cm^{-1}.

HRMS (ESI-TOF) *m/z*: [M+H$^+$] calculated for $C_{17}H_{20}NO$, 254.1545; found, 254.1547.

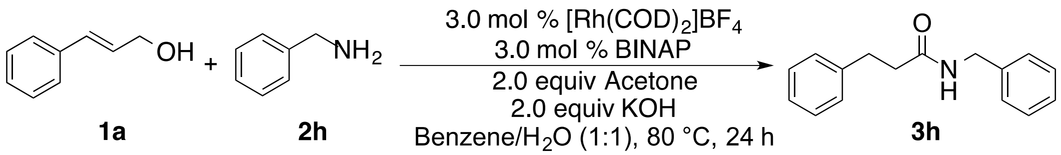

N-benzyl-3-phenylpropanamide (Table 1, 3h): $[Rh(COD)_2]BF_4$ (3.1 mg, 0.0075 mmol, 3.0 mol %), BINAP (4.7 mg, 0.0075 mmol, 3.0 mol %), and KOH (28 mg, 0.50 mmol, 2.0 equiv) were added to a 4 mL vial equipped with a stir bar. After purging with with nitrogen, to the vial was added sequentially benzene (0.2 mL), benzylamine (82 µL, 0.75 mmol, 3.0 equiv), acetone (36 µL, 0.50 mmol, 2.0 equiv), cinnamyl alcohol (32 µL, 0.25 mmol, 1.0 equiv) and DI water (0.2 mL). The resulting solution was allowed to stir for 24 h at 80 °C. Then, the reaction vial was cooled to room temperature followed by the addition of diphenylmethane as an internal standard for analysis of the crude reaction mixture. The biphasic solution was added EtOAc, dried over anhydrous $MgSO_4$, and filtered. The solution was concentrated *in vacuo* and then purified by silica gel chromatography (4:1 hexanes/EtOAc) to afford a white solid in 80% yield.

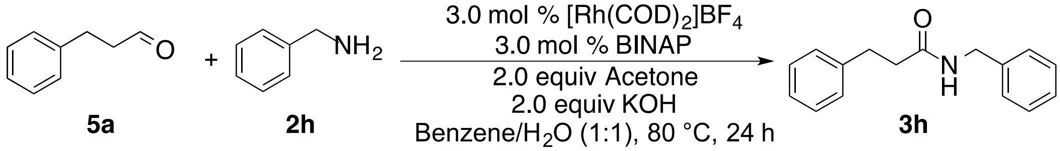

N-benzyl-3-phenylpropanamide (Table 1, 3h): $[Rh(COD)_2]BF_4$ (3.1 mg, 0.0075 mmol, 3.0 mol %), BINAP (4.7 mg, 0.0075 mmol, 3.0 mol %), and KOH (28 mg, 0.50 mmol, 2.0 equiv) were added to a 4 mL vial equipped with a stir bar. After purging with with nitrogen, to the vial was added sequentially benzene (0.2 mL), benzylamine (82 µL, 0.75 mmol, 3.0 equiv), acetone (36 µL, 0.50 mmol, 2.0 equiv), 3-phenyl-propanal (34 µL, 0.25 mmol, 1.0 equiv) and DI water (0.2 mL). The resulting solution was allowed to stir for 24 h at 80 °C. Then, the reaction vial was cooled to room temperature followed by the addition of diphenylmethane as an internal standard for analysis of the crude reaction mixture. The biphasic solution was added EtOAc, dried over anhydrous $MgSO_4$, and filtered. The solution was concentrated *in vacuo* and then purified by silica gel chromatography (4:1 hexanes/EtOAc) to afford a white solid in 72% yield.

R_f = 0.25

mp = 80-83 °C (reported mp = 81-83 °C)[3]

^{1}H NMR (400 MHz, $CDCl_3$) δ: 7.33 – 7.23 (m, 5H), 7.23 – 7.18 (m, 3H), 7.18 – 7.12 (m, 2H), 5.59 (bs, 1H), 4.40 (d, *J* = 5.7 Hz, 2H), 3.00 (t, *J* = 7.6 Hz, 2H), 2.52 (t, *J* = 7.6 Hz, 2H) ppm.

^{13}C NMR (101 MHz, $CDCl_3$) δ: 171.94, 140.89, 138.24, 128.80, 128.70, 128.54, 127.89, 127.61, 126.40, 43.73, 38.71, 31.87 ppm.

IR: ν 3293, 3031, 2928, 1639, 1545 cm^{-1}.

HRMS (ESI-TOF) *m/z*: $[M+H^+]$ calculated for $C_{16}H_{18}NO$, 240.1388; found, 240.1393.

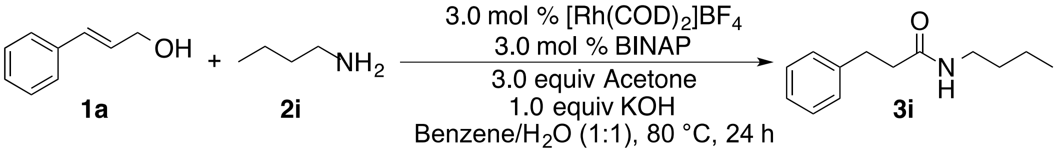

***N*-Butyl-3-phenylpropanamide (Table 1, 3i):** $[Rh(COD)_2]BF_4$ (3.1 mg, 0.0075 mmol, 3.0 mol %), BINAP (4.7 mg, 0.0075 mmol, 3.0 mol %), and KOH (14 mg, 0.25 mmol, 1.0 equiv) were added to a 4 mL vial equipped with a stir bar. After purging with with nitrogen, to the vial was added sequentially benzene (0.2 mL), *n*-butylamine (74 µL, 0.75 mmol, 3.0 equiv), acetone (54 µL, 0.75 mmol, 3.0 equiv), cinnamyl alcohol (32 µL, 0.25 mmol, 1.0 equiv) and DI water (0.2 mL). The resulting solution was allowed to stir for 24 h at 80 °C. Then, the reaction vial was cooled to room temperature followed by the addition of diphenylmethane as an internal standard for analysis of the crude reaction mixture. The biphasic solution was added EtOAc, dried over anhydrous $MgSO_4$, and filtered. The solution was concentrated *in vacuo* and then purified by silica gel chromatography (3:1 hexanes/EtOAc) to afford a light yellow liquid in 72% yield.

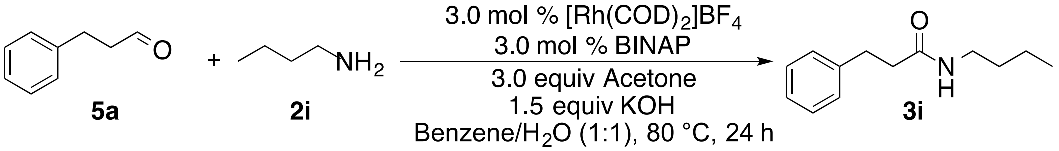

***N*-Butyl-3-phenylpropanamide (Table 1, 3i):** $[Rh(COD)_2]BF_4$ (3.1 mg, 0.0075 mmol, 3.0 mol %), BINAP (4.7 mg, 0.0075 mmol, 3.0 mol %), and KOH (21 mg, 0.38 mmol, 1.5 equiv) were added to a 4 mL vial equipped with a stir bar. After purging with with nitrogen, to the vial was added sequentially benzene (0.2 mL), *n*-butylamine (74 µL, 0.75 mmol, 3.0 equiv), acetone (54 µL, 0.75 mmol, 3.0 equiv), 3-phenyl-propanal (34 µL, 0.25 mmol, 1.0 equiv) and DI water (0.2 mL). The resulting solution was allowed to stir for 24 h at 80 °C. Then, the reaction vial was cooled to room temperature followed by the addition of diphenylmethane as an internal standard for analysis of the crude reaction mixture. The biphasic solution was added EtOAc, dried over anhydrous $MgSO_4$, and filtered. The solution was concentrated *in vacuo* and then purified by silica gel chromatography (3:1 hexanes/EtOAc) to afford a light yellow liquid in 63% yield.

$\mathbf{R_f}$ = 0.2

^{1}H NMR (400 MHz, $CDCl_3$) δ: 7.30 – 7.24 (m, 2H), 7.21 – 7.14 (m, 3H), 5.28 (bs, 1H), 3.18 (td, J = 7.2, 5.7 Hz, 2H), 2.94 (t, J = 7.7 Hz, 2H), 2.44 (t, J = 7.7 Hz, 2H), 1.46 – 1.30 (m, 2H), 1.31 – 1.16 (m, 2H), 0.86 (t, J = 7.3 Hz, 3H) ppm.

^{13}C NMR (126 MHz, $CDCl_3$) δ: 172.12, 141.03, 128.62, 128.47, 126.32, 39.35, 38.74, 31.94, 31.74, 20.10, 13.86 ppm.

IR: ν 3301, 2962, 2932, 1643, 1554 cm^{-1}.

HRMS (ESI-TOF) *m/z*: $[M+H^+]$ calculated for $C_{13}H_{20}NO$, 206.1545; found, 206.1548.

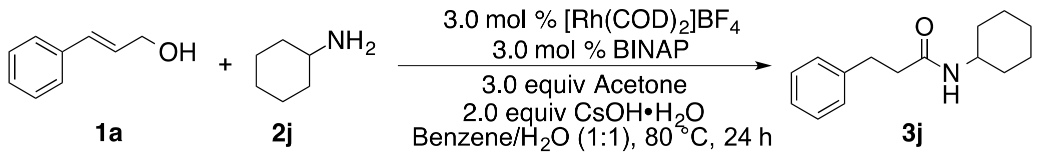

***N*-Cyclohexyl-3-phenylpropanamide (Table 3j):** $[Rh(COD)_2]BF_4$ (3.1 mg, 0.0075 mmol, 3.0 mol %), BINAP (4.7 mg, 0.0075 mmol, 3.0 mol %), and $CsOH•H_2O$ (84 mg, 0.50 mmol, 2.0 equiv) were added to a 4 mL vial equipped with a stir bar. After purging with with nitrogen, to the vial was added sequentially benzene (0.2 mL), cyclohexylamine (86 μL, 0.75 mmol, 3.0 equiv), acetone (54 μL, 0.75 mmol, 3.0 equiv), cinnamyl alcohol (32 μL, 0.25 mmol, 1.0 equiv) and DI water (0.2 mL). The resulting solution was allowed to stir for 24 h at 80 °C. Then, the reaction vial was cooled to room temperature followed by the addition of diphenylmethane as an internal standard for analysis of the crude reaction mixture. The biphasic solution was added EtOAc, dried over anhydrous $MgSO_4$, and filtered. The solution was concentrated *in vacuo* and then purified by silica gel chromatography (4:1 hexanes/EtOAc) to afford a white solid in 54% yield.

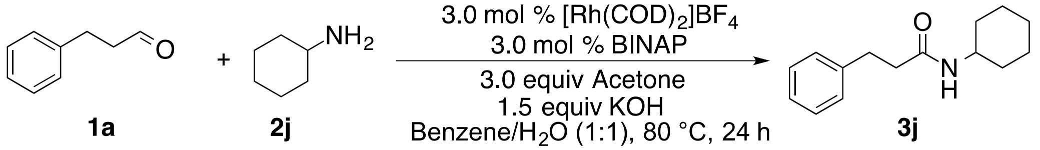

***N*-Cyclohexyl-3-phenylpropanamide (Table 1, 3j):** $[Rh(COD)_2]BF_4$ (3.1 mg, 0.0075 mmol, 3.0 mol %), BINAP (4.7 mg, 0.0075 mmol, 3.0 mol %), and KOH (21 mg, 0.38 mmol, 1.5 equiv) were added to a 4 mL vial equipped with a stir bar. After purging with with nitrogen, to the vial was added sequentially benzene (0.2 mL), *n*-butylamine (74 μL, 0.75 mmol, 3.0 equiv), acetone (54 μL, 0.75 mmol, 3.0 equiv), 3-phenyl-propanal (34 μL, 0.25 mmol, 1.0 equiv) and DI water (0.2 mL). The resulting solution was allowed to stir for 24 h at 80 °C. Then, the reaction vial was cooled to room temperature followed by the addition of diphenylmethane as an internal standard for analysis of the crude reaction mixture. The biphasic solution was added EtOAc, dried over anhydrous $MgSO_4$, and filtered. The solution was concentrated *in vacuo* and then purified by silica gel chromatography (4:1 hexanes/EtOAc) to afford a white solid in 48% yield.

R_f = 0.2

mp = 107-108 °C (reported mp = 113-114 °C)[4]

^{1}H NMR (500 MHz, $CDCl_3$) δ: 7.31 – 7.26 (m, 2H), 7.23 – 7.17 (m, 3H), 5.12 (s, 1H), 3.79– 3.69 (m, 1H), 2.96 (t, J = 7.7 Hz, 2H), 2.43 (t, J = 7.6 Hz, 2H), 1.87 – 1.78 (m, 2H), 1.69 – 1.61 (m, 2H), 1.58 (ddd, J = 7.8, 3.9 Hz, 1H), 1.39 – 1.28 (m, 2H), 1.11 (ddt, J = 12.8, 3.5 Hz, 1H), 1.00 (ddd, J = 12.0, 3.6 Hz, 2H) ppm.

^{13}C NMR (126 MHz, $CDCl_3$) δ: 171.13, 141.07, 128.63, 128.55, 126.34, 48.17, 39.00, 33.27, 32.04, 25.65, 24.95 ppm.

IR: ν 3303, 2939, 2850, 1636, 1543 cm^{-1}.

HRMS (ESI-TOF) *m/z*: $[M+H^+]$ calculated for $C_{15}H_{22}NO$, 232.1701; found, 232.1704.

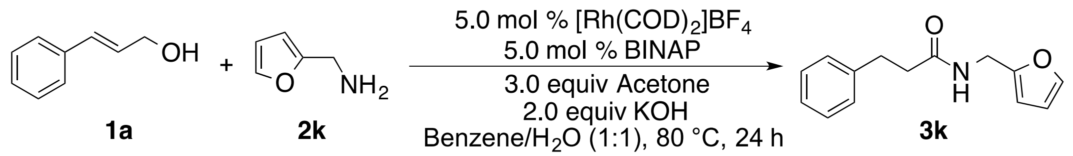

***N*-(furan-2-ylmethyl)-3-phenylpropanamide (Table 1, 3k)** $[Rh(COD)_2]BF_4$ (5.0 mg, 0.0125 mmol, 5.0 mol %) and BINAP (7.8 mg, 0.0125 mmol, 5.0 mol %) and KOH (28 mg, 0.50 mmol, 2.0 equiv) were added to a 4 mL vial equipped with a stir bar. After purging with with nitrogen, to the vial was added sequentially benzene (0.2 mL), furan-2-ylmethanamine (70 µL, 0.75 mmol, 3.0 equiv), acetone (54 µL, 0.75 mmol, 3.0 equiv), cinnamyl alcohol (32 µL, 0.25 mmol, 1.0 equiv) and DI water (0.2 mL). The resulting solution was allowed to stir for 24 h at 80 °C. Then, the reaction vial was cooled to room temperature followed by the addition of diphenylmethane as an internal standard for analysis of the crude reaction mixture. The biphasic solution was added EtOAc, dried over anhydrous $MgSO_4$, and filtered. The solution was concentrated *in vacuo* and then purified by silica gel chromatography (4:1 hexanes/EtOAc) to afford a white solid in 73% yield.

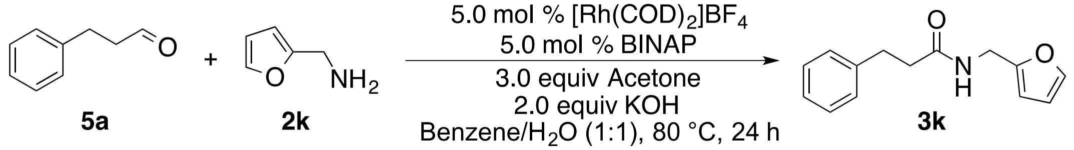

***N*-(furan-2-ylmethyl)-3-phenylpropanamide (Table 1, 3k)** $[Rh(COD)_2]BF_4$ (5.0 mg, 0.0125 mmol, 5.0 mol %) and BINAP (7.8 mg, 0.0125 mmol, 5.0 mol %) and KOH (28 mg, 0.50 mmol, 2.0 equiv) were added to a 4 mL vial equipped with a stir bar. After purging with with nitrogen, to the vial was added sequentially benzene (0.2 mL), furan-2-ylmethanamine (70 µL, 0.75 mmol, 3.0 equiv), acetone (54 µL, 0.75 mmol, 3.0 equiv), 3-phenyl-propanal (34 µL, 0.25 mmol, 1.0 equiv) and DI water (0.2 mL). The resulting solution was allowed to stir for 24 h at 80 °C. Then, the reaction vial was cooled to room temperature followed by the addition of diphenylmethane as an internal standard for analysis of the crude reaction mixture. The biphasic solution was added EtOAc, dried over anhydrous $MgSO_4$, and filtered. The solution was concentrated *in vacuo* and then purified by silica gel chromatography (4:1 hexanes/EtOAc) to afford a white solid in 66% yield.

$\mathbf{R_f}$ = 0.1

mp = 51-53 °C (reported mp = 56-58 °C)[10]

^{1}H NMR (400 MHz, $CDCl_3$) δ: 7.46 – 7.10 (m, 6H), 6.46 – 6.21 (m, 1H), 6.16 (d, J = 3.3 Hz, 1H), 5.62 (bs, 1H), 4.41 (d, J = 5.6 Hz, 2H), 2.98 (t, J = 7.7 Hz, 2H), 2.49 (t, J = 7.7 Hz, 2H).

^{13}C NMR (126 MHz, $CDCl_3$) δ: 171.88, 151.32, 142.30, 140.87, 128.67, 128.47, 126.37, 110.57, 107.57, 38.52, 36.60, 31.74.

IR: ν 3311, 2961, 2930, 2869, 1638, 1534.

HRMS (ESI-TOF) *m/z*: [M+H$^+$] calculated for $C_{13}H_{20}NO$, 230.1181; found, 230.1174.

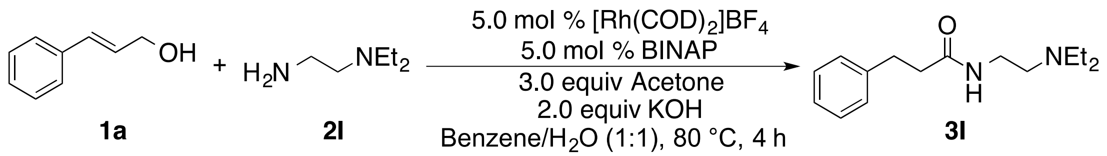

N-(2-(diethylamino)ethyl)-3-phenylpropanamide (Table 1, 3l): $[Rh(COD)_2]BF_4$ (5.0 mg, 0.0125 mmol, 5.0 mol %) and BINAP (7.8 mg, 0.0125 mmol, 5.0 mol %) and KOH (28 mg, 0.50 mmol, 2.0 equiv) were added to a 4 mL vial equipped with a stir bar. After purging with with nitrogen, to the vial was added sequentially benzene (0.2 mL), N, N-diethylethane-1,2-diamine (105 μL, 0.75 mmol, 3.0 equiv), acetone (54 μL, 0.75 mmol, 3.0 equiv), cinnamyl alcohol (32 μL, 0.25 mmol, 1.0 equiv) and DI water (0.2 mL). The resulting solution was allowed to stir for 4 h at 80 °C. Then, the reaction vial was cooled to room temperature followed by the addition of diphenylmethane as an internal standard for analysis of the crude reaction mixture. The biphasic solution was added EtOAc, extracted with 1 M HCl solution three times. The combined water layer was basified by adding 2 M NaOH solution until pH > 10, then extracted with DCM three times. The combine organic layers was dried over anhydrous $MgSO_4$, filtered and concentrated *in vacuo* to afford a yellow liquid in 73% yield.

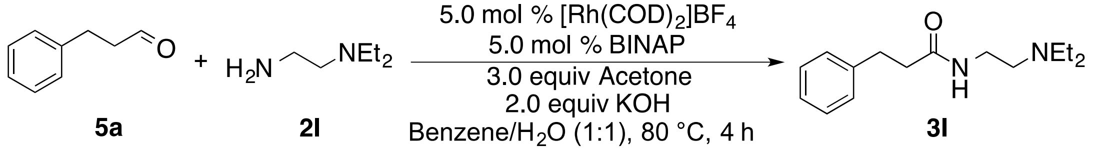

N-(2-(diethylamino)ethyl)-3-phenylpropanamide (Table 1, 3l): $[Rh(COD)_2]BF_4$ (5.0 mg, 0.0125 mmol, 5.0 mol %) and BINAP (7.8 mg, 0.0125 mmol, 5.0 mol %) and KOH (28 mg, 0.50 mmol, 2.0 equiv) were added to a 4 mL vial equipped with a stir bar. After purging with with nitrogen, to the vial was added sequentially benzene (0.2 mL), N, N-diethylethane-1,2-diamine (105 μL, 0.75 mmol, 3.0 equiv), acetone (54 μL, 0.75 mmol, 3.0 equiv), 3-phenyl-propanal (34 μL, 0.25 mmol, 1.0 equiv) and DI water (0.2 mL). The resulting solution was allowed to stir for 4 h at 80 °C. Then, the reaction vial was cooled to room temperature followed by the addition of diphenylmethane as an internal standard for analysis of the crude reaction mixture. The biphasic solution was added EtOAc, extracted with 1 M HCl solution three times. The combined water layer was basified by adding 2 M NaOH solution until pH > 10, then extracted with DCM three times. The combine organic layers was dried over anhydrous $MgSO_4$, filtered and concentrated *in vacuo* to afford a yellow liquid in 65% yield.

$\mathbf{R_f}$ = 0.1

^{1}H NMR (400 MHz, Benzene-d_6) δ: 7.15 – 7.09 (m, 2H), 7.09 – 7.00 (m, 3H), 5.39 (bs, 1H), 3.20 (q, J = 5.7 Hz, 2H), 2.95 (t, J = 7.6 Hz, 2H), 2.19 (q, J = 7.1 Hz, 4H), 2.15 – 2.08 (m, 4H), 0.78 (t, J = 7.1 Hz, 6H).

^{13}C NMR (101 MHz, $CDCl_3$) δ: δ 172.09, 141.12, 128.60, 128.47, 126.28, 51.41, 46.70, 38.64, 36.91, 31.91, 11.84.

IR: ν 3304, 2971, 2937, 1647, 1554.

HRMS (ESI-TOF) *m/z*: $[M+H^+]$ calculated for $C_{13}H_{20}NO$, 246.1967; found, 246.1968.

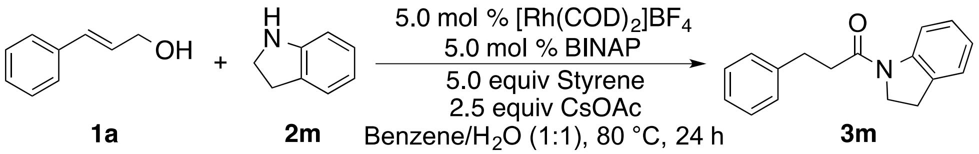

1-(indolin-1-yl)-3-phenylpropan-1-one (Table 1, 3m): $[Rh(COD)_2]BF_4$ (5.0 mg, 0.0125 mmol, 5.0 mol %), BINAP (7.8 mg, 0.0125 mmol, 5.0 mol %), and CsOAc (120 mg, 1.25 mmol, 2.5 equiv) were added to a 4 mL vial equipped with a stir bar. After purging with with nitrogen, to the vial was added sequentially benzene (0.2 mL), indoline (84 μL, 0.75 mmol, 3.0 equiv), styrene (145 μL, 1.25 mmol, 5.0 equiv), cinnamyl alcohol (32 μL, 0.25 mmol, 1.0 equiv) and DI water (0.2 mL). The resulting solution was allowed to stir for 24 h at 80 °C. Then, the reaction vial was cooled to room temperature followed by the addition of diphenylmethane as an internal standard for analysis of the crude reaction mixture. The biphasic solution was added EtOAc, extracted with 1 M HCl three times. The combined organic layer was dried over anhydrous $MgSO_4$, filtered and concentrated *in vacuo* and then purified by silica gel chromatography (10:1 hexanes/EtOAc) to afford a white solid in 73% yield.

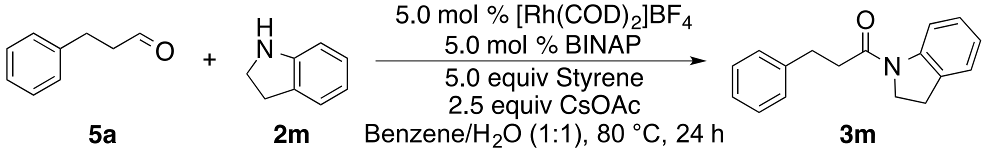

1-(indolin-1-yl)-3-phenylpropan-1-one (Table 1, 3m): $[Rh(COD)_2]BF_4$ (5.0 mg, 0.0125 mmol, 5.0 mol %), BINAP (7.8 mg, 0.0125 mmol, 5.0 mol %), and CsOAc (120 mg, 1.25 mmol, 2.5 equiv) were added to a 4 mL vial equipped with a stir bar. After purging with with nitrogen, to the vial was added sequentially benzene (0.2 mL), indoline (84 μL, 0.75 mmol, 3.0 equiv), styrene (145 μL, 1.25 mmol, 5.0 equiv), 3-phenyl-propanal (34 μL, 0.25 mmol, 1.0 equiv) and DI water (0.2 mL). The resulting solution was allowed to stir for 24 h at 80 °C. Then, the reaction vial was cooled to room temperature followed by the addition of diphenylmethane as an internal standard for analysis of the crude reaction mixture. The biphasic solution was added EtOAc, extracted with 1 M HCl three times. The combined organic layer was dried over anhydrous $MgSO_4$, filtered and concentrated *in vacuo* and then purified by silica gel chromatography (10:1 hexanes/EtOAc) to afford a white solid in 83% yield.

R_f = 0.1 **mp** =112-113 °C

^{1}H NMR (500 MHz, $CDCl_3$, mixture of amide rotamers)[9] δ: 8.27 (d, J = 8.0 Hz, 1H), 7.43 – 7.25 (m, 4H), 7.20 (m, 3H, overlapping peaks), 7.01 (t, J = 7.4 Hz, 1H), 4.16 (t, J = 7.9 Hz, 0.2H, minor rotamer), 3.97 (t, J = 8.5 Hz, 2H, major rotamer), 3.15 (t, J = 8.5 Hz, 2H), 3.08 (t, J = 7.8 Hz, 2H, major rotamer), 2.99 (t, J = 8.0 Hz, 0.2H minor rotamer).2.74 (t, J = 7.9 Hz 2H).

^{13}C NMR (126 MHz, $CDCl_3$) (major rotamer) δ: 170.50, 143.12, 141.37, 131.16, 128.68, 128.59, 127.69, 126.33, 124.63, 123.71, 117.16, 48.08, 38.07, 30.92, 28.16.

IR: ν 3064, 2929, 1654, 1483.

HRMS (ESI-TOF) *m/z*: $[M+H^+]$ calculated for $C_{17}H_{18}NO$, 252.1388; found, 252.1390.

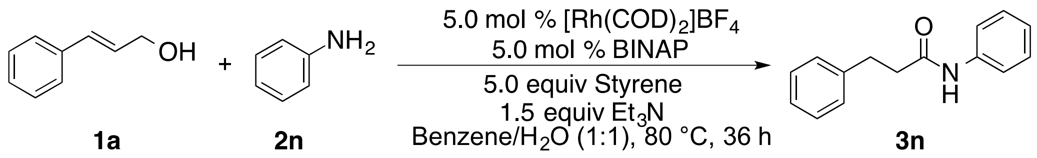

***N*,3-diphenylpropanamide (Table 1, 3n):** $[Rh(COD)_2]BF_4$ (5.0 mg, 0.0125 mmol, 5.0 mol %) and BINAP (7.8 mg, 0.0125 mmol, 5.0 mol %) were added to a 4 mL vial equipped with a stir bar. After purging with with nitrogen, to the vial was added sequentially benzene (0.2 mL), aniline (68 µL, 0.75 mmol, 3.0 equiv), styrene (145 µL, 1.25 mmol, 5.0 equiv), triethylamine (53 µL, 0.38 mmol, 1.5 equiv), cinnamyl alcohol (32 µL, 0.25 mmol, 1.0 equiv) and DI water (0.2 mL). The resulting solution was allowed to stir for 36 h at 80 °C. Then, the reaction vial was cooled to room temperature followed by the addition of diphenylmethane as an internal standard for analysis of the crude reaction mixture. The biphasic solution was added EtOAc, dried over anhydrous $MgSO_4$, and filtered. The solution was concentrated *in vacuo* and then purified by silica gel chromatography (4:1 hexanes/EtOAc) to afford a white solid in 55% yield.

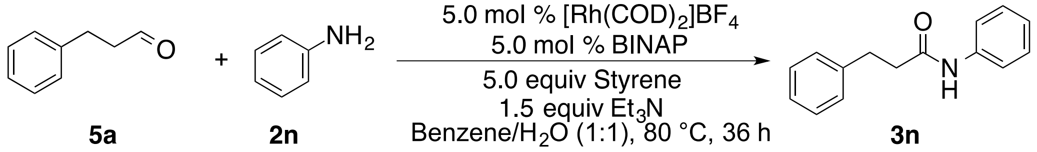

***N*,3-diphenylpropanamide (Table 1, 3n):** $[Rh(COD)_2]BF_4$ (5.0 mg, 0.0125 mmol, 5.0 mol %) and BINAP (7.8 mg, 0.0125 mmol, 5.0 mol %) were added to a 4 mL vial equipped with a stir bar. After purging with with nitrogen, to the vial was added sequentially benzene (0.2 mL), aniline (68 µL, 0.75 mmol, 3.0 equiv), styrene (145 µL, 1.25 mmol, 5.0 equiv), triethylamine (53 µL, 0.38 mmol, 1.5 equiv), 3-phenyl-propanal (34 µL, 0.25 mmol, 1.0 equiv) and DI water (0.2 mL). The resulting solution was allowed to stir for 36 h at 80 °C. Then, the reaction vial was cooled to room temperature followed by the addition of diphenylmethane as an internal standard for analysis of the crude reaction mixture. The biphasic solution was added EtOAc, dried over anhydrous $MgSO_4$, and filtered. The solution was concentrated *in vacuo* and then purified by silica gel chromatography (4:1 hexanes/EtOAc) to afford a white solid in 59% yield.

$\mathbf{R_f}$ = 0.3

mp = 92-94 °C (reported mp = 95-96 °C)[5]

^{1}H NMR (500 MHz, $CDCl_3$) δ: 7.43 (d, J = 8.0 Hz, 2H), 7.34 – 7.27 (m, 4H), 7.25 – 7.20 (m, 3H), 7.10 (t, J = 7.4 Hz, 1H), 6.96 (bs, 1H), 3.07 (t, J = 7.6 Hz, 2H), 2.67 (t, J = 7.6 Hz, 2H) ppm.

^{13}C NMR (101 MHz, $CDCl_3$) δ: 170.49, 140.82, 137.88, 129.18, 128.86, 128.61, 126.61, 124.51, 120.07, 39.74, 31.77 ppm.

IR: ν 3325, 3030, 2930, 2861, 1652, 1600, 1528, 1441 cm^{-1}.

HRMS (ESI-TOF) *m/z*: $[M+H^+]$ calculated for $C_{15}H_{16}NO$, 226.1232; found, 226.1236.

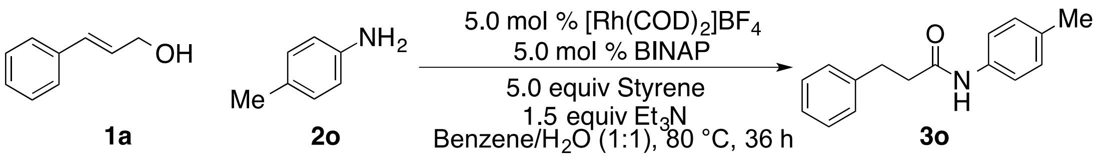

3-phenyl-*N*-(*p*-tolyl)propanamide (Table 1, 3o): $[Rh(COD)_2]BF_4$ (5.0 mg, 0.0125 mmol, 5.0 mol %) and BINAP (7.8 mg, 0.0125 mmol, 5.0 mol %) were added to a 4 mL vial equipped with a stir bar. After purging with with nitrogen, to the vial was added sequentially benzene (0.2 mL), *p*-toluidine (81 mg, 0.75 mmol, 3.0 equiv), styrene (145 µL, 1.25 mmol, 5.0 equiv), triethylamine (53 µL, 0.38 mmol, 1.5 equiv), cinnamyl alcohol (32 µL, 0.25 mmol, 1.0 equiv) and DI water (0.2 mL). The resulting solution was allowed to stir for 36 h at 80 °C. Then, the reaction vial was cooled to room temperature followed by the addition of diphenylmethane as an internal standard for analysis of the crude reaction mixture. The biphasic solution was added EtOAc, extracted with 1 M HCl three times. The combined organic layer was dried over anhydrous $MgSO_4$, filtered and concentrated *in vacuo* and then purified by silica gel chromatography (10:1 hexanes/EtOAc) to afford a white solid in 64% yield.

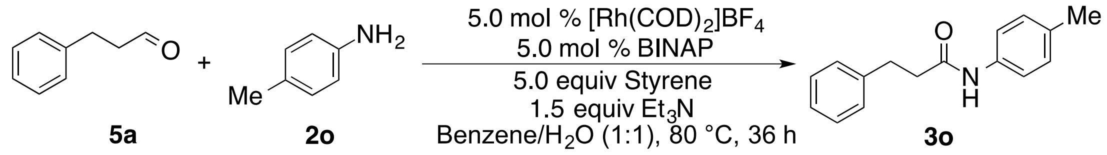

3-phenyl-*N*-(*p*-tolyl)propanamide (Table 1, 3o): $[Rh(COD)_2]BF_4$ (5.0 mg, 0.0125 mmol, 5.0 mol %) and BINAP (7.8 mg, 0.0125 mmol, 5.0 mol %) were added to a 4 mL vial equipped with a stir bar. After purging with with nitrogen, to the vial was added sequentially benzene (0.2 mL), *p*-toluidine (81 mg, 0.75 mmol, 3.0 equiv), styrene (145 µL, 1.25 mmol, 5.0 equiv), triethylamine (53 µL, 0.38 mmol, 1.5 equiv), 3-phenyl-propanal (34 µL, 0.25 mmol, 1.0 equiv) and DI water (0.2 mL). The resulting solution was allowed to stir for 36 h at 80 °C. Then, the reaction vial was cooled to room temperature followed by the addition of diphenylmethane as an internal standard for analysis of the crude reaction mixture. The biphasic solution was added EtOAc, extracted with 1 M HCl three times. The combined organic layer was dried over anhydrous $MgSO_4$, filtered and concentrated *in vacuo* and then purified by silica gel chromatography (10:1 hexanes/EtOAc) to afford a white solid in 53% yield.

$\mathbf{R_f}$ = 0.1

mp =126-129 °C (reported mp =129-130 °C)[6]

^{1}H NMR (500 MHz, $CDCl_3$) δ: 7.33 – 7.28 (m, 4H), 7.26 – 7.20 (m, 3H), 7.10 (d, J = 8.0 Hz, 2H), 6.93 (bs, 1H), 3.06 (t, J = 7.6 Hz, 2H), 2.65 (t, J = 7.6 Hz, 2H), 2.30 (s, 3H) ppm.

^{13}C NMR (126 MHz, $CDCl_3$) δ: 170.38, 140.83, 135.28, 134.06, 129.57, 128.76, 128.53, 126.49, 120.18, 39.57, 31.75, 20.98 ppm.

IR: ν 3320, 3027, 2926, 2867, 1654, 1595, 1564 cm^{-1}.

HRMS (ESI-TOF) *m/z*: $[M+H^+]$ calculated for $C_{16}H_{18}NO$, 240.1388; found, 240.1391.

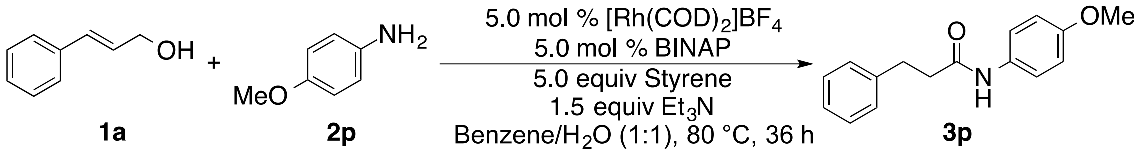

***N*-(4-methoxyphenyl)-3-phenylpropanamide (Table 1, 3p):** $[Rh(COD)_2]BF_4$ (5.0 mg, 0.0125 mmol, 5.0 mol %) and BINAP (7.8 mg, 0.0125 mmol, 5.0 mol %) were added to a 4 mL vial equipped with a stir bar. After purging with with nitrogen, to the vial was added sequentially benzene (0.2 mL), 4-methoxyaniline (92 mg, 0.75 mmol, 3.0 equiv), styrene (145 µL, 1.25 mmol, 5.0 equiv), triethylamine (53 µL, 0.38 mmol, 1.5 equiv), cinnamyl alcohol (32 µL, 0.25 mmol, 1.0 equiv) and DI water (0.2 mL). The resulting solution was allowed to stir for 36 h at 80 °C. Then, the reaction vial was cooled to room temperature followed by the addition of diphenylmethane as an internal standard for analysis of the crude reaction mixture. The biphasic solution was added EtOAc, extracted with 1 M HCl three times. The combined organic layer was dried over anhydrous $MgSO_4$, filtered and concentrated *in vacuo* and then purified by silica gel chromatography (10:1 hexanes/EtOAc) to afford a white solid in 53% yield.

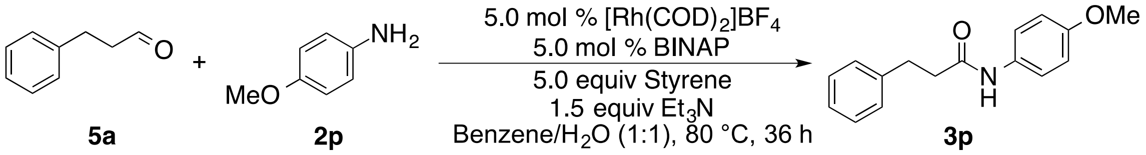

***N*-(4-methoxyphenyl)-3-phenylpropanamide (Table 1, 3p):** $[Rh(COD)_2]BF_4$ (5.0 mg, 0.0125 mmol, 5.0 mol %) and BINAP (7.8 mg, 0.0125 mmol, 5.0 mol %) were added to a 4 mL vial equipped with a stir bar. After purging with with nitrogen, to the vial was added sequentially benzene (0.2 mL), 4-methoxyaniline (92 mg, 0.75 mmol, 3.0 equiv), styrene (145 µL, 1.25 mmol, 5.0 equiv), triethylamine (53 µL, 0.38 mmol, 1.5 equiv), 3-phenyl-propanal (34 µL, 0.25 mmol, 1.0 equiv) and DI water (0.2 mL). The resulting solution was allowed to stir for 36 h at 80 °C. Then, the reaction vial was cooled to room temperature followed by the addition of diphenylmethane as an internal standard for analysis of the crude reaction mixture. The biphasic solution was added EtOAc, extracted with 1 M HCl three times. The combined organic layer was dried over anhydrous $MgSO_4$, filtered and concentrated *in vacuo* and then purified by silica gel chromatography (10:1 hexanes/EtOAc) to afford a white solid in 59% yield.

R_f = 0.15

mp =127-130 °C (reported mp =130-131 °C)[7]

^{1}H NMR (500 MHz, $CDCl_3$) δ: 7.35 – 7.28 (m, 4H), 7.26 – 7.20 (m, 3H), 6.88 (bs, 1H), 6.86 – 6.80 (m, 2H), 3.78 (s, 3H), 3.05 (t, J = 7.6 Hz, 2H), 2.64 (t, J = 7.6 Hz, 2H) ppm.

^{13}C NMR (126 MHz, $CDCl_3$) δ: 170.36, 156.58, 140.84, 130.91, 128.76, 128.54, 126.50, 122.05, 114.25, 55.61, 39.46, 31.81 ppm.

IR: ν 3288, 2998, 2948, 2845, 1651, 1606, 1508 cm^{-1}.

HRMS (ESI-TOF) *m/z*: $[M+H^+]$ calculated for $C_{16}H_{18}NO_2$, 256.1338; found, 256.1340.

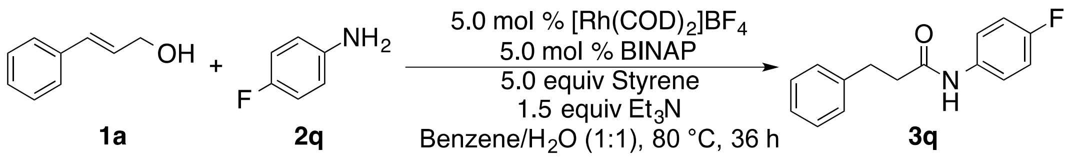

***N*-(4-fluorophenyl)-3-phenylpropanamide (Table 1, 3q):** $[Rh(COD)_2]BF_4$ (5.0 mg, 0.0125 mmol, 5.0 mol %) and BINAP (7.8 mg, 0.0125 mmol, 5.0 mol %) were added to a 4 mL vial equipped with a stir bar. After purging with with nitrogen, to the vial was added sequentially benzene (0.2 mL), 4-fluoroaniline (71 µL, 0.75 mmol, 3.0 equiv), styrene (145 µL, 1.25 mmol, 5.0 equiv), triethylamine (53 µL, 0.38 mmol, 1.5 equiv), cinnamyl alcohol (32 µL, 0.25 mmol, 1.0 equiv) and DI water (0.2 mL). The resulting solution was allowed to stir for 36 h at 80 °C. Then, the reaction vial was cooled to room temperature followed by the addition of diphenylmethane as an internal standard for analysis of the crude reaction mixture. The biphasic solution was added EtOAc, extracted with 1 M HCl three times. The combined organic layer was dried over anhydrous $MgSO_4$, filtered and concentrated *in vacuo* and then purified by silica gel chromatography (10:1 hexanes/ EtOAc) to afford a white solid in 66% yield.

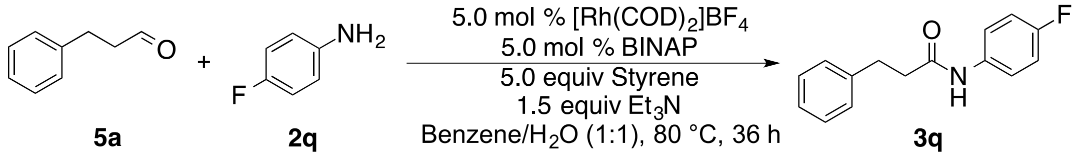

***N*-(4-fluorophenyl)-3-phenylpropanamide (Table 1, 3q):** $[Rh(COD)_2]BF_4$ (5.0 mg, 0.0125 mmol, 5.0 mol %) and BINAP (7.8 mg, 0.0125 mmol, 5.0 mol %) were added to a 4 mL vial equipped with a stir bar. After purging with with nitrogen, to the vial was added sequentially benzene (0.2 mL), 4-fluoroaniline (71 µL, 0.75 mmol, 3.0 equiv), styrene (145 µL, 1.25 mmol, 5.0 equiv), triethylamine (53 µL, 0.38 mmol, 1.5 equiv), 3-phenyl-propanal (34 µL, 0.25 mmol, 1.0 equiv) and DI water (0.2 mL). The resulting solution was allowed to stir for 36 h at 80 °C. Then, the reaction vial was cooled to room temperature followed by the addition of diphenylmethane as an internal standard for analysis of the crude reaction mixture. The biphasic solution was added EtOAc, extracted with 1 M HCl three times. The combined organic layer was dried over anhydrous $MgSO_4$, filtered and concentrated *in vacuo* and then purified by silica gel chromatography (10:1 hexanes/ EtOAc) to afford a white solid in 56% yield.

R_f = 0.2 **mp** =116-118 °C (reported mp =116-118 °C)[8]

^{1}H NMR (400 MHz, $CDCl_3$) δ: 7.42 – 7.34 (m, 2H), 7.34 – 7.27 (m, 1H), 7.27 – 7.19 (m, 3H), 7.07 – 6.86 (m, 3H), 3.05 (t, J = 7.6 Hz, 2H), 2.65 (t, J = 7.6 Hz, 2H) ppm.

^{13}C NMR (126 MHz, $CDCl_3$) δ: 170.47, 159.78 (d, $^1J_{CF}$ = 242.5 Hz), 140.66, 133.76, 128.81, 128.53, 126.59, 121.94 (d, $^3J_{CF}$= 8.8 Hz), 115.72 (d, $^2J_{CF}$ = 21.8 Hz), 39.48, 31.70 ppm.

^{19}F NMR (470 MHz, $CDCl_3$) δ: -118.98 (t, $^2J_{FH}$ = 7.8 Hz) ppm.

IR: ν 3293, 3072, 3036, 2958, 1652, 1507 cm^{-1}.

HRMS (ESI-TOF) *m/z*: [M+H$^+$] calculated for $C_{15}H_{15}NOF$, 244.1138; found, 244.1143.

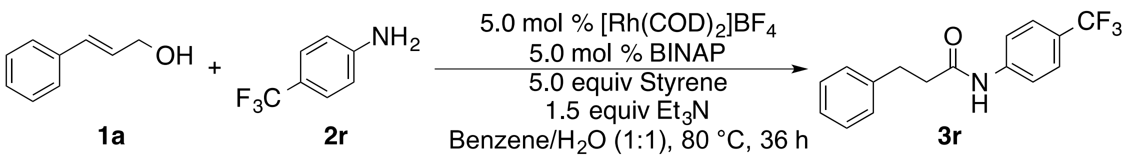

3-phenyl-*N*-(4-(trifluoromethyl)phenyl)propanamide (Table 1, 3r): $[Rh(COD)_2]BF_4$ (5.0 mg, 0.0125 mmol, 5.0 mol %) and BINAP (7.8 mg, 0.0125 mmol, 5.0 mol %) were added to a 4 mL vial equipped with a stir bar. After purging with with nitrogen, to the vial was added sequentially benzene (0.2 mL), 4-(trifluoromethyl)aniline (94 µL, 0.75 mmol, 3.0 equiv), styrene (145 µL, 1.25 mmol, 5.0 equiv), triethylamine (53 µL, 0.38 mmol, 1.5 equiv), cinnamyl alcohol (32 µL, 0.25 mmol, 1.0 equiv) and DI water (0.2 mL). The resulting solution was allowed to stir for 36 h at 80 °C. Then, the reaction vial was cooled to room temperature followed by the addition of diphenylmethane as an internal standard for analysis of the crude reaction mixture. The biphasic solution was added EtOAc, extracted with 1 M HCl three times. The combined organic layer was dried over anhydrous $MgSO_4$, filtered and concentrated *in vacuo* and then purified by silica gel chromatography (10:1 hexanes/EtOAc) to afford a white solid in 41% yield.

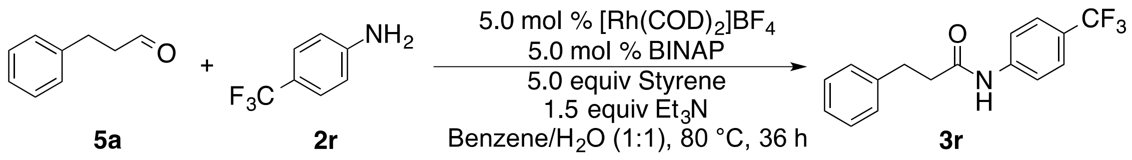

3-phenyl-*N*-(4-(trifluoromethyl)phenyl)propanamide (Table 1, 3r): $[Rh(COD)_2]BF_4$ (5.0 mg, 0.0125 mmol, 5.0 mol %) and BINAP (7.8 mg, 0.0125 mmol, 5.0 mol %) were added to a 4 mL vial equipped with a stir bar. After purging with with nitrogen, to the vial was added sequentially benzene (0.2 mL), 4-(trifluoromethyl)aniline (94 µL, 0.75 mmol, 3.0 equiv), styrene (145 µL, 1.25 mmol, 5.0 equiv), triethylamine (53 µL, 0.38 mmol, 1.5 equiv), 3-phenyl-propanal (34 µL, 0.25 mmol, 1.0 equiv) and DI water (0.2 mL). The resulting solution was allowed to stir for 36 h at 80 °C. Then, the reaction vial was cooled to room temperature followed by the addition of diphenylmethane as an internal standard for analysis of the crude reaction mixture. The biphasic solution was added EtOAc, extracted with 1 M HCl three times. The combined organic layer was dried over anhydrous $MgSO_4$, filtered and concentrated *in vacuo* and then purified by silica gel chromatography (10:1 hexanes/EtOAc) to afford a white solid in 39% yield.

R_f = 0.1 **mp** =147-150 °C

^{1}H NMR (500 MHz, $CDCl_3$) δ: 7.55 (s, 4H), 7.36 – 7.28 (m, 2H), 7.26 – 7.20 (m, 3H), 7.08 (bs, 1H), 3.07 (t, J = 7.5 Hz, 2H), 2.70 (t, J = 7.6 Hz, 2H) ppm.

^{13}C NMR (126 MHz, $CDCl_3$) δ: 170.75, 140.82, 140.44, 128.88, 128.51, 126.69, 126.38 (q, $^3J_{CF}$ = 3.8 Hz), 125.92 (q, $^2J_{CF}$ = 31.2 Hz), 124.17 (q, $^1J_{CF}$ = 270.0 Hz), 119.48, 39.66, 31.55 ppm.

^{19}F NMR (470 MHz, $CDCl_3$) δ: -62.20 (s) ppm.

IR: ν 3329, 3031, 2931, 1673, 1600, 1524 cm^{-1}.

HRMS (ESI-TOF) *m/z*: [M+H$^+$] calculated for $C_{16}H_{15}NOF_3$, 294.1106; found, 294.1106.

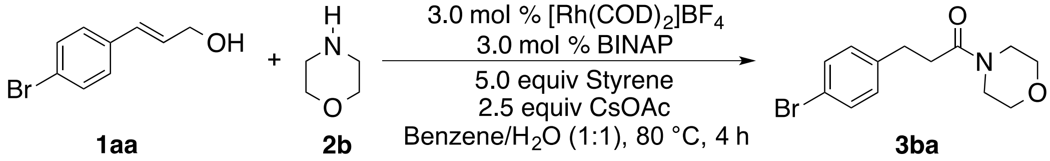

3-(4-bromophenyl)-1-morpholinopropan-1-one (Table 2, 3ba): $[Rh(COD)_2]BF_4$ (3.1 mg, 0.0075 mmol, 3.0 mol %), BINAP (4.7 mg, 0.0075 mmol, 3.0 mol %), and CsOAc (120 mg, 1.25 mmol, 2.5 equiv) were added to a 4 mL vial equipped with a stir bar. After purging with with nitrogen, to the vial was added sequentially benzene (0.2 mL), morpholine (65 µL, 0.75 mmol, 3.0 equiv), styrene (145 µL, 1.25 mmol, 5.0 equiv), (*E*)-3-(4-bromophenyl)prop-2-en-1-ol (53 mg, 0.25 mmol, 1.0 equiv) and DI water (0.2 mL). The resulting solution was allowed to stir for 4 h at 80 °C. Then, the reaction vial was cooled to room temperature followed by the addition of diphenylmethane as an internal standard for analysis of the crude reaction mixture. The biphasic solution was added EtOAc, dried over anhydrous $MgSO_4$, and filtered. The solution was concentrated *in vacuo* and then purified by silica gel chromatography (1:1 hexanes/EtOAc) to afford a white solid in 76% yield.

R_f = 0.1

mp = 69-71°C

^{1}H NMR (500 MHz, $CDCl_3$) δ: 7.46 – 7.36 (m, 2H), 7.11 – 7.05 (m, 2H), 3.71 – 3.59 (m, 4H), 3.57 (t, J = 4.9 Hz, 2H), 3.44 – 3.32 (m, 2H), 2.94 (t, J = 7.7 Hz, 2H), 2.58 (t, J = 7.7 Hz, 2H).

^{13}C NMR (126 MHz, $CDCl_3$) δ: 170.56, 140.25, 131.71, 130.39, 120.14, 67.02, 66.64, 46.03, 42.11, 34.67, 30.81.

IR: ν 2973, 2920, 2850, 1632.

HRMS (ESI-TOF) *m/z*: $[M+H^+]$ calculated for $C_{13}H_{17}NOBr$, 298.0443; found, 298.0455.

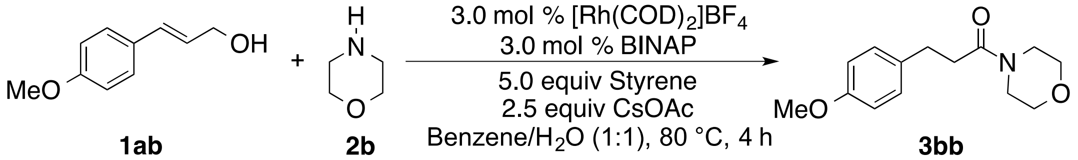

3-(4-methoxyphenyl)-1-morpholinopropan-1-one (Table 2, 3bb): $[Rh(COD)_2]BF_4$ (3.1 mg, 0.0075 mmol, 3.0 mol %), BINAP (4.7 mg, 0.0075 mmol, 3.0 mol %), and CsOAc (120 mg, 1.25 mmol, 2.5 equiv) were added to a 4 mL vial equipped with a stir bar. After purging with with nitrogen, to the vial was added sequentially benzene (0.2 mL), morpholine (65 µL, 0.75 mmol, 3.0 equiv), styrene (145 µL, 1.25 mmol, 5.0 equiv), (*E*)-3-(4-methoxyphenyl)prop-2-en-1-ol (41 mg, 0.25 mmol, 1.0 equiv) and DI water (0.2 mL). The resulting solution was allowed to stir for 4 h at 80 °C. Then, the reaction vial was cooled to room temperature followed by the addition of diphenylmethane as an internal standard for analysis of the crude reaction mixture. The biphasic solution was added EtOAc, dried over anhydrous $MgSO_4$, and filtered. The solution was concentrated *in vacuo* and then purified by silica gel chromatography (2:1 hexanes/EtOAc) to afford a colorless liquid in 72% yield.

R_f = 0.1

^{1}H NMR (400 MHz, $CDCl_3$) δ: 7.18 – 7.08 (m, 2H), 6.89 – 6.76 (m, 2H), 3.78 (s, 3H), 3.74 – 3.56 (m, 4H), 3.67 – 3.46 (m, 2H), 3.42 – 3.30 (m, 2H), 2.91 (t, J = 7.7 Hz, 2H), 2.58 (t, J = 7.8 Hz, 2H).

^{13}C NMR (101 MHz, $CDCl_3$) δ: 171.08, 158.18, 133.19, 129.51, 114.03, 66.99, 66.62, 55.14, 46.09, 42.03, 35.20, 30.71.

IR: ν 2963, 2929, 2860, 1639, 1512.

HRMS (ESI-TOF) *m/z*: [M+H$^+$] calculated for $C_{14}H_{20}NO_3$, 250.1443; found, 250.1455.

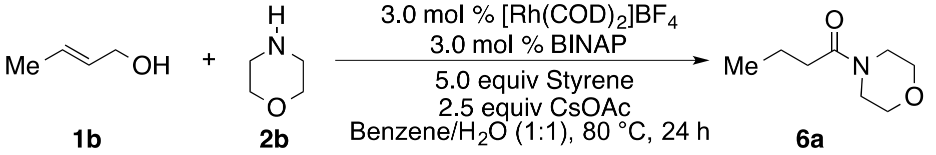

1-morpholinobutan-1-one (Table 2, 6a): $[Rh(COD)_2]BF_4$ (3.1 mg, 0.0075 mmol, 3.0 mol %), BINAP (4.7 mg, 0.0075 mmol, 3.0 mol %), and CsOAc (120 mg, 1.25 mmol, 2.5 equiv) were added to a 4 mL vial equipped with a stir bar. After purging with with nitrogen, to the vial was added sequentially benzene (0.2 mL), morpholine (65 µL, 0.75 mmol, 3.0 equiv), styrene (145 µL, 1.25 mmol, 5.0 equiv), crotyl alcohol (22 µL, 0.25 mmol, 1.0 equiv) and DI water (0.2 mL). The resulting solution was allowed to stir for 24 h at 80 °C. Then, the reaction vial was cooled to room temperature followed by the addition of diphenylmethane as an internal standard for analysis of the crude reaction mixture. The biphasic solution was added EtOAc, dried over anhydrous $MgSO_4$, and filtered. The solution was concentrated *in vacuo* and then purified by silica gel chromatography (1:1 hexanes/EtOAc) to afford a light yellow liquid in 77% yield.

R_f = 0.2

^{1}H NMR (500 MHz, $CDCl_3$) δ: 3.66 (dd, J = 5.7, 3.9 Hz, 4H), 3.63 – 3.43 (m, 4H), 2.29 (t, J = 7.6 Hz, 2H), 1.66 (h, J = 7.4 Hz, 2H), 0.97 (t, J = 7.4 Hz, 3H).

^{13}C NMR (126 MHz, $CDCl_3$) δ: 171.82, 67.05, 66.84, 46.15, 41.96, 35.15, 18.79, 14.11.

IR: ν 2965, 2934, 2861, 1642, 1433.

HRMS (ESI-TOF) *m/z*: [M+H$^+$] calculated for $C_8H_{16}NO_2$, 158.1181; found, 158.1180.

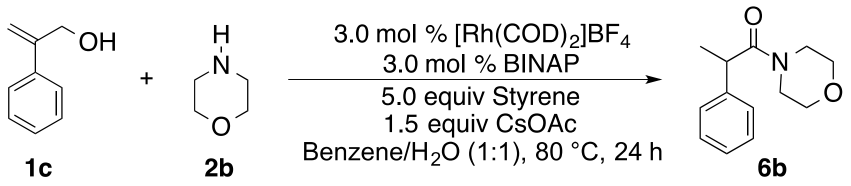

1-morpholino-2-phenylpropan-1-one (Table 2, 6b): $[Rh(COD)_2]BF_4$ (3.1 mg, 0.0075 mmol, 3.0 mol %), BINAP (4.7 mg, 0.0075 mmol, 3.0 mol %), and CsOAc (72 mg, 0.38 mmol, 1.5 equiv) were added to a 4 mL vial equipped with a stir bar. After purging with with nitrogen, to the vial was added sequentially benzene (0.2 mL), morpholine (65 µL, 0.75 mmol, 3.0 equiv), styrene (145 µL, 1.25 mmol, 5.0 equiv), 2-phenylprop-2-en-1-ol (32 µL, 0.25 mmol, 1.0 equiv) and DI water (0.2 mL). The resulting solution was allowed to stir for 24 h at 80 °C. Then, the reaction vial was cooled to room temperature followed by the addition of diphenylmethane as an internal standard for analysis of the crude reaction mixture. The biphasic solution was added EtOAc, dried over anhydrous $MgSO_4$, and filtered. The solution was concentrated *in vacuo* and then purified by silica gel chromatography (1:1 hexanes/EtOAc) to afford a light yellow liquid in 61% yield.

R_f = 0.2

^{1}H NMR (400 MHz, $CDCl_3$) δ: 7.35 – 7.28 (m, 2H), 7.26 – 7.20 (m, 3H), 3.83 (q, J = 6.9 Hz, 1H), 3.78 (dd, J = 9.4, 3.6 Hz, 1H), 3.66 (dd, J = 9.4, 4.6 Hz, 1H), 3.57 – 3.43 (m, 3H), 3.39 (ddd, J = 13.5, 7.4, 3.2 Hz, 1H), 3.29 (ddd, J = 13.9, 6.1, 3.4 Hz, 1H), 3.09 (ddd, J = 10.8, 7.3, 3.1 Hz, 1H), 1.45 (d, J = 6.8 Hz, 3H).

^{13}C NMR (101 MHz, $CDCl_3$) δ: 172.28, 141.93, 129.12, 127.25, 127.01, 66.90, 66.40, 46.11, 43.37, 42.48, 20.76.

IR: ν 2974, 2932, 2858, 1642, 1430.

HRMS (ESI-TOF) *m/z*: [M+H$^+$] calculated for $C_{13}H_{18}NO_2$, 220.1338; found, 220.1340.

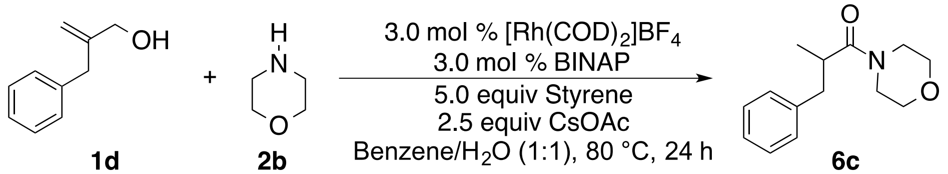

1d **2b** **6c**

2-methyl-1-morpholino-3-phenylpropan-1-one (Table 2, 6c): $[Rh(COD)_2]BF_4$ (3.1 mg, 0.0075 mmol, 3.0 mol %), BINAP (4.7 mg, 0.0075 mmol, 3.0 mol %), and CsOAc (120 mg, 1.25 mmol, 2.5 equiv) were added to a 4 mL vial equipped with a stir bar. After purging with with nitrogen, to the vial was added sequentially benzene (0.2 mL), morpholine (65 µL, 0.75 mmol, 3.0 equiv), styrene (145 µL, 1.25 mmol, 5.0 equiv), 2-benzylprop-2-en-1-ol (37 µL, 0.25 mmol, 1.0 equiv) and DI water (0.2 mL). The resulting solution was allowed to stir for 24 h at 80 °C. Then, the reaction vial was cooled to room temperature followed by the addition of diphenylmethane as an internal standard for analysis of the crude reaction mixture. The biphasic solution was added EtOAc, dried over anhydrous $MgSO_4$, and filtered. The solution was concentrated *in vacuo* and then purified by silica gel chromatography (1:1 hexanes/ EtOAc) to afford a light yellow liquid in 61% yield.

R_f = 0.35

^{1}H NMR (500 MHz, $CDCl_3$) δ: 7.32 – 7.26 (m, 2H), 7.24 – 7.13 (m, 3H), 3.74 – 3.54 (m, 2H), 3.52 – 3.38 (m, 3H), 3.30 (ddd, J = 13.5, 7.5, 3.2 Hz, 1H), 3.15 (ddd, J = 13.4, 5.9, 3.0 Hz, 1H), 3.01 (ddd, J = 11.9, 7.9, 3.6 Hz, 1H), 2.98 – 2.93 (m, 2H), 2.78 – 2.62 (m, 1H), 1.18 (d, J = 6.0 Hz, 3H).

^{13}C NMR (126 MHz, $CDCl_3$) δ: 174.55, 140.02, 129.18, 128.55, 126.53, 66.94, 66.52, 46.11, 42.19, 40.94, 37.43, 18.15.

IR: ν 2971, 2929, 2859, 1636, 1433.

HRMS (ESI-TOF) *m/z*: [M+H$^+$] calculated for $C_{14}H_{20}NO_2$, 234.1494; found, 234.1493.

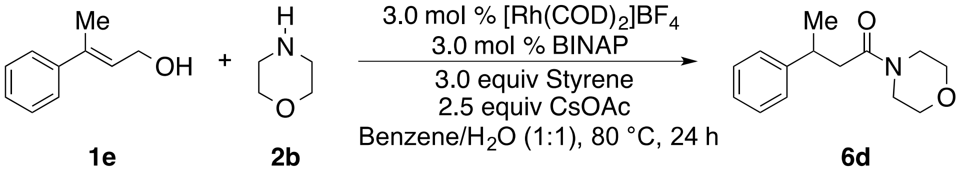

1e **2b** **6d**

1-morpholino-3-phenylbutan-1-one (Table 2, 6d): $[Rh(COD)_2]BF_4$ (3.1 mg, 0.0075 mmol, 3.0 mol %), BINAP (4.7 mg, 0.0075 mmol, 3.0 mol %), and CsOAc (120 mg, 1.25 mmol, 2.5 equiv) were added to a 4 mL vial equipped with a stir bar. After purging with with nitrogen, to the vial was added sequentially benzene (0.2 mL), morpholine (65 µL, 0.75 mmol, 3.0 equiv), styrene (87 µL, 0.75 mmol, 3.0 equiv), (*E*)-3-phenylbut-2-en-1-ol (38 µL, 0.25 mmol, 1.0 equiv) and DI water (0.2

mL). The resulting solution was allowed to stir for 24 h at 80 °C. Then, the reaction vial was cooled to room temperature followed by the addition of diphenylmethane as an internal standard for analysis of the crude reaction mixture. The biphasic solution was added EtOAc, dried over anhydrous $MgSO_4$, and filtered. The solution was concentrated *in vacuo* and then purified by silica gel chromatography (1:1 hexanes/ EtOAc) to afford a colorless liquid in 84% yield.

R_f = 0.15

^{1}H NMR (500 MHz, $CDCl_3$) δ: 7.32 – 7.27 (m, 2H), 7.25 – 7.18 (m, 3H), 3.71 – 3.58 (m, 2H), 3.56 – 3.42 (m, 3H), 3.39 – 3.28 (m, 2H), 3.28 – 3.16 (m, 2H), 2.62 (dd, J = 14.5, 7.0 Hz, 1H), 2.50 (dd, J = 14.5, 7.4 Hz, 1H), 1.35 (d, J = 7.0 Hz, 3H).

^{13}C NMR (126 MHz, $CDCl_3$) δ: 170.55, 146.14, 128.67, 127.04, 126.62, 66.96, 66.55, 46.33, 42.02, 41.54, 37.06, 21.77.

IR: ν 2966, 2926, 2860, 1638, 1455, 1429.

HRMS (ESI-TOF) *m/z*: [M+H$^+$] calculated for $C_{14}H_{20}NO_2$, 234.1494; found, 234.1499.

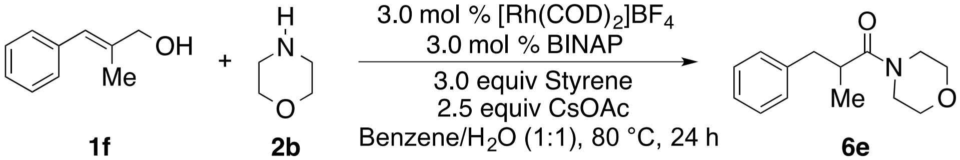

1f 2b 6e

2-methyl-1-morpholino-3-phenylpropan-1-one (Table 2, 6e): $[Rh(COD)_2]BF_4$ (3.1 mg, 0.0075 mmol, 3.0 mol %), BINAP (4.7 mg, 0.0075 mmol, 3.0 mol %), and CsOAc (120 mg, 1.25 mmol, 2.5 equiv) were added to a 4 mL vial equipped with a stir bar. After purging with with nitrogen, to the vial was added sequentially benzene (0.2 mL), morpholine (65 µL, 0.75 mmol, 3.0 equiv), styrene (87 µL, 0.75 mmol, 3.0 equiv), (*E*)-2-methyl-3-phenylprop-2-en-1-ol (36 µL, 0.25 mmol, 1.0 equiv) and DI water (0.2 mL). The resulting solution was allowed to stir for 24 h at 80 °C. Then, the reaction vial was cooled to room temperature followed by the addition of diphenylmethane as an internal standard for analysis of the crude reaction mixture. The biphasic solution was added EtOAc, dried over anhydrous $MgSO_4$, and filtered. The solution was concentrated *in vacuo* and then purified by silica gel chromatography (1:1 hexanes/ EtOAc) to afford a colorless liquid in 52% yield.

R_f = 0.1

^{1}H NMR (400 MHz, $CDCl_3$) δ: 7.32 – 7.25 (m, 2H), 7.24 – 7.14 (m, 3H), 3.74 – 3.55 (m, 2H), 3.51 – 3.38 (m, 3H), 3.35 – 3.25 (m, 1H), 3.21 – 3.10 (m, 1H), 3.07 – 2.86 (m, 3H), 2.76 – 2.65 (m, 1H), 1.18 (d, J = 6.0 Hz, 3H).

^{13}C NMR (126 MHz, $CDCl_3$) δ: 174.55, 139.99, 129.16, 128.53, 126.52, 66.92, 66.50, 46.10, 42.17, 40.93, 37.41, 18.14.

IR: ν 2972, 2928, 2859, 1635, 1434.

HRMS (ESI-TOF) *m/z*: [M+H$^+$] calculated for $C_{14}H_{20}NO_2$, 234.1494; found, 234.1496.

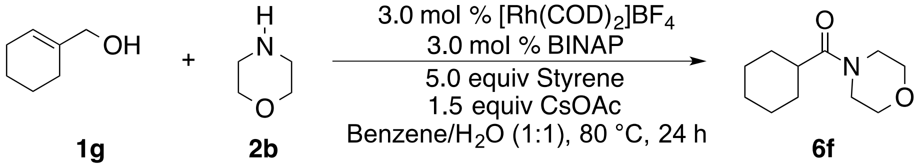

1g 2b 6f

cyclohexyl(morpholino)methanone (Table 2, 6f): $[Rh(COD)_2]BF_4$ (3.1 mg, 0.0075 mmol, 3.0 mol %), BINAP (4.7 mg, 0.0075 mmol, 3.0 mol %), and CsOAc (72 mg, 0.38 mmol, 1.5 equiv) were added to a 4 mL vial equipped with a stir bar.

After purging with with nitrogen, to the vial was added sequentially benzene (0.2 mL), morpholine (65 µL, 0.75 mmol, 3.0 equiv), styrene (145 µL, 1.25 mmol, 5.0 equiv), cyclohex-1-en-1-ylmethanol (29 µL, 0.25 mmol, 1.0 equiv) and DI water (0.2 mL). The resulting solution was allowed to stir for 24 h at 80 °C. Then, the reaction vial was cooled to room temperature followed by the addition of diphenylmethane as an internal standard for analysis of the crude reaction mixture. The biphasic solution was added EtOAc, dried over anhydrous $MgSO_4$, and filtered. The solution was concentrated *in vacuo* and then purified by silica gel chromatography (1:1 hexanes/EtOAc) to afford a white solid in 54% yield.

R_f = 0.2

mp = 54-55 °C

^{1}H NMR (400 MHz, $CDCl_3$) δ: 3.73 – 3.63 (m, 4H), 3.64 – 3.41 (m, 4H), 2.43 (tt, J = 11.6, 3.4 Hz, 1H), 1.91 – 1.76 (m, 2H), 1.76 – 1.60 (m, 3H), 1.60 – 1.46 (m, 2H), 1.33 – 1.21 (m, 3H).

^{13}C NMR (101 MHz, $CDCl_3$) δ: 174.90, 67.16, 67.03, 46.04, 42.06, 40.40, 29.45, 25.96, 25.93.

IR: ν 2923, 2852, 1642, 1454, 1452.

HRMS (ESI-TOF) *m/z*: [M+H$^+$] calculated for $C_{11}H_{20}NO_2$, 198.1494; found, 198.1495.

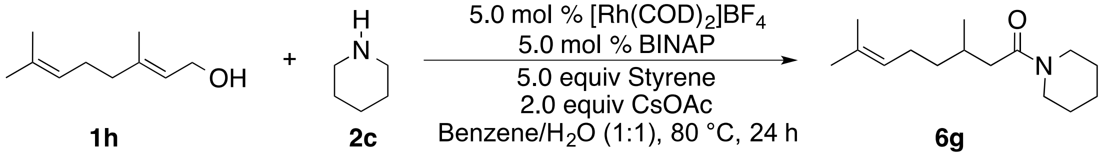

3,7-dimethyl-1-(piperidin-1-yl)oct-6-en-1-one (Table 2, 6g): $[Rh(COD)_2]BF_4$ (5.0 mg, 0.0125 mmol, 5.0 mol %) and BINAP (7.8 mg, 0.0125 mmol, 5.0 mol %), and CsOAc (96 mg, 0.50 mmol, 2.0 equiv) were added to a 4 mL vial equipped with a stir bar. After purging with with nitrogen, to the vial was added sequentially benzene (0.2 mL), piperidine (74 µL, 0.75 mmol, 3.0 equiv), styrene (145 µL, 1.25 mmol, 5.0 equiv), geraniol (44 µL, 0.25 mmol, 1.0 equiv) and DI water (0.2 mL). The resulting solution was allowed to stir for 24 h at 80 °C. Then, the reaction vial was cooled to room temperature followed by the addition of diphenylmethane as an internal standard for analysis of the crude reaction mixture. The biphasic solution was added EtOAc, dried over anhydrous $MgSO_4$, and filtered. The solution was concentrated *in vacuo* and then purified by silica gel chromatography (3:1 hexanes/EtOAc) to afford a light yellow liquid in 64% yield.

R_f = 0.35

^{1}H NMR (400 MHz, $CDCl_3$) δ: 5.23 – 4.88 (m, 1H), 3.61 – 3.47 (m, 2H), 3.45 – 3.31 (m, 2H), 2.32 (dd, J = 14.3, 5.9 Hz, 1H), 2.12 (dd, J = 14.3, 8.4 Hz, 1H), 2.08 – 1.90 (m, 3H), 1.67 (d, J = 1.3 Hz, 3H), 1.65 – 1.61 (m, 1H), 1.60 (d, J = 1.3 Hz, 3H), 1.57 – 1.48 (m, 4H), 1.38 (dddd, J = 13.4, 9.3, 6.5, 5.4 Hz, 1H), 1.21 (dddd, J = 13.6, 9.3, 7.9, 5.8 Hz, 1H), 0.94 (d, J = 6.5 Hz, 3H).

^{13}C NMR (101 MHz, $CDCl_3$) δ: 171.07, 131.51, 124.65, 47.09, 42.79, 40.85, 37.27, 30.26, 29.83, 26.76, 25.83, 25.66, 24.75, 19.95, 17.82.

IR: ν 2929, 2856, 1642, 1443.

HRMS (ESI-TOF) *m/z*: [M+H$^+$] calculated for $C_{15}H_{28}NO$, 238.2171; found, 238.2179.

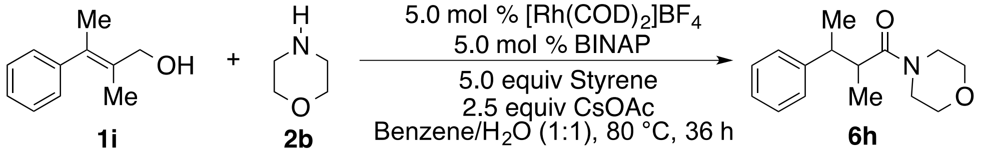

2-methyl-1-morpholino-3-phenylbutan-1-one (Table 2, 6h): $[Rh(COD)_2]BF_4$ (5.0 mg, 0.0125 mmol, 5.0 mol %) and BINAP (7.8 mg, 0.0125 mmol, 5.0 mol %), and CsOAc (120 mg, 0.50 mmol, 2.5 equiv) were added to a 4 mL vial equipped with a stir bar. After purging with with nitrogen, to the vial was added sequentially benzene (0.2 mL), morpholine (65 μL, 0.75 mmol, 3.0 equiv), styrene (145 μL, 1.25 mmol, 5.0 equiv), (*E*)-2-methyl-3-phenylbut-2-en-1-ol (**1i**, 42 μL, 0.25 mmol, 1.0 equiv) and DI water (0.2 mL). The resulting solution was allowed to stir for 36 h at 80 °C. Then, the reaction vial was cooled to room temperature followed by the addition of diphenylmethane as an internal standard for analysis of the crude reaction mixture. The biphasic solution was added EtOAc, dried over anhydrous $MgSO_4$, and filtered. The solution was concentrated *in vacuo* and then purified by silica gel chromatography (1:1 hexanes/EtOAc) to afford two diastereomers in 31% (d. r. = 1.6).

$\mathbf{R_f}$ = 0.2

^{1}H NMR (500 MHz, $CDCl_3$) δ **major diastereomer:** 7.30 – 7.23 (m, 2H), 7.22 – 7.16 (m, 3H), 3.64 – 3.48 (m, 2H), 3.45 – 3.35 (m, 1H), 3.28 – 3.13 (m, 3H), 3.10 – 2.97 (m, 2H), 2.85 – 2.76 (m, 1H), 2.76 – 2.70 (m, 1H), 1.32 (d, J = 7.1 Hz, 2H), 1.21 (d, J = 6.6 Hz, 2H); **minor diastereomer:** 7.34 – 7.28 (m, 2H), 7.25 – 7.17 (m, 3H), 3.72 – 3.67 (m, 4H), 3.66 – 3.61 (m, 2H), 3.59 – 3.51 (m, 2H), 3.04 (dq, J = 9.8, 6.8 Hz, 1H), 2.84 (dq, J = 9.7, 6.7 Hz, 1H), 1.22 (d, J = 6.9 Hz, 3H), 0.89 (d, J = 6.7 Hz, 3H).

^{13}C NMR (126 MHz, $CDCl_3$) δ **major diastereomer:** 174.67, 145.80, 128.48, 127.52, 126.65, 66.85, 66.42, 46.12, 43.01, 42.55, 41.97, 18.06, 16.13; **minor diastereomer**: 174.85, 145.09, 128.61, 127.82, 126.58, 67.25, 66.97, 46.43, 43.74, 42.33, 41.86, 20.57, 17.25.

IR: ν 2967, 2929, 2680, 1621.

HRMS (ESI-TOF) *m/z*: $[M+H^+]$ calculated for $C_{15}H_{22}NO_2$, 248.1651; found, 248.1657.

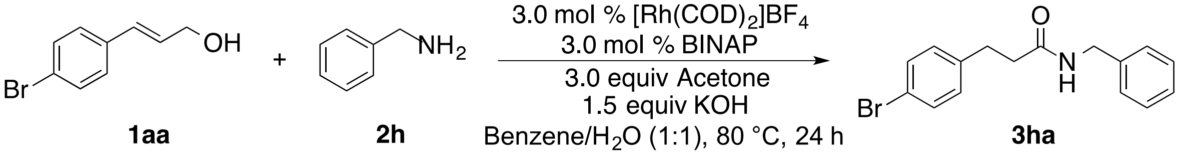

***N*-benzyl-3-(4-bromophenyl)propanamide (Table 2, 3ha):** $[Rh(COD)_2]BF_4$ (3.1 mg, 0.0075 mmol, 3.0 mol %), BINAP (4.7 mg, 0.0075 mmol, 3.0 mol %), and KOH (21 mg, 0.38 mmol, 1.5 equiv) were added to a 4 mL vial equipped with a stir bar. After purging with with nitrogen, to the vial was added sequentially benzene (0.2 mL), benzylamine (82 μL, 0.75 mmol, 3.0 equiv), acetone (54 μL, 0.75 mmol, 3.0 equiv), (*E*)-3-(4-bromophenyl)prop-2-en-1-ol (53 mg, 0.25 mmol, 1.0 equiv) and DI water (0.2 mL). The resulting solution was allowed to stir for 24 h at 80 °C. Then, the reaction vial was cooled to room temperature followed by the addition of diphenylmethane as an internal standard for analysis of the crude reaction mixture. The biphasic solution was added EtOAc, dried over anhydrous $MgSO_4$, and filtered. The solution was concentrated *in vacuo* and then purified by silica gel chromatography (2:1 hexanes/EtOAc) to afford a white solid in 60 % yield.

R_f = 0.1

mp = 136-139 °C (reported mp = 140-141 °C)[1]

^{1}H NMR (500 MHz, $CDCl_3$) δ: 7.43 – 7.36 (m, 2H), 7.34 – 7.26 (m, 3H), 7.15 – 7.11 (m, 2H), 7.10 – 7.04 (m, 2H), 5.59 (bs, 1H), 4.39 (d, J = 5.7 Hz, 2H), 2.95 (t, J = 7.5 Hz, 2H), 2.48 (t, J = 7.4 Hz, 2H).

^{13}C NMR (126 MHz, $CDCl_3$) δ: 171.49, 139.86, 138.14, 131.73, 130.37, 128.84, 127.86, 127.69, 120.20, 43.73, 38.41, 31.18.

IR: ν 3296, 3035, 2934, 1635, 1539.

HRMS (ESI-TOF) *m/z*: [M+H$^+$] calculated for $C_{16}H_{17}NOBr$, 318.0494; found, 318.0491.

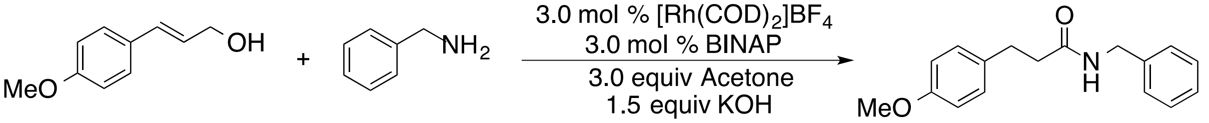

1ab **2h** **3hb**

***N*-benzyl-3-(4-methoxyphenyl)propanamide (Table 2, 3hb):** $[Rh(COD)_2]BF_4$ (3.1 mg, 0.0075 mmol, 3.0 mol %), BINAP (4.7 mg, 0.0075 mmol, 3.0 mol %), and KOH (21 mg, 0.38 mmol, 1.5 equiv) were added to a 4 mL vial equipped with a stir bar. After purging with with nitrogen, to the vial was added sequentially benzene (0.2 mL), benzylamine (82 μL, 0.75 mmol, 3.0 equiv), acetone (54 μL, 0.75 mmol, 3.0 equiv), (*E*)-3-(4-methoxyphenyl)prop-2-en-1-ol (41 mg, 0.25 mmol, 1.0 equiv) and DI water (0.2 mL). The resulting solution was allowed to stir for 24 h at 80 °C. Then, the reaction vial was cooled to room temperature followed by the addition of diphenylmethane as an internal standard for analysis of the crude reaction mixture. The biphasic solution was added EtOAc, dried over anhydrous $MgSO_4$, and filtered. The solution was concentrated *in vacuo* and then purified by silica gel chromatography (2:1 hexanes/EtOAc) to afford a white solid in 70 % yield.

R_f = 0.1

mp = 91-92 °C (reported mp = 92 °C)[17]

^{1}H NMR (500 MHz, $CDCl_3$) δ: 7.35 – 7.24 (m, 3H), 7.19 – 7.04 (m, 4H), 6.87 – 6.72 (m, 2H), 5.58 (bs, 1H), 4.40 (d, J = 5.7 Hz, 2H), 3.79 (s, 3H), 2.94 (t, J = 7.5 Hz, 2H), 2.48 (t, J = 7.5 Hz, 2H).

^{13}C NMR (126 MHz, $CDCl_3$) δ: 172.05, 158.21, 138.29, 132.90, 129.50, 128.78, 127.89, 127.60, 114.08, 55.39, 43.69, 38.98, 31.01.

IR: ν 3292, 3067, 2938, 1635, 1512.

HRMS (ESI-TOF) *m/z*: [M+H$^+$] calculated for $C_{17}H_{20}NO_2$, 270.1494; found, 270.1501.

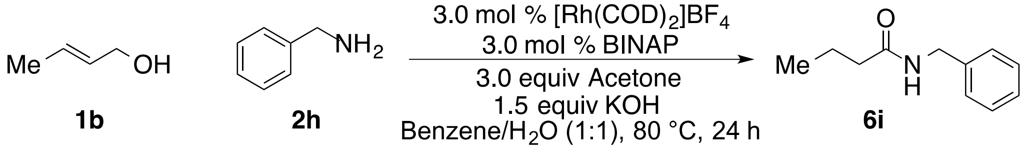

1b **2h** **6i**

***N*-benzylbutyramide (Table 2, 6i):** $[Rh(COD)_2]BF_4$ (3.1 mg, 0.0075 mmol, 3.0 mol %), BINAP (4.7 mg, 0.0075 mmol, 3.0 mol %), and KOH (21 mg, 0.38 mmol, 1.5 equiv) were added to a 4 mL vial equipped with a stir bar. After purging with

with nitrogen, to the vial was added sequentially benzene (0.2 mL), benzylamine (82 μL, 0.75 mmol, 3.0 equiv), acetone (54 μL, 0.75 mmol, 3.0 equiv), crotyl alcohol (22 μL, 0.25 mmol, 1.0 equiv) and DI water (0.2 mL). The resulting solution was allowed to stir for 24 h at 80 °C. Then, the reaction vial was cooled to room temperature followed by the addition of diphenylmethane as an internal standard for analysis of the crude reaction mixture. The biphasic solution was added EtOAc, dried over anhydrous $MgSO_4$, and filtered. The solution was concentrated *in vacuo* and then purified by silica gel chromatography (4:1 hexanes/EtOAc) to afford a white solid in 87% yield.

$\mathbf{R_f}$ = 0.2

mp = 48-50 °C (reported mp = 49-50 °C)[11]

^{1}H NMR (400 MHz, $CDCl_3$) δ: 7.54 – 7.09 (m, 5H), 5.76 (bs, 1H), 4.44 (d, J = 5.7 Hz, 2H), 2.19 (t, J = 7.5 Hz, 2H), 1.69 (h, J = 7.4 Hz, 2H), 0.96 (t, J = 7.4 Hz, 3H).

^{13}C NMR (101 MHz, $CDCl_3$) δ: 172.93, 138.53, 128.83, 127.95, 127.62, 43.70, 38.84, 19.32, 13.94.

IR: ν 3282, 2927, 2853, 1641, 1551.

HRMS (ESI-TOF) *m/z*: [M+H$^+$] calculated for $C_{11}H_{16}NO$, 178.1232; found, 178.1237.

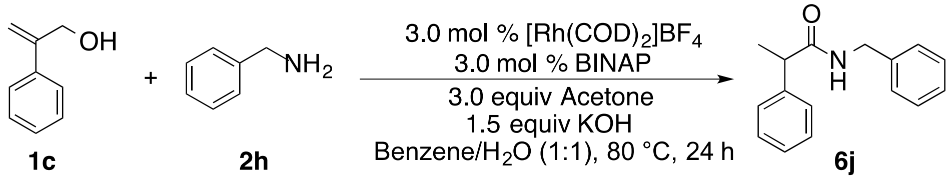

***N*-benzyl-2-phenylpropanamide (Table 2, 6j):** $[Rh(COD)_2]BF_4$ (3.1 mg, 0.0075 mmol, 3.0 mol %), BINAP (4.7 mg, 0.0075 mmol, 3.0 mol %), and KOH (21 mg, 0.38 mmol, 1.5 equiv) were added to a 4 mL vial equipped with a stir bar. After purging with with nitrogen, to the vial was added sequentially benzene (0.2 mL), benzylamine (82 μL, 0.75 mmol, 3.0 equiv), acetone (54 μL, 0.75 mmol, 3.0 equiv), 2-phenylprop-2-en-1-ol (32 μL, 0.25 mmol, 1.0 equiv) and DI water (0.2 mL). The resulting solution was allowed to stir for 24 h at 80 °C. Then, the reaction vial was cooled to room temperature followed by the addition of diphenylmethane as an internal standard for analysis of the crude reaction mixture. The biphasic solution was added EtOAc, dried over anhydrous $MgSO_4$, and filtered. The solution was concentrated *in vacuo* and then purified by silica gel chromatography (4:1 hexanes/EtOAc) to afford an orange solid in 74% yield.

$\mathbf{R_f}$ = 0.25

mp = 74-75 °C (reported mp = 76 °C)[12]

^{1}H NMR (400 MHz, $CDCl_3$) δ: 7.39 – 7.20 (m, 8H), 7.18 – 7.11 (m, 2H), 5.60 (bs, 1H), 4.47 – 4.32 (m, 2H), 3.60 (q, J = 7.2 Hz, 1H), 1.56 (d, J = 7.2 Hz, 3H).

^{13}C NMR (126 MHz, $CDCl_3$) δ: 174.14, 141.40, 138.43, 129.10, 128.76, 127.81, 127.60, 127.50, 127.47, 47.34, 43.71, 18.70.

IR: ν 3308, 3033, 2970, 2926, 1637, 1538.

HRMS (ESI-TOF) *m/z*: [M+H$^+$] calculated for $C_{16}H_{18}NO$, 240.1388; found, 240.1396.

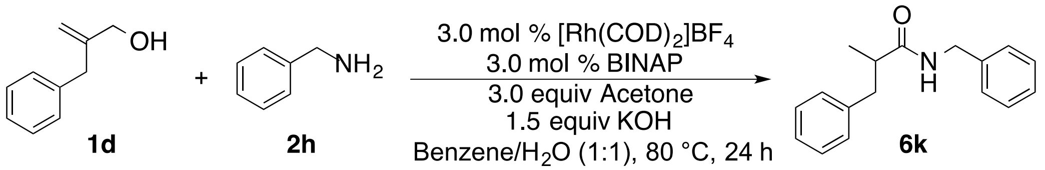

***N*-benzyl-2-methyl-3-phenylpropanamide (Table 2, 6k):** $[Rh(COD)_2]BF_4$ (3.1 mg, 0.0075 mmol, 3.0 mol %), BINAP (4.7 mg, 0.0075 mmol, 3.0 mol %), and KOH (21 mg, 0.38 mmol, 1.5 equiv) were added to a 4 mL vial equipped with a stir bar. After purging with with nitrogen, to the vial was added sequentially benzene (0.2 mL), benzylamine (82 µL, 0.75 mmol, 3.0 equiv), acetone (54 µL, 0.75 mmol, 3.0 equiv), 2-benzylprop-2-en-1-ol (37 µL, 0.25 mmol, 1.0 equiv) and DI water (0.2 mL). The resulting solution was allowed to stir for 24 h at 80 °C. Then, the reaction vial was cooled to room temperature followed by the addition of diphenylmethane as an internal standard for analysis of the crude reaction mixture. The biphasic solution was added EtOAc, dried over anhydrous $MgSO_4$, and filtered. The solution was concentrated *in vacuo* and then purified by silica gel chromatography (4:1 hexanes/EtOAc) to afford a white solid in 70% yield.

$\mathbf{R_f}$ = 0.2

mp = 84-86 °C (reported mp = 83-84 °C)[13]

^{1}H NMR (500 MHz, $CDCl_3$) δ: 7.35 – 7.12 (m, 8H), 7.10 – 6.95 (m, 2H), 5.44 (bs, 1H), 4.40 (dd, J = 14.8, 6.0 Hz, 1H), 4.28 (dd, J = 14.7, 5.4 Hz, 1H), 2.99 (dd, J = 13.4, 8.7 Hz, 1H), 2.71 (dd, J = 13.4, 6.2 Hz, 1H), 2.46 (dq, J = 8.8, 6.7 Hz, 1H), 1.23 (d, J = 6.8 Hz, 3H).

^{13}C NMR (101 MHz, $CDCl_3$) δ: 175.41, 139.97, 138.26, 129.12, 128.72, 128.58, 127.79, 127.48, 126.42, 44.25, 43.51, 40.69, 18.04.

IR: ν 3283, 3030, 2960, 2930, 1637, 1550.

HRMS (ESI-TOF) *m/z*: $[M+H^+]$ calculated for $C_{17}H_{20}NO$, 254.1545; found, 254.1549.

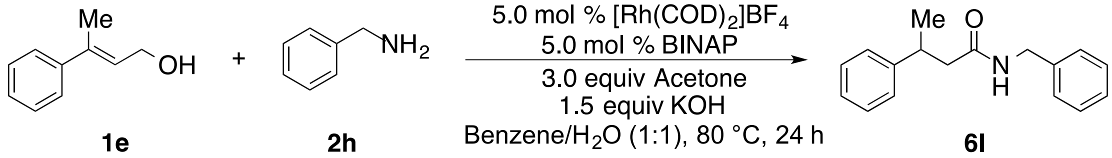

***N*-benzyl-3-phenylbutanamide (Table 2, 6l):** $[Rh(COD)_2]BF_4$ (5.0 mg, 0.0125 mmol, 5.0 mol %) and BINAP (7.8 mg, 0.0125 mmol, 5.0 mol %), and KOH (21 mg, 0.38 mmol, 1.5 equiv) were added to a 4 mL vial equipped with a stir bar. After purging with with nitrogen, to the vial was added sequentially benzene (0.2 mL), benzylamine (82 µL, 0.75 mmol, 3.0 equiv), acetone (54 µL, 0.75 mmol, 3.0 equiv), (*E*)-3-phenylbut-2-en-1-ol (38 µL, 0.25 mmol, 1.0 equiv) and DI water (0.2 mL). The resulting solution was allowed to stir for 24 h at 80 °C. Then, the reaction vial was cooled to room temperature followed by the addition of diphenylmethane as an internal standard for analysis of the crude reaction mixture. The biphasic solution was added EtOAc, dried over anhydrous $MgSO_4$, and filtered. The solution was concentrated *in vacuo* and then purified by silica gel chromatography (5:1 hexanes/EtOAc) to afford a colorless liquid in 74 % yield.

$\mathbf{R_f}$ = 0.15

^{1}**H NMR** (400 MHz, $CDCl_3$) δ: 7.35 – 7.22 (m, 8H), 7.13 – 6.83 (m, 2H), 5.66 (bs, 1H), 4.40 (dd, J = 14.8, 6.0 Hz, 1H), 4.30 (dd, J = 14.8, 5.4 Hz, 1H), 3.36 (h, J = 7.2 Hz, 1H), 2.49 (d, J = 7.5 Hz, 2H), 1.35 (d, J = 7.0 Hz, 3H).

^{13}**C NMR** (101 MHz, $CDCl_3$) δ: 171.57, 145.84, 138.19, 128.74, 128.69, 127.68, 127.44, 126.95, 126.56, 45.97, 43.52, 37.21, 21.95.

IR: ν 3289, 3032, 2965, 2920, 1642, 1549.

HRMS (ESI-TOF) *m/z*: [$M+H^+$] calculated for $C_{17}H_{20}NO$, 254.1545; found, 251.1546.

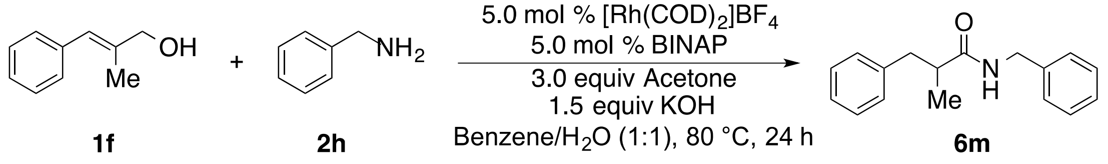

***N*-benzyl-2-methyl-3-phenylpropanamide (Table 2, 6m):** $[Rh(COD)_2]BF_4$ (5.0 mg, 0.0125 mmol, 5.0 mol %) and BINAP (7.8 mg, 0.0125 mmol, 5.0 mol %), and KOH (21 mg, 0.38 mmol, 1.5 equiv) were added to a 4 mL vial equipped with a stir bar. After purging with with nitrogen, to the vial was added sequentially benzene (0.2 mL), benzylamine (82 µL, 0.75 mmol, 3.0 equiv), acetone (54 µL, 0.75 mmol, 3.0 equiv), (*E*)-2-methyl-3-phenylprop-2-en-1-ol (36 µL, 0.25 mmol, 1.0 equiv) and DI water (0.2 mL). The resulting solution was allowed to stir for 24 h at 80 °C. Then, the reaction vial was cooled to room temperature followed by the addition of diphenylmethane as an internal standard for analysis of the crude reaction mixture. The biphasic solution was added EtOAc, dried over anhydrous $MgSO_4$, and filtered. The solution was concentrated *in vacuo* and then purified by silica gel chromatography (4:1 hexanes/EtOAc) to afford a white solid in 60 % yield.

$\mathbf{R_f}$ = 0.1

mp = 88-89 °C (reported mp = 83-84 °C)[18]

^{1}**H NMR** (400 MHz, $CDCl_3$) δ: 7.32 – 7.21 (m, 6H), 7.21 – 7.14 (m, 2H), 7.06 – 6.99 (m, 2H), 5.44 (bs, 1H), 4.40 (dd, J = 14.8, 6.0 Hz, 1H), 4.28 (dd, J = 14.7, 5.3 Hz, 1H), 2.99 (dd, J = 13.4, 8.8 Hz, 1H), 2.71 (dd, J = 13.4, 6.2 Hz, 1H), 2.46 (dp, J = 8.9, 6.7 Hz, 1H), 1.23 (d, J = 6.8 Hz, 3H).

^{13}**C NMR** (101 MHz, $CDCl_3$) δ: 175.43, 139.95, 138.25, 129.10, 128.69, 128.56, 127.76, 127.45, 126.39, 44.19, 43.47, 40.66, 18.02.

IR: ν 3284, 3030, 2960, 1637, 1549.

HRMS (ESI-TOF) *m/z*: [$M+H^+$] calculated for $C_{17}H_{20}NO$, 254.1545; found, 251.1547.

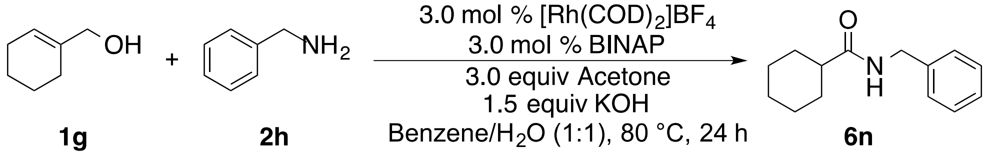

***N*-benzylcyclohexanecarboxamide (Table 2, 6n):** $[Rh(COD)_2]BF_4$ (3.1 mg, 0.0075 mmol, 3.0 mol %), BINAP (4.7 mg, 0.0075 mmol, 3.0 mol %), and KOH (21 mg, 0.38 mmol, 1.5 equiv) were added to a 4 mL vial equipped with a stir bar. After purging with with nitrogen, to the vial was added sequentially benzene (0.2 mL), benzylamine (82 µL, 0.75 mmol, 3.0 equiv), acetone (54 µL, 0.75 mmol, 3.0 equiv), cyclohex-1-en-1-ylmethanol (29 µL, 0.25 mmol, 1.0 equiv) and DI water

(0.2 mL). The resulting solution was allowed to stir for 24 h at 80 °C. Then, the reaction vial was cooled to room temperature followed by the addition of diphenylmethane as an internal standard for analysis of the crude reaction mixture. The biphasic solution was added EtOAc, dried over anhydrous $MgSO_4$, and filtered. The solution was concentrated *in vacuo* and then purified by silica gel chromatography (4:1 hexanes/EtOAc) to afford a white solid in 67% yield.

R_f = 0.4

mp = 109-110 °C (reported mp = 107-109 °C)[14]

^{1}H NMR (400 MHz, $CDCl_3$) δ: 7.38 – 7.30 (m, 2H), 7.30 – 7.26 (m, 3H), 5.70 (bs, 1H), 4.44 (d, J = 5.6 Hz, 2H), 2.11 (tt, J = 11.8, 3.5 Hz, 1H), 1.94 – 1.85 (m, 2H), 1.83 – 1.76 (m, 2H), 1.71 – 1.63 (m, 1H), 1.47 (qd, J = 12.0, 3.3 Hz, 2H), 1.34 – 1.16 (m, 3H).

^{13}C NMR (101 MHz, $CDCl_3$) δ: 176.00, 138.66, 128.83, 127.87, 127.59, 45.73, 43.52, 29.87, 25.88 (overlap).

IR: ν 3285, 2927, 2853, 1641, 1550.

HRMS (ESI-TOF) *m/z*: [M+H$^+$] calculated for $C_{14}H_{20}NO$, 218.1545; found, 218.1546.

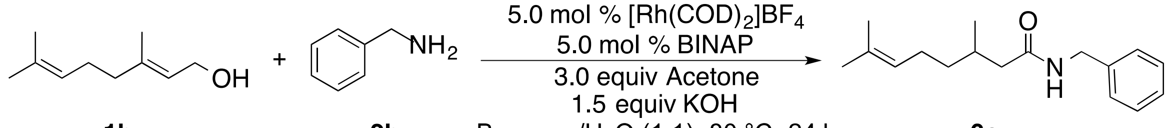

***N*-benzyl-3,7-dimethyloct-6-enamide (Table 2, 6o):** $[Rh(COD)_2]BF_4$ (5.0 mg, 0.0125 mmol, 5.0 mol %) and BINAP (7.8 mg, 0.0125 mmol, 5.0 mol %), and KOH (21 mg, 0.38 mmol, 1.5 equiv) were added to a 4 mL vial equipped with a stir bar. After purging with with nitrogen, to the vial was added sequentially benzene (0.2 mL), benzylamine (82 µL, 0.75 mmol, 3.0 equiv), acetone (54 µL, 0.75 mmol, 3.0 equiv), genaniol (44 µL, 0.25 mmol, 1.0 equiv) and DI water (0.2 mL). The resulting solution was allowed to stir for 24 h at 80 °C. Then, the reaction vial was cooled to room temperature followed by the addition of diphenylmethane as an internal standard for analysis of the crude reaction mixture. The biphasic solution was added EtOAc, dried over anhydrous $MgSO_4$, and filtered. The solution was concentrated *in vacuo* and then purified by silica gel chromatography (4:1 hexanes/EtOAc) to afford a white solid in 65 % yield.

R_f = 0.2

mp = 39-42 °C (reported mp = 43-44 °C)[15]

^{1}H NMR (400 MHz, $CDCl_3$) δ: 7.38 – 7.31 (m, 2H), 7.30 – 7.26 (m, 3H), 5.68 (br, 1H), 5.27 – 4.85 (m, 1H), 4.53 – 4.36 (m, 2H), 2.27 – 2.21 (m, 1H), 2.10 – 1.91 (m, 4H), 1.67 (d, J = 1.2 Hz, 3H), 1.59 (d, J = 1.4 Hz, 3H), 1.43 – 1.33 (m, 1H), 1.24 – 1.15 (m, 1H), 0.95 (d, J = 6.2 Hz, 3H).

^{13}C NMR (101 MHz, $CDCl_3$) δ: 172.50, 138.54, 131.68, 128.85, 128.01, 127.65, 124.45, 44.69, 43.75, 37.05, 30.65, 25.86, 25.59, 19.72, 17.81.

IR: ν 3285, 2967, 2920, 2856, 1633, 1550.

HRMS (ESI-TOF) *m/z*: [M+H$^+$] calculated for $C_{17}H_{26}NO$, 260.2014; found, 260.2012.

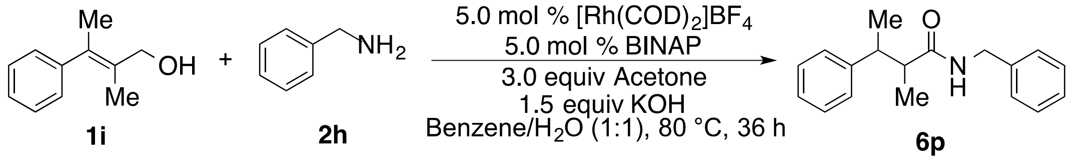

***N*-benzyl-2-methyl-3-phenylbutanamide (Table 2, 6p):** $[Rh(COD)_2]BF_4$ (5.0 mg, 0.0125 mmol, 5.0 mol %) and BINAP (7.8 mg, 0.0125 mmol, 5.0 mol %), and KOH (21 mg, 0.38 mmol, 1.5 equiv) were added to a 4 mL vial equipped with a stir bar. After purging with with nitrogen, to the vial was added sequentially benzene (0.2 mL), benzylamine (82 μL, 0.75 mmol, 3.0 equiv), acetone (54 μL, 0.75 mmol, 3.0 equiv), (*E*)-2-methyl-3-phenylbut-2-en-1-ol (**1g**, 42 μL, 0.25 mmol, 1.0 equiv) and DI water (0.2 mL). The resulting solution was allowed to stir for 36 h at 80 °C. Then, the reaction vial was cooled to room temperature followed by the addition of diphenylmethane as an internal standard for analysis of the crude reaction mixture. The biphasic solution was added EtOAc, dried over anhydrous $MgSO_4$, and filtered. The solution was concentrated *in vacuo* and then purified by silica gel chromatography (6:1 hexanes/EtOAc) to afford afford two diastereomers in 41% (d. r. = 1).

$\mathbf{R_f}$ = 0.1

^{1}H NMR (500 MHz, $CDCl_3$) δ **diastereomer I:** 7.32 – 7.27 (m, 3H), 7.25 – 7.16 (m, 5H), 6.85 – 6.72 (m, 2H), 5.22 (bs, 1H), 4.26 (dd, J = 14.8, 6.3 Hz, 1H), 4.09 (dd, J = 14.8, 5.0 Hz, 1H), 3.02 (dq, J = 9.8, 7.1 Hz, 1H), 2.30 (dq, J = 9.8, 6.8 Hz, 1H), 1.30 (d, J = 7.1 Hz, 3H), 1.27 (d, J = 6.8 Hz, 3H); **diastereomer II:** 7.39 – 7.32 (m, 2H), 7.31 – 7.26 (m, 5H), 7.24 – 7.14 (m, 3H), 5.78 – 5.62 (m, 1H), 4.56 – 4.36 (m, 2H), 2.97 (dq, J = 9.9, 6.8 Hz, 1H), 2.30 (dq, J = 9.8, 6.8 Hz, 1H), 1.27 (d, J = 6.9 Hz, 3H), 0.97 (d, J = 6.8 Hz, 3H).

^{13}C NMR (126 MHz, $CDCl_3$) δ **diastereomer I:** 175.32, 145.92, 138.19, 128.66, 128.62, 127.67, 127.47, 127.32, 126.51, 49.72, 43.35, 43.14, 18.66, 15.79; **diastereomer II:** 175.67, 144.91, 138.49, 128.88, 128.59, 128.05, 127.72, 127.69, 126.54, 49.09, 43.73, 43.58, 20.47, 17.07.

IR: ν 3279, 2968, 2932, 2878, 1645.

HRMS (ESI-TOF) *m/z*: [M+H$^+$] calculated for $C_{18}H_{22}NO$, 268.1701; found, 268.1706.

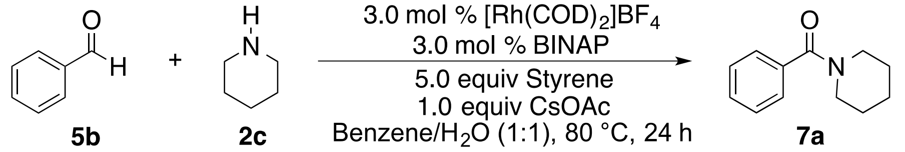

phenyl(piperidin-1-yl)methanone (Table 3, 7a): $[Rh(COD)_2]BF_4$ (3.1 mg, 0.0075 mmol, 3.0 mol %), BINAP (4.7 mg, 0.0075 mmol, 3.0 mol %), and CsOAc (48 mg, 0.25 mmol, 1.0 equiv) were added to a 4 mL vial equipped with a stir bar. After purging with with nitrogen, to the vial was added sequentially benzene (0.2 mL), piperdine (74 μL μL, 0.75 mmol, 3.0 equiv), styrene (145 μL, 1.25 mmol, 5.0 equiv), benzaldehyde (26 μL, 0.25 mmol, 1.0 equiv) and DI water (0.2 mL). The resulting solution was allowed to stir for 24 h at 80 °C. Then, the reaction vial was cooled to room temperature followed by the addition of diphenylmethane as an internal standard for analysis of the crude reaction mixture. The biphasic solution was added EtOAc, dried over anhydrous $MgSO_4$, and filtered. The solution was concentrated *in vacuo* and then purified by silica gel chromatography (2:1 hexanes/EtOAc) to afford a colorless liquid in 91% yield.

R$_f$ = 0.4

^{1}H NMR (400 MHz, $CDCl_3$) δ: 7.39 (m, 5H), 3.81 – 3.60 (m, 2H), 3.42 – 3.24 (m, 2H), 1.82 – 1.40 (m, 6H).

^{13}C NMR (101 MHz, $CDCl_3$) δ: 170.43, 136.60, 129.45, 128.51, 126.89, 48.86, 43.22, 26.65, 25.73, 24.71.

IR: ν 2938, 2857, 1627, 1431.

HRMS (ESI-TOF) *m/z*: [M+H$^+$] calculated for $C_{12}H_{16}NO$, 190.1232; found, 190.1236.

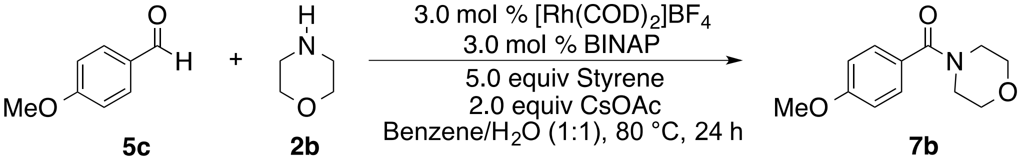

(4-methoxyphenyl)(morpholino)methanone (Table 3, 7b): $[Rh(COD)_2]BF_4$ (3.1 mg, 0.0075 mmol, 3.0 mol %), BINAP (4.7 mg, 0.0075 mmol, 3.0 mol %), and CsOAc (96 mg, 0.50 mmol, 2.0 equiv) were added to a 4 mL vial equipped with a stir bar. After purging with with nitrogen, to the vial was added sequentially benzene (0.2 mL), morpholine (65 μL μL, 0.75 mmol, 3.0 equiv), styrene (145 μL, 1.25 mmol, 5.0 equiv), 4-methoxybenzaldehyde (31 μL, 0.25 mmol, 1.0 equiv) and DI water (0.2 mL). The resulting solution was allowed to stir for 24 h at 80 °C. Then, the reaction vial was cooled to room temperature followed by the addition of diphenylmethane as an internal standard for analysis of the crude reaction mixture. The biphasic solution was added EtOAc, dried over anhydrous $MgSO_4$, and filtered. The solution was concentrated *in vacuo* and then purified by silica gel chromatography (1:1 hexanes/EtOAc) to afford a colorless liquid in 99% yield.

R$_f$ = 0.2

^{1}H NMR (400 MHz, $CDCl_3$) δ: 7.51 – 7.33 (m, 2H), 6.98 – 6.82 (m, 2H), 3.83 (s, 3H), 3.75 – 3.50 (m, 8H).

^{13}C NMR (101 MHz, $CDCl_3$) δ: 170.52, 160.99, 129.31, 127.41, 113.88, 67.04, 55.50, 55.46, 29.81.

IR: ν 2924, 2854, 1629, 1610, 1514.

HRMS (ESI-TOF) *m/z*: [M+H$^+$] calculated for $C_{12}H_{16}NO_3$, 222.1130; found, 222.1133.

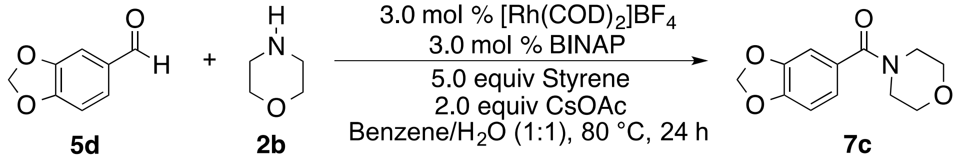

benzo[d][1,3]dioxol-5-yl(morpholino)methanone (Table 3, 7c): $[Rh(COD)_2]BF_4$ (3.1 mg, 0.0075 mmol, 3.0 mol %), BINAP (4.7 mg, 0.0075 mmol, 3.0 mol %), and CsOAc (96 mg, 0.50 mmol, 2.0 equiv) were added to a 4 mL vial equipped with a stir bar. After purging with with nitrogen, to the vial was added sequentially benzene (0.2 mL), morpholine (65 μL, 0.75 mmol, 3.0 equiv), styrene (145 μL, 1.25 mmol, 5.0 equiv), benzo[d][1,3]dioxole-5-carbaldehyde (38 mg, 0.25 mmol, 1.0 equiv) and DI water (0.2 mL). The resulting solution was allowed to stir for 12 h at 80 °C. After 12 h, the reaction vial was cooled to room temperature followed by the addition of diphenylmethane as an internal standard for analysis of the crude reaction mixture. The biphasic solution was added EtOAc, dried over anhydrous $MgSO_4$, and filtered. The solution was concentrated *in vacuo* and then purified by silica gel chromatography (1:1 hexanes/EtOAc) to afford a viscous liquid in 99% yield.

R_f = 0.25

^{1}H NMR (500 MHz, $CDCl_3$) δ: 6.92 (dd, J = 7.9, 1.7 Hz, 1H), 6.90 (d, J = 1.5 Hz, 1H), 6.82 (d, J = 7.9 Hz, 1H), 6.00 (s, 2H), 3.86 – 3.38 (m, 8H).

^{13}C NMR (101 MHz, $CDCl_3$) δ: 170.09, 149.10, 147.80, 128.96, 121.82, 108.40, 108.21, 101.61, 67.02, 48.03, 42.89.

IR: ν 2973, 2925, 2857, 1633, 1440.

HRMS (ESI-TOF) *m/z*: [M+H$^+$] calculated for $C_{12}H_{14}NO_4$, 236.0923; found, 236.0925.

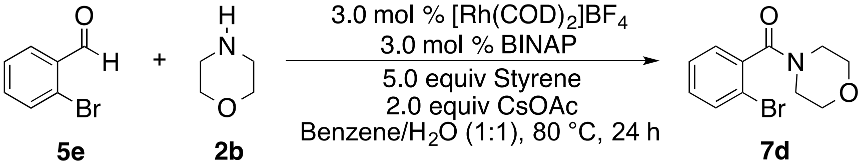

(2-bromophenyl)(morpholino)methanone (Table 3, 7d): $[Rh(COD)_2]BF_4$ (3.1 mg, 0.0075 mmol, 3.0 mol %), BINAP (4.7 mg, 0.0075 mmol, 3.0 mol %), and CsOAc (96 mg, 0.50 mmol, 2.0 equiv) were added to a 4 mL vial equipped with a stir bar. After purging with with nitrogen, to the vial was added sequentially benzene (0.2 mL), morpholine (65 µL, 0.75 mmol, 3.0 equiv), styrene (145 µL, 1.25 mmol, 5.0 equiv), 2-bromobenzaldehyde (29 µL, 0.25 mmol, 1.0 equiv) and DI water (0.2 mL). The resulting solution was allowed to stir for 12 h at 80 °C. After 12 h, the reaction vial was cooled to room temperature followed by the addition of diphenylmethane as an internal standard for analysis of the crude reaction mixture. The biphasic solution was added EtOAc, dried over anhydrous $MgSO_4$, and filtered. The solution was concentrated *in vacuo* and then purified by silica gel chromatography (1:1 hexanes/EtOAc) to afford a white solid in 64% yield.

R_f = 0.2

mp = 85-88 °C

^{1}H NMR (400 MHz, $CDCl_3$) δ: 7.58 (dd, J = 8.4, 1.1 Hz, 1H), 7.37 (td, J = 7.5, 1.2 Hz, 1H), 7.31 – 7.22 (m, 2H), 3.92 – 3.83 (m, 1H), 3.83 – 3.69 (m, 4H), 3.59 (ddd, J = 11.6, 6.2, 3.3 Hz, 1H), 3.29 (ddd, J = 13.4, 6.2, 3.2 Hz, 1H), 3.20 (ddd, J = 13.4, 6.7, 3.3 Hz, 1H).

^{13}C NMR (101 MHz, $CDCl_3$) δ: 167.88, 137.68, 132.98, 130.59, 127.95, 127.87, 119.27, 66.90, 66.81, 47.29, 42.14.

IR: ν 2966, 2927, 2867, 1625, 1427.

HRMS (ESI-TOF) *m/z*: [M+H$^+$] calculated for $C_{11}H_{13}NO_2Br$, 270.0130; found, 270.0135.

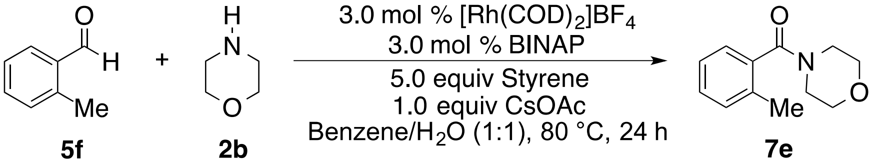

morpholino(o-tolyl)methanone (Table 3, 7e): $[Rh(COD)_2]BF_4$ (3.1 mg, 0.0075 mmol, 3.0 mol %), BINAP (4.7 mg, 0.0075 mmol, 3.0 mol %), and CsOAc (48 mg, 0.25 mmol, 1.0 equiv) were added to a 4 mL vial equipped with a stir bar. After purging with with nitrogen, to the vial was added sequentially benzene (0.2 mL), morpholine (65 µL µL, 0.75 mmol, 3.0 equiv), styrene (145 µL, 1.25 mmol, 5.0 equiv), 2-methylbenzaldehyde (29 µL, 0.25 mmol, 1.0 equiv) and DI water (0.2

mL). The resulting solution was allowed to stir for 24 h at 80 °C. Then, the reaction vial was cooled to room temperature followed by the addition of diphenylmethane as an internal standard for analysis of the crude reaction mixture. The biphasic solution was added EtOAc, dried over anhydrous $MgSO_4$, and filtered. The solution was concentrated *in vacuo* and then purified by silica gel chromatography (1:1 hexanes/EtOAc) to afford a colorless liquid in 86% yield.

R_f = 0.15

^{1}H NMR (400 MHz, $CDCl_3$) δ: 7.32 – 7.26 (m, 1H), 7.25 – 7.13 (m, 3H), 3.92 – 3.73 (m, 4H), 3.65 – 3.51 (m, 2H), 3.31 – 3.18 (m, 2H), 2.32 (s, 3H).

^{13}C NMR (101 MHz, $CDCl_3$) δ: 170.20, 135.72, 134.28, 130.61, 129.16, 126.12, 125.92, 67.12, 67.07, 47.37, 42.02, 19.15.

IR: ν 2923, 2854, 1635, 1427.

HRMS (ESI-TOF) *m/z*: [M+H$^+$] calculated for $C_{12}H_{16}NO_2$, 206.1181; found, 206.1183.

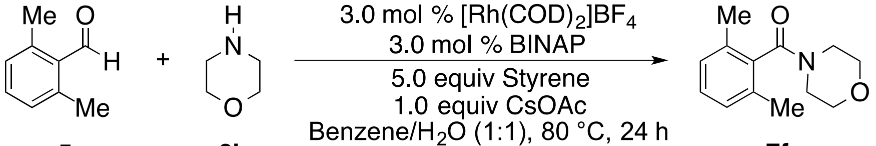

5g **2b** **7f**

(2,6-dimethylphenyl)(morpholino)methanone (Table 3, 7f): $[Rh(COD)_2]BF_4$ (3.1 mg, 0.0075 mmol, 3.0 mol %), BINAP (4.7 mg, 0.0075 mmol, 3.0 mol %), and CsOAc (48 mg, 0.25 mmol, 1.0 equiv) were added to a 4 mL vial equipped with a stir bar. After purging with with nitrogen, to the vial was added sequentially benzene (0.2 mL), morpholine (65 µL µL, 0.75 mmol, 3.0 equiv), styrene (145 µL, 1.25 mmol, 5.0 equiv), 2,6-dimethylbenzaldehyde (34 µL, 0.25 mmol, 1.0 equiv) and DI water (0.2 mL). The resulting solution was allowed to stir for 24 h at 80 °C. Then, the reaction vial was cooled to room temperature followed by the addition of diphenylmethane as an internal standard for analysis of the crude reaction mixture. The biphasic solution was added EtOAc, dried over anhydrous $MgSO_4$, and filtered. The solution was concentrated *in vacuo* and then purified by silica gel chromatography (1:1 hexanes/EtOAc) to afford a colorless liquid in 88% yield.

R_f = 0.2

^{1}H NMR (500 MHz, $CDCl_3$) δ: 7.15 (t, J = 7.6 Hz, 1H), 7.03 (d, J = 7.6 Hz, 2H), 3.85 (dd, J = 5.8, 3.7 Hz, 2H), 3.77 (dd, J = 5.5, 4.1 Hz, 2H), 3.61 – 3.55 (m, 2H), 3.18 (dd, J = 5.5, 4.2 Hz, 2H), 2.26 (s, 6H).

^{13}C NMR (101 MHz, $CDCl_3$) δ: 169.82, 135.65, 133.71, 128.67, 127.71, 67.18, 67.06, 46.49, 41.59, 19.23.

IR: ν 2972, 2925, 2856, 1633, 1435.

HRMS (ESI-TOF) *m/z*: [M+H$^+$] calculated for $C_{13}H_{18}NO_2$, 220.1338; found, 220.1339.

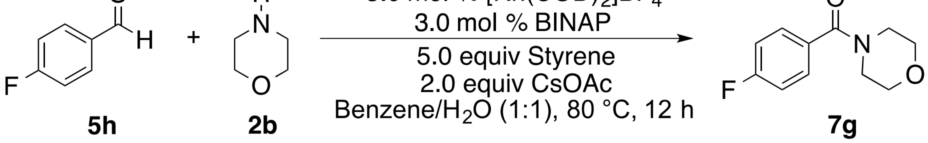

5h **2b** **7g**

(4-fluorophenyl)(morpholino)methanone (Table 3, 7g): $[Rh(COD)_2]BF_4$ (3.1 mg, 0.0075 mmol, 3.0 mol %), BINAP (4.7 mg, 0.0075 mmol, 3.0 mol %), and CsOAc (96 mg, 0.50 mmol, 2.0 equiv) were added to a 4 mL vial equipped with a stir

bar. After purging with with nitrogen, to the vial was added sequentially benzene (0.2 mL), morpholine (65 μL, 0.75 mmol, 3.0 equiv), styrene (145 μL, 1.25 mmol, 5.0 equiv), 4-fluorobenzaldehyde (27 μL, 0.25 mmol, 1.0 equiv) and DI water (0.2 mL). The resulting solution was allowed to stir for 12 h at 80 °C. After 12 h, the reaction vial was cooled to room temperature followed by the addition of diphenylmethane as an internal standard for analysis of the crude reaction mixture. The biphasic solution was added EtOAc, dried over anhydrous $MgSO_4$, and filtered. The solution was concentrated *in vacuo* and then purified by silica gel chromatography (1:1 hexanes/EtOAc) to afford a colorless liquid in 85% yield.

R_f = 0.2

^{1}H NMR (400 MHz, $CDCl_3$) δ: 7.47 – 7.38 (m, 2H), 7.16 – 7.05 (m, 2H), 4.08 – 3.02 (m, 8H).

^{13}C NMR (101 MHz, $CDCl_3$) δ: 169.63, 163.59 (d, J_{CF} = 250.1 Hz), 131.39, 129.56 (d, J_{CF}= 8.7 Hz), 115.79 (d, J_{CF} = 21.8 Hz), 66.97, 48.05, 42.61.

^{19}F NMR (470 MHz, $CDCl_3$) δ: -110.92 (t, J_{CF} = 6.8 Hz).

IR: ν 2974, 2928, 2960, 1631, 1619, 1511.

HRMS (ESI-TOF) *m/z*: [M+H$^+$] calculated for $C_{11}H_{13}NO_2F$, 210.0930; found, 210.0931.

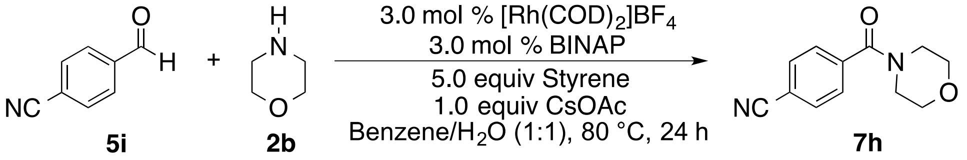

4-(morpholine-4-carbonyl)benzonitrile (Table 3, 7h): $[Rh(COD)_2]BF_4$ (3.1 mg, 0.0075 mmol, 3.0 mol %), BINAP (4.7 mg, 0.0075 mmol, 3.0 mol %), and CsOAc (48 mg, 0.25 mmol, 1.0 equiv) were added to a 4 mL vial equipped with a stir bar. After purging with with nitrogen, to the vial was added sequentially benzene (0.2 mL), morpholine (65 μL, 0.75 mmol, 3.0 equiv), styrene (145 μL, 1.25 mmol, 5.0 equiv), 4-formylbenzonitrile (33 mg, 0.25 mmol, 1.0 equiv) and DI water (0.2 mL). The resulting solution was allowed to stir for 12 h at 80 °C. After 12 h, the reaction vial was cooled to room temperature followed by the addition of diphenylmethane as an internal standard for analysis of the crude reaction mixture. The biphasic solution was added EtOAc, dried over anhydrous $MgSO_4$, and filtered. The solution was concentrated *in vacuo* and then purified by silica gel chromatography (1:1 hexanes/EtOAc) to afford a light yellow solid in 48% yield.

R_f = 0.2

mp = 138-140 °C (reported mp = 139-140 °C)[16]

^{1}H NMR (400 MHz, $CDCl_3$) δ: 7.79 – 7.68 (m, 2H), 7.57 – 7.47 (m, 2H), 3.88 – 3.55 (m, 6H), 3.46 – 3.32 (m, 2H).

^{13}C NMR (101 MHz, $CDCl_3$) δ: 168.45, 139.77, 132.66, 127.96, 118.13, 113.90, 66.89, 48.12, 42.67.

IR: ν 2927, 2863, 2227, 1621, 1441.

HRMS (ESI-TOF) *m/z*: [M+H$^+$] calculated for $C_{12}H_{13}N_2O_2$, 217.0977; found, 217.0983.

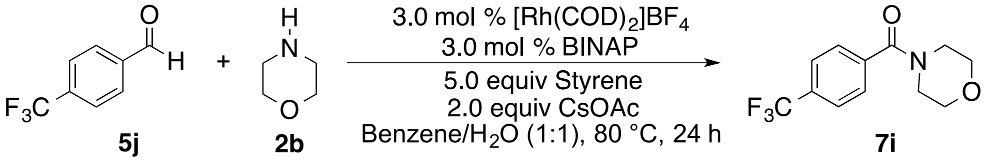

morpholino(4-(trifluoromethyl)phenyl)methanone (Table 3, 7i): $[Rh(COD)_2]BF_4$ (3.1 mg, 0.0075 mmol, 3.0 mol %), BINAP (4.7 mg, 0.0075 mmol, 3.0 mol %), and CsOAc (96 mg, 0.50 mmol, 2.0 equiv) were added to a 4 mL vial equipped with a stir bar. After purging with with nitrogen, to the vial was added sequentially benzene (0.2 mL), morpholine (65 µL, 0.75 mmol, 3.0 equiv), styrene (145 µL, 1.25 mmol, 5.0 equiv), 4-(trifluoromethyl)benzaldehyde (34 µL, 0.25 mmol, 1.0 equiv) and DI water (0.2 mL). The resulting solution was allowed to stir for 12 h at 80 °C. After 12 h, the reaction vial was cooled to room temperature followed by the addition of diphenylmethane as an internal standard for analysis of the crude reaction mixture. The biphasic solution was added EtOAc, dried over anhydrous $MgSO_4$, and filtered. The solution was concentrated *in vacuo* and then purified by silica gel chromatography (1:1 hexanes/EtOAc) to afford a colorless liquid in 72% yield.

$\mathbf{R_f}$ = 0.2

^{1}H NMR (400 MHz, $CDCl_3$) δ: 7.69 (d, J = 8.0 Hz, 2H), 7.52 (d, J = 8.0 Hz, 2H), 3.88 – 3.57 (m, 6H), 3.53 – 3.26 (m, 2H).

^{13}C NMR (101 MHz, $CDCl_3$) δ: 169.14, 139.07, 132.07 (q, J_{CF} = 32.8 Hz), 127.69, 125.92 (q, J_{CF} = 3.8 Hz), 123.86 (q, J_{CF} = 272.5 Hz), 67.02, 48.31, 42.76.

^{19}F NMR (470 MHz, $CDCl_3$) δ: 62.98 (s).

IR: ν 2974, 2924, 2864, 1637, 1434.

HRMS (ESI-TOF) *m/z*: $[M+H^+]$ calculated for $C_{12}H_{13}NO_2F_3$, 260.0898; found, 260.0905.

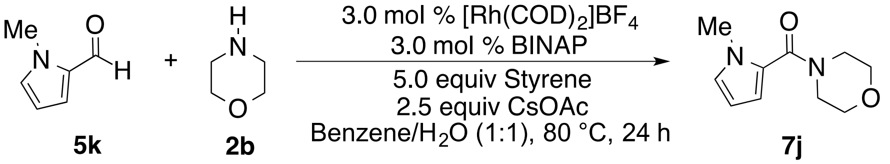

(1-methyl-1H-pyrrol-2-yl)(morpholino)methanone (Table 3, 7j): $[Rh(COD)_2]BF_4$ (3.1 mg, 0.0075 mmol, 3.0 mol %), BINAP (4.7 mg, 0.0075 mmol, 3.0 mol %), and CsOAc (120 mg, 1.25 mmol, 2.5 equiv) were added to a 4 mL vial equipped with a stir bar. After purging with with nitrogen, to the vial was added sequentially benzene (0.2 mL), morpholine (65 µL, 0.75 mmol, 3.0 equiv), styrene (145 µL, 1.25 mmol, 5.0 equiv), 1-methyl-1H-pyrrole-2-carbaldehyde (27 µL, 0.25 mmol, 1.0 equiv) and DI water (0.2 mL). The resulting solution was allowed to stir for 12 h at 80 °C. After 12 h, the reaction vial was cooled to room temperature followed by the addition of diphenylmethane as an internal standard for analysis of the crude reaction mixture. The biphasic solution was added EtOAc, dried over anhydrous $MgSO_4$, and filtered. The solution was concentrated *in vacuo* and then purified by silica gel chromatography (1:1 hexanes/EtOAc) to afford a light yellow solid in 97% yield.

$\mathbf{R_f}$ = 0.3

mp =80-83 °C

^{1}H NMR (400 MHz, C_6D_6) δ: 6.28 (t, J = 2.1 Hz, 1H), 6.21 (dd, J = 3.9, 1.7 Hz, 1H), 6.10 (dd, J = 3.8, 2.6 Hz, 1H), 3.47 (s, 3H), 3.43 (dd, J = 5.6, 4.0 Hz, 4H), 3.27 – 3.19 (m, 4H).

^{13}C NMR (101 MHz, $CDCl_3$) δ: 163.21, 126.75, 124.77, 113.25, 107.13, 67.25, 45.87, 35.94.

IR: ν 2968, 2930, 2863, 1602, 1534.

HRMS (ESI-TOF) *m/z*: [M+H$^+$] calculated for $C_{12}H_{13}N_2O_2$, 195.1134; found, 195.1131.

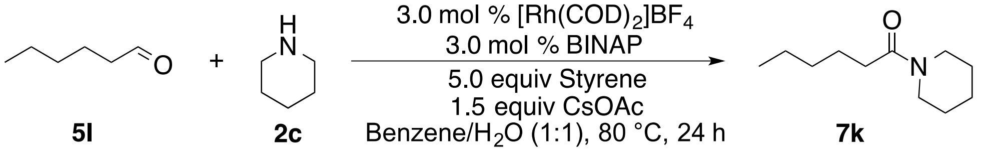

1-(piperidin-1-yl)hexan-1-one (Table 3, 7k): $[Rh(COD)_2]BF_4$ (3.1 mg, 0.0075 mmol, 3.0 mol %), BINAP (4.7 mg, 0.0075 mmol, 3.0 mol %), and CsOAc (72 mg, 0.38 mmol, 1.5 equiv) were added to a 4 mL vial equipped with a stir bar. After purging with with nitrogen, to the vial was added sequentially benzene (0.2 mL), piperdine (74 μL, 0.75 mmol, 3.0 equiv), styrene (145 μL, 1.25 mmol, 5.0 equiv), hexanal (34 μL, 0.25 mmol, 1.0 equiv) and DI water (0.2 mL). The resulting solution was allowed to stir for 24 h at 80 °C. Then, the reaction vial was cooled to room temperature followed by the addition of diphenylmethane as an internal standard for analysis of the crude reaction mixture. The biphasic solution was added EtOAc, dried over anhydrous $MgSO_4$, and filtered. The solution was concentrated *in vacuo* and then purified by silica gel chromatography (3:1 hexanes/EtOAc) to afford a light yellow liquid in 63% yield.

R_f = 0.2

^{1}H NMR (400 MHz, $CDCl_3$) δ: 6.54 – 6.39 (m, 4H), 2.30 (td, J = 7.8, 1.5 Hz, 2H), 1.66 – 1.57 (m, 4H), 1.57 – 1.48 (m, 4H), 1.39 – 1.26 (m, 4H), 0.88 (t, J = 6.8 Hz, 3H).

^{13}C NMR (126 MHz, $CDCl_3$) δ: 171.66, 46.84, 42.70, 33.58, 31.84, 26.70, 25.72, 25.32, 24.72, 22.62, 14.10.

IR: ν 2934, 2857, 2960, 1641, 1435.

HRMS (ESI-TOF) *m/z*: [M+H$^+$] calculated for $C_{11}H_{22}NO$, 184.1701; found, 184.1705.

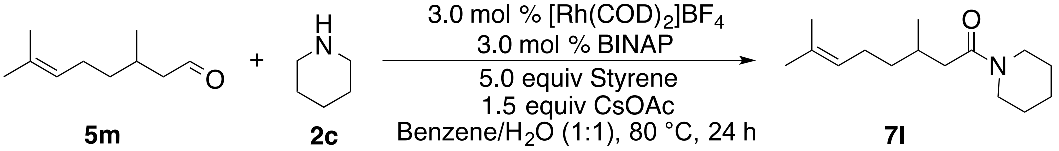

3,7-dimethyl-1-(piperidin-1-yl)oct-6-en-1-one (Table 3, 7l): $[Rh(COD)_2]BF_4$ (3.1 mg, 0.0075 mmol, 3.0 mol %), BINAP (4.7 mg, 0.0075 mmol, 3.0 mol %), and CsOAc (72 mg, 0.38 mmol, 1.5 equiv) were added to a 4 mL vial equipped with a stir bar. After purging with with nitrogen, to the vial was added sequentially benzene (0.2 mL), piperdine (74 μL, 0.75 mmol, 3.0 equiv), styrene (145 μL, 1.25 mmol, 5.0 equiv), 3,7-dimethyloct-6-enal (45 μL, 0.25 mmol, 1.0 equiv) and DI water (0.2 mL). The resulting solution was allowed to stir for 24 h at 80 °C. Then, the reaction vial was cooled to room temperature followed by the addition of diphenylmethane as an internal standard for analysis of the crude reaction mixture. The biphasic solution was added EtOAc, dried over anhydrous $MgSO_4$, and filtered. The solution was concentrated *in vacuo* and then purified by silica gel chromatography (4:1 hexanes/EtOAc) to afford a colorless liquid in 78% yield.

R_f = 0.35

^{1}H NMR (400 MHz, $CDCl_3$) δ: 5.23 – 4.88 (m, 1H), 3.55 (br, 2H), 3.39 (br, 2H), 2.32 (dd, J = 14.3, 5.9 Hz, 1H), 2.12 (dd, J = 14.3, 8.4 Hz, 1H), 2.08 – 1.90 (m, 3H), 1.67 (t, J = 1.3 Hz, 3H), 1.65 – 1.61 (m, 1H), 1.60 (d, J = 1.3 Hz, 3H), 1.57 – 1.48 (m, 4H), 1.38 (dddd, J = 13.4, 9.3, 6.5, 5.4 Hz, 1H), 1.21 (dddd, J = 13.6, 9.3, 7.9, 5.8 Hz, 1H), 0.94 (d, J = 6.5 Hz, 3H).

^{13}C NMR (101 MHz, $CDCl_3$) δ: 171.07, 131.51, 124.65, 47.09, 42.79, 40.85, 37.27, 30.26, 29.83, 26.76, 25.83, 25.66, 24.75, 19.95, 17.82.

IR: ν 2929, 2856, 1642, 1443.

HRMS (ESI-TOF) *m/z*: [M+H$^+$] calculated for $C_{15}H_{28}NO$, 238.2171; found, 238.2179.

H. Reference:

(1) Chiang, P.-C.; Kim, Y.; Bode, J. W. *Chem. Commun*. **2009**, *45*, 4566-4568.

(2) Allen, C. L.; Chhatwal, R.; Williams, J. M. J. *Chem. Commun*. **2012**, *48*, 666-668.

(3) Funasaka, S.; Mukaiyama, T. *Bull. Chem. Soc. Jpn*. **2008**, *81*, 148-159.

(4) Ohshima, T.; Iwasaki, T.; Maegawa, Y.; Yoshiyama, A.; Mashima, K. *J. Am. Chem. Soc*. **2008**, *130*, 2944-2945.

(5) Liu, H.; Laurenczy, G.; Yan, N.; Dyson, P. J. *Chem. Commun*. **2014**, *50*, 341-343.

(6) Badr, M. Z. A.; Aly, M. M.; Abdel-Latif, F. F. *J. Org. Chem*. **1979**, *44*, 3244-3247.

(7) Hanada, S.; Tsutsumi, E.; Motoyama, Y.; Nagashima, H. *J. Am. Chem. Soc*. **2009**, *131*, 15032-15040.

(8) Tamura, M.; Murase, D.; Komura, K. *Synthesis*, **2015**, *47*, 769-776.

(9) Szostak, M.; Spain, M.; Eberhart, A. J.; Procter, D. J. *J. Am. Chem. Soc*. **2014**, *136*, 2268-2271.

(10) Watson, A. J. A.; Wakeham, R. J.; Maxwell, A. C. Williams, J. M. J. *Tetrahedron*, **2014**, *70*, 3683-3690.

(11) Morlino, V. J.; Martin, R. B. *J. Am. Chem. Soc*. **1967**, *89*, 3107-3111.

(12) Shiina, I.; Suenaga, Y.; Nakano, M.; Mukaiyama, T. *Bull. Chem. Soc. Jpn*. **2000**, *73*, 2811-2818.

(13) Davies, S. G.; Dixon, D. J. *J. Chem. Soc., Perkin Trans*. **2002**, *1*, 1869-1876.

(14) Saito, Y.; Ouchi, H.; Takahata, H. *Tetrahedron*, **2008**, *64*, 11129-11135.

(15) Brodney, M. A.; Padwa, A. *J. Org. Chem*. **1999**, *64*, 556-565.

(16) Tamaru, Y.; Yamada, Y.; Yoshida, Z.-i. *Synthesis*, **1983**, 474-476.

(17) Sidhu, G. S.; Sattur, P. B.; Sadanandam, Y. S. *Arch. Pharmaz*. **1972**, 54-57.

(18) Davies, S. G.; Dixon, D. J. *J. Chem. Soc., Perkin, Trans. I*, **2002**, 1869-1876.
